# Small molecules rescue pathogenic POLGA aggregation and restore its functions in a mouse model of PEO

**DOI:** 10.1038/s44321-026-00532-3

**Published:** 2026-10-06

**Authors:** Aftab Mohammed, Tavleen Kaur, Priyanka Panwar, Samanwita Ghosh, Hanif Ahmad, Sadananda Kumbhakar, Meltem Muftuoglu, Carlo Viscomi, Sagar Sengupta

**Affiliations:** 1 Biotechnology Research and Innovation Council—National Institute of Immunology (BRIC-NII), Aruna Asaf Ali Marg, New Delhi, India; 2 Biotechnology Research and Innovation Council—National Institute of Biomedical Genomics (BRIC-NIBMG), Kalyani, India; 3 Regional Centre for Biotechnology (RCB), 3rd Milestone, Faridabad-Gurgaon Expressway, NCR Biotech Cluster, Faridabad, Haryana 121001 India; 4 Department of Molecular Biology and Genetics, Faculty of Engineering and Natural Sciences, Acibadem Mehmet Ali Aydinlar University, Atasehir, 34638 Istanbul, Turkey; 5 Department of Biosciences, University of Milano, Via Celoria, 26, 20133 Milano, Italy; 6 Veneto Institute of Molecular Medicine (VIMM), Via Orus, 2, 35129 Padova, Italy

**Keywords:** Genetics, Gene Therapy & Genetic Disease, Organelles, Pharmacology & Drug Discovery

## Abstract

POLGA, the catalytic subunit of the mitochondrial DNA polymerase, is essential for mitochondrial DNA (mtDNA) replication and maintenance. Mutations in POLGA cause progressive external ophthalmoplegia (PEO), but the underlying pathogenic mechanisms remain unclear. Here, we show that the mitochondrial E3 ligase MITOL hyperubiquitylates PEO-associated POLGA mutants at lysine 1060, triggering conformational changes and formation of insoluble aggregates that impair their mitochondrial import and consequently compromise mtDNA replication and mitochondrial homeostasis. To identify compounds that disrupt POLGA aggregates and restore mutant POLGA function, we screened an FDA/EMA/PMDA-approved drug library using a split-GFP system. We identified two clinically approved compounds, diltiazem hydrochloride (C1) and sulbactam (C4), that disrupted POLGA aggregates, prevented aberrant MITOL recognition, restored mitochondrial import, and rescued mtDNA replication and mitochondrial function. Importantly, treatment with either compound significantly improved motor performance in a knock-in mouse model carrying the disease-relevant A467T-equivalent POLGA mutation. These findings establish pathogenic aggregation as a previously underrecognized disease mechanism and demonstrate that disrupting POLGA aggregates with repurposed drugs can restore mitochondrial function in vivo, providing a potential therapeutic strategy for POLGA-associated mitochondrial disease.

## Synopsis

A set of PEO mutants forms aggregates and accumulates on the mitochondrial surface. Sulbactam is identified as a small-molecule disruptor of PEO mutant aggregates and restores their mitochondrial import, resulting in enhanced mitochondrial homeostasis in the PEO mouse model.

- A set of PEO mutants forms aggregates and show decreased mitochondrial import.
- Lack of mitochondrial import results in decreased mtDNA content, resulting in enhanced mitophagy.
- Sulbactam disrupts PEO aggregates and restores mtDNA replication and mitochondrial homeostasis.
- Sulbactam improved motor functions in 7-week-old PEO mice.

## Abstract (web summary)

A set of PEO mutants forms aggregates and accumulates on the mitochondrial surface. Sulbactam is identified as a small-molecule disruptor of PEO mutant aggregates and restores their mitochondrial import, resulting in enhanced mitochondrial homeostasis in the PEO mouse model.

## Graphical abstract

**Figure:**
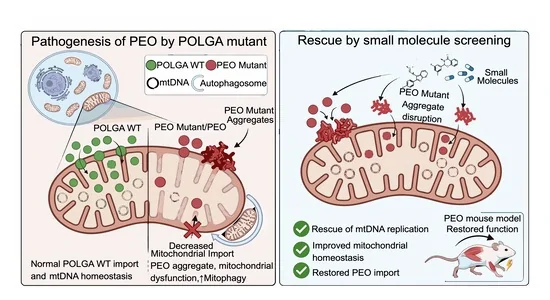

The paper explained **Problem**

Mutations in POLGA, a key regulator of mtDNA replication, can cause PEO and mitochondrial dysfunction. Currently, there are no therapies for PEO, and treatment largely focuses on managing symptoms. Understanding the molecular mechanisms driving mitochondrial dysfunction is therefore critical for developing targeted treatments.

**Results**

In this study, we found that PEO-associated POLGA mutants form aggregates and exhibit impaired mitochondrial import, leading to disrupted mitochondrial homoeostasis. Small-molecule screening identified compounds that disrupted these aggregates and rescued the mutant phenotype. The treatment restored mitochondrial import of PEO-associated POLGA mutants and improved mitochondrial DNA replication. These compounds also improved mitochondrial homoeostasis in mice, thereby reducing the consequences of mitochondrial dysfunction.

**Impact**

These findings identify PEO-associated POLGA mutant aggregates as a druggable mechanism contributing to PEO pathology. Disrupting aggregates can restore PEO mutant mitochondrial import and improve mitochondrial homoeostasis. The findings provide a promising foundation for developing small-molecule therapies for PEO and related mitochondrial diseases.

## Introduction

The mitochondrial polymerase gamma holoenzyme consists of a monomeric catalytic subunit, DNA polymerase gamma A (POLGA), and a dimeric accessory subunit, DNA polymerase gamma B (POLGB), which enhances the enzyme’s processivity and affinity for DNA (Graziewicz et al, 2006; Pinz & Bogenhagen, 2006; Yakubovskaya et al, 2006). As the major mtDNA replicative polymerase, POLGA is critical for maintaining mtDNA integrity, which is vital for oxidative phosphorylation and ATP production. Mutations in POLGA can lead to a range of disorders and can impair its enzymatic activity and disrupt its interaction with POLGB, leading to increased susceptibility to degradation by LONP1, the main protease in the mitochondrial matrix (Silva-Pinheiro et al, 2021; Van Goethem et al, 2001). Patients with POLGA mutations are characterised by ptosis, progressive external ophthalmoplegia (PEO), and ataxia and have a huge variability of phenotypes (Chan and Copeland, 2009; Hikmat et al, 2020; Lee et al, 2009; Saneto and Naviaux, 2010).

Ubiquitylation plays a crucial role as a regulatory mechanism for mitochondrial protein import. By balancing the removal of ubiquitin on mitochondrial proteins, the cell ensures proper mitochondrial protein import (Eldeeb et al, 2020; Phu et al, 2020). Recent findings have shown that mitochondrial E3 ligase MITOL-mediated ubiquitylation prevents multiple proteins involved in mtDNA replication, like POLGA (Hussain et al, 2021) and RECQL4 (Hussain et al, 2023), from entering mitochondria by reducing their interaction with the translocase Tom20. PEO-associated POLG mutants (hereafter called PEO mutants) show enhanced MITOL-dependent ubiquitylation, thereby accumulating on the mitochondrial surface and preventing their optimal mitochondrial import (Hussain et al, 2021).

Here, we show that multiple PEO mutants undergo conformational change and form aggregates. These PEO mutants are hyperubiquitylated by MITOL, which negatively regulates their mitochondrial entry and causes decreased mtDNA replication and altered mitochondrial homoeostasis. With the aim of restoring mitochondrial import and making the PEO mutants functional again, we screened the Prestwick library consisting of 1280 small molecules, 98% being marketed-approved drugs (mostly FDA-approved, several approved in Europe (EMA) and Japan (PMDA). We identified two small molecules, diltiazem hydrochloride (C1) and Sulbactam (C4), which induced the conformational change in PEO mutants, reversed their hyperubiquitylation by MITOL, leading to the disruption of their aggregates. This culminated in their enhanced mitochondrial entry and allowed the reinitiation of mtDNA synthesis. The effects of C1 and C4 were observed not only in cells expressing PEO mutants but also in a knockin PEO mice model. These mice treated with C1 and C4 regained their motor functions to an appreciable extent, thereby indicating the use of these small molecules as a promising therapeutic strategy for PEO patients.

## Results

### Hyperubiquitylated PEO mutants form aggregate-like structures and induce mitophagy

**Figure EV1:**
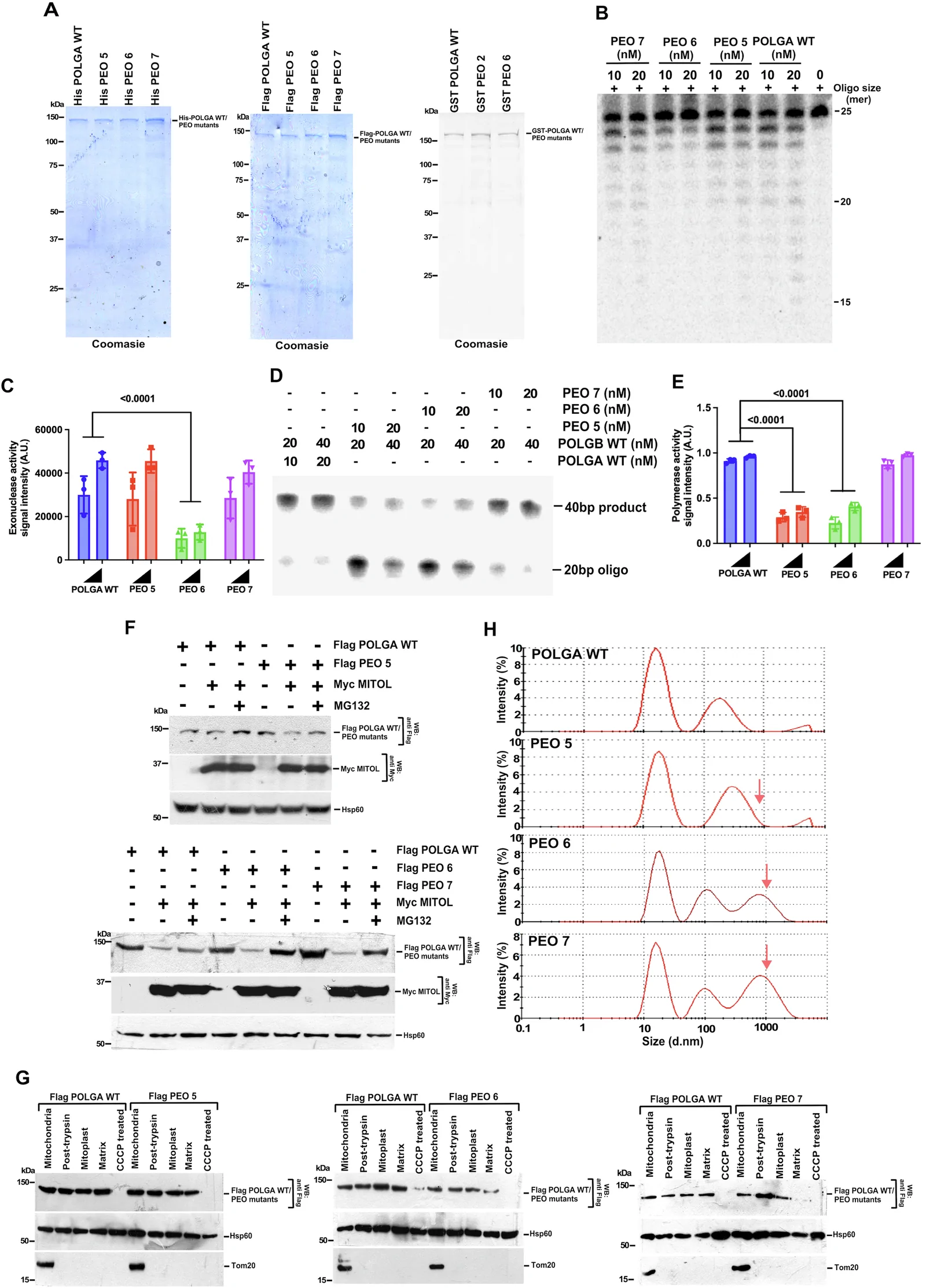
PEO mutants show differential DNA polymerase and exonuclease activity in vitro and form aggregates. (**A**) Purity of proteins used in assays. Coomassie gel showing the purity of the recombinant His-tagged (left, used for in vitro exonuclease and primer extension assay), Flag-tagged (middle, used for import assay and DLS) and GST-tagged (right, used for tryptophan fluorescence assay and DLS) POLGA WT and PEO mutants. Three independent preparations were used for each set of tagged proteins. (**B**,** C**) PEO 6 shows decreased exonuclease activity. (**B**) In vitro exonuclease assay was carried out with His-tagged POLGA WT, PEO 5, PEO 6 and PEO 7 using a paired template labelled with ATP [γ-^32^P]. (**C**) Quantitation of (**B**). AU is arbitrary units. *n* = 3 biological replicates. (**D**,** E**) PEO 5 and PEO 6 show minimal primer extension activity. (**D**) In vitro primer extension assay was performed with His-tagged PEO mutants and POLGA WT using an annealed template labelled with ATP [γ-^32^P]. (**E**) Quantitation of (**D**). *n* = 3 biological replicates. (**F**) PEO mutants undergo enhanced proteasomal degradation. Cell extracts were made from HEK293T cells (in ±MG132 conditions) transiently expressing Flag-tagged POLGA WT, PEO 5, PEO 6 and PEO 7 mutants, along with Myc-tagged MITOL. Western analysis was done with **t**he indicated antibodies. *n* = 3 biological replicates. (**G**) PEO mutants show decreased mitochondrial import. An in vitro import assay was done using Flag-tagged POLGA WT, PEO 5, PEO 6 and PEO 7 mutants. Post-incubation, each of the indicated mitochondrial fractions was subjected to Western blot analysis with the indicated antibodies. n = 3 biological replicates. (**H**) PEO 5, PEO 6 and PEO 7 mutants form high-order structures. Flag-tagged POLGA WT, PEO 5, PEO 6 and PEO 7 proteins were monitored by DLS and represented by an intensity plot. *n* = 3 biological replicates. Data represent the mean ± SD and were analysed using a two-way ANOVA (for **C**, **E**) statistical test with Tukey’s (**C**) and Dunnett’s (**E**) multiple comparisons tests.

**Figure 1:**
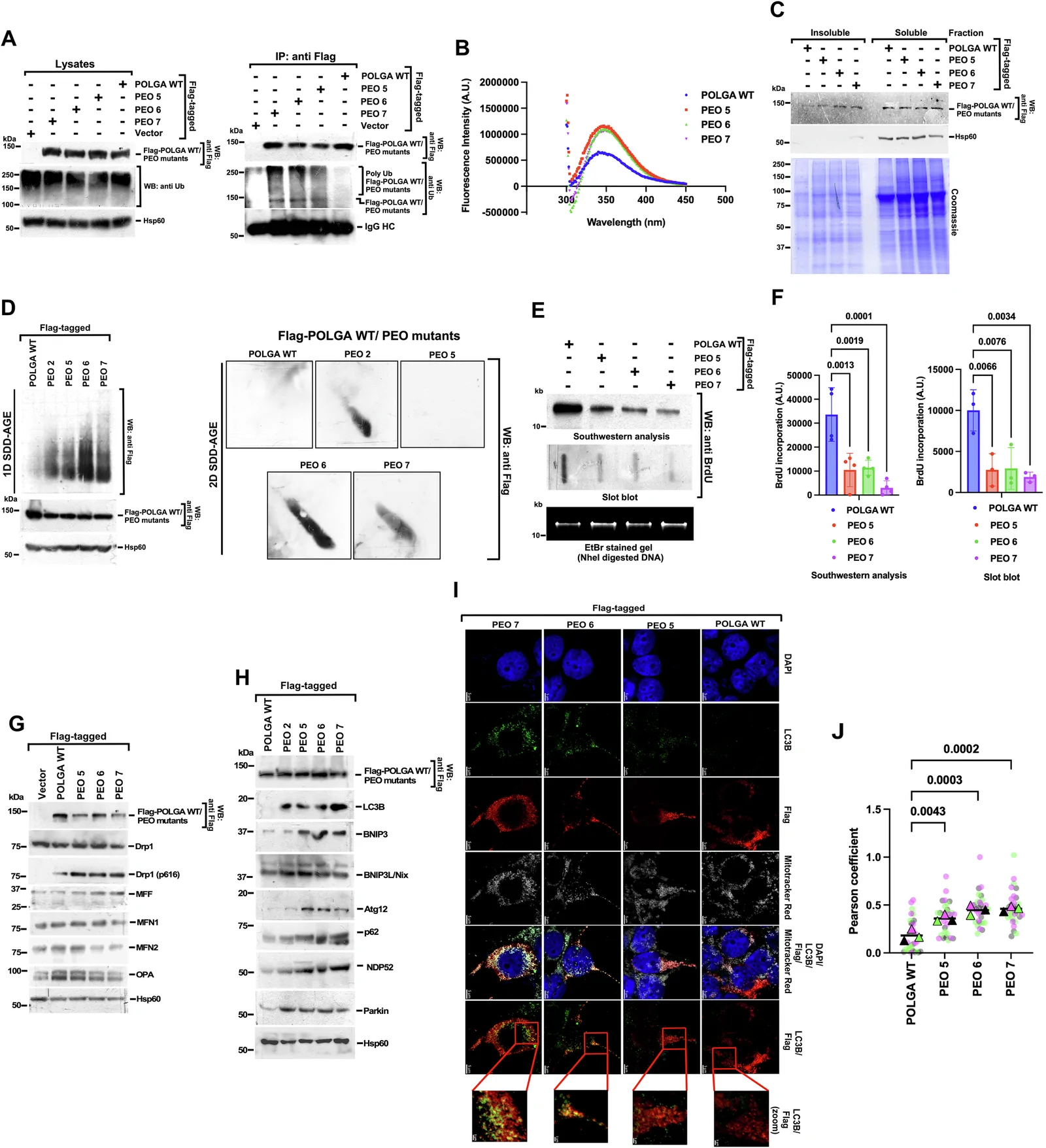
Hyperubiquitylated PEO mutants form aggregates that lead to mitophagy. (**A**) PEO mutants are hyperubiquitylated in cellular conditions. (Left) Western blot analysis was carried out with HEK293T cell extracts transiently expressing Flag-tagged POLGA WT, PEO 5, PEO 6 and PEO 7 with the indicated antibodies. (Right) Anti-Flag immunoprecipitation was carried out using anti-Flag beads on the same cell extracts. The extent of ubiquitylation was assessed by probing with anti-ubiquitin (P4D1) and anti-Flag antibodies. *n* = 3 biological replicates. (**B**) PEO mutants undergo conformational change. Recombinant His-tagged POLGA WT, PEO 5, PEO 6 and PEO 7 mutants were analysed using tryptophan fluorescence recorded from 300 to 450 nm. *n* = 3 biological replicates. (**C**) PEO mutants are enriched in the insoluble fraction. Soluble and insoluble fractions were separated from HEK293T cells transiently expressing Flag-tagged POLGA WT, PEO 5, PEO 6 and PEO 7 mutants. Immunoblotting was done with the indicated antibodies. (Bottom) Coomassie gel was used as a loading control. *n* = 3 biological replicates. (**D**) PEO 2, PEO 6 and PEO 7 form SDS-resistant aggregates. Cell lysates of HEK293T cells transiently expressing Flag-tagged POLGA WT, PEO 5, PEO 6 and PEO 7 were subjected to (Left) 1D SDD-AGE; (Right) 2D SDD-AGE. Immunoblots were probed with the indicated antibodies. n = 4 biological replicates. (**E**,** F**) Decreased mtDNA replication in PEO mutant-expressing cells. mtDNA was isolated from HEK293T cells transiently expressing Flag-tagged POLGA WT, PEO 5, PEO 6, PEO 7 post-treatment with BrdU. (**E**) mtDNA replication was analysed by either (Top) Southwestern blot or (Middle) Slot blot using an anti-BrdU antibody. (Bottom) EtBr-stained gel was used as a loading control. (**F**) Quantitation of (**E**). Mean ± SD. *n* = 3 biological replicates. (**G**) Mitochondrial fission is upregulated in PEO mutant-expressing cells. Cell lysates from HEK293T cells transiently expressing Flag-tagged POLGA WT, PEO 5, PEO 6 and PEO 7 were subjected to western blotting with the indicated antibodies. *n* = 3 biological replicates. (**H**–**J**) Mitophagy is upregulated in cells expressing PEO mutants. (**H**) Lysates made from HEK293T cells transiently expressing Flag-tagged POLGA WT, PEO 5, PEO 6 and PEO 7 were subjected to Western blot analysis with the indicated antibodies. *n* = 3 biological replicates. (**I**) Immunofluorescence followed by confocal microscopy was performed on HEK293T cells expressing either Flag-tagged POLGA WT, PEO 5, PEO 6 or PEO 7 with antibodies against LC3B and Flag. Mitochondria were stained with MitoTracker Red. The nucleus was stained with DAPI. Scale bar = 5 μm. (**J**) Quantitation of (**I**) using SuperPlots. Mean ± SD. *n* = 30 cells across 3 biological replicates. Data represent the mean ± SD and were analysed using one-way ANOVA (for **F**, **J**) statistical test with Dunnett’s multiple comparisons test.

We had earlier shown that two of the three tested PEO mutants, namely PEO 1 (F961S) and PEO 2 (A467T), were hyperubiquitylated by MITOL and accumulated in the insoluble aggregates-enriched fraction, which prevented their entry into mitochondria (Hussain et al, 2021). To investigate whether such a phenomenon was ubiquitous among the PEO mutants, three more PEO mutants, PEO 5 (T251I), PEO 6 (L304R), and PEO 7 (R943H) (Table EV1) were investigated. The tagged versions of the different PEO mutants were generated, purified (Fig. EV1A) and tested in different biochemical, biophysical and cellular assays. We found that PEO 6 showed minimal exonuclease activity (Fig. EV1B,C), while both PEO 5 and PEO 6 showed compromised polymerase activity (Fig. EV1D,E). All three tested PEO mutants were hyperubiquitylated in contrast to wild-type (WT) POLGA (Fig. 1A). The enhanced hyperubiquitylation of the PEO mutants led to increased turnover via proteasomal degradation (Fig. EV1F). In vitro import assay using Flag-tagged POLGA WT and PEO mutant proteins (Fig. EV1A) showed decreased entry into the mitochondrial matrix for PEO 6 and PEO 7 (Fig. EV1G), thereby identifying MITOL-dependent hyperubiquitylation as a key mechanism controlling the entry of these PEO mutants into mitochondria.

However, the question remained whether hyperubiquitylation of PEO mutants led to their aggregation. Enhanced tryptophan fluorescence was observed for the tested PEO mutants compared to POLGA WT (Fig. 1B), indicating their altered conformation. PEO 6 and PEO 7 showed enrichment in the insoluble fraction (Fig. 1C). Both 1D and 2D semi-denaturing detergent agarose gel electrophoresis (SDD-AGE) revealed that PEO 2, PEO 6 and PEO 7 formed stable aggregates, PEO 5 possibly formed dissociating unstable aggregates, while POLGA WT did not form any aggregates (Fig. 1D). Further dynamic light scattering (DLS) with purified Flag-tagged POLGA WT and PEO mutants showed increased hydrodynamic radii of the PEO mutants compared to POLGA WT (Fig. EV1H), suggesting that hyperubiquitylated PEO 6 and PEO 7 mutants undergo conformational changes and form aggregate-like structures, which hampered their mitochondrial import.

**Figure EV2:**
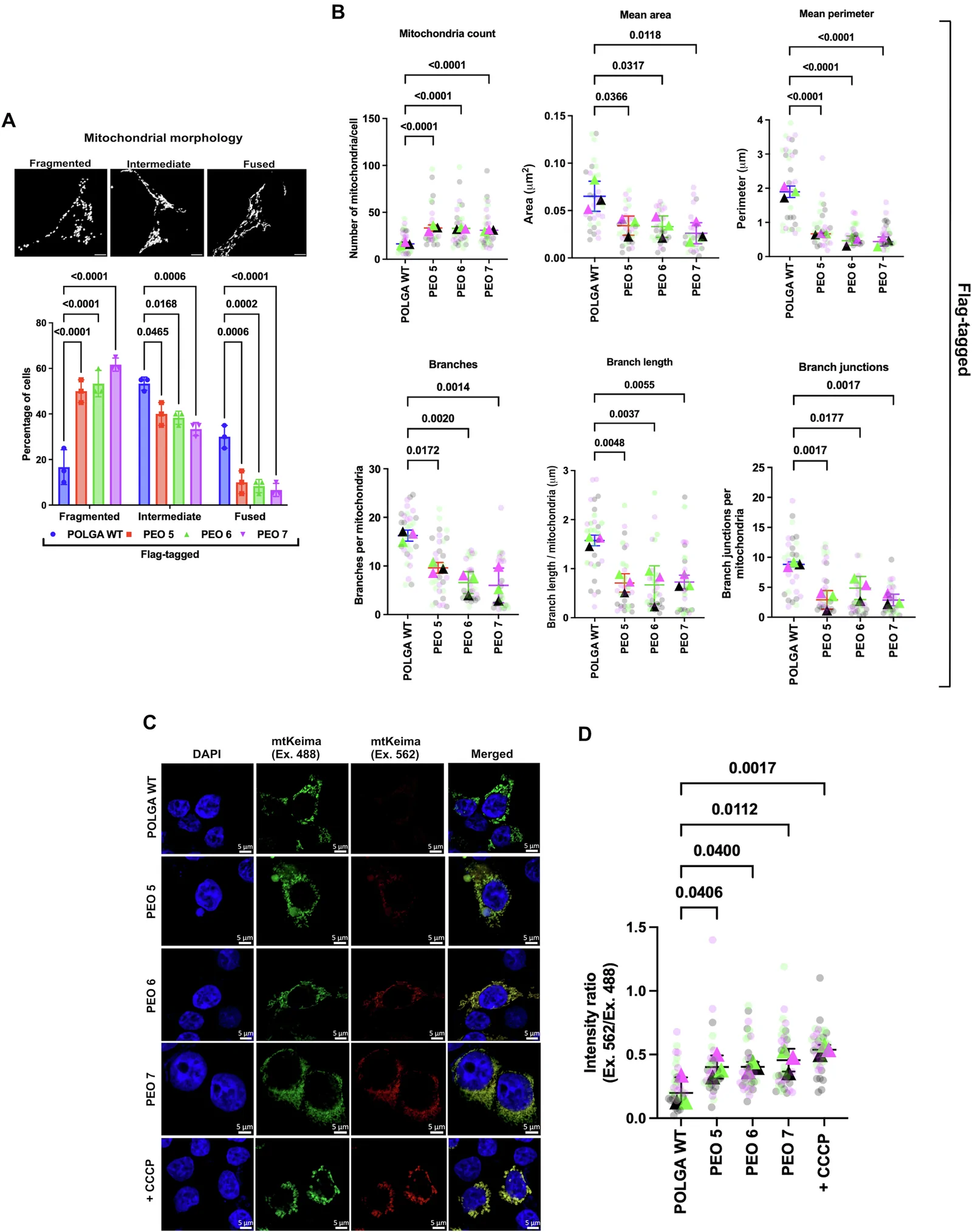
PEO mutant-expressing cells show mitochondrial fission and mitophagy. (**A**,** B**) Mitochondrial morphology is altered in cells expressing PEO mutants. (**A**) Mitochondrial morphology of HEK293T cells expressing Flag-tagged POLGA WT, PEO 5, PEO 6 and PEO 7 was monitored after staining with Mitotracker CMX ROS using immunofluorescence. (Top) Representative images are shown for fragmented, intermediate and fused mitochondria. (Bottom) Quantitation of fragmented, intermediate and fused mitochondria. (**B**) Parameters indicating mitochondrial fission are increased in cells expressing PEO mutants. Quantitation using SuperPlots was carried out using FIJI software, and the different mitochondrial morphology parameters were calculated using the mitochondrial analyser plugin (FIJI). *n* = 30 cells over three biological replicates. (**C**,** D**) Mitophagy was upregulated in PEO-expressing cells. (**C**) Mitophagy was monitored in HEK293T cells expressing POLGA WT, PEO 5, PEO 6 and PEO 7 using the mtKeima assay. CCCP treatment on the same cells was used as a positive control. The immunofluorescence-based readout was monitored. Scale bar = 5 μm. (**D**) Quantitation of (**C**) using SuperPlots. *n* = 35 cells over three biological replicates. Data represent the mean ± SD and were analysed using ordinary one-way (for **B**, **D**) and two-way ANOVA (for **A**) statistical tests with Tukey’s (**A**) and Dunnett’s (**B**, **D**) multiple comparisons tests.

Next, we wanted to understand the impact of decreased mitochondrial entry of PEO mutants on mitochondrial homoeostasis. As observed by both Southwestern and Slot blot analysis, cells expressing PEO mutants showed decreased BrdU incorporation compared to POLGA WT (Fig. 1E,F). Further, in cells expressing PEO mutants, elevated levels of mitochondrial fission markers [like MFF and Drp1 (p616)] were observed (Fig. 1G), along with increased fragmented mitochondria and decreased hyperfused mitochondria (Fig. EV2A), which resulted in decreased mitochondrial area, perimeter, branch length, and number of branches compared to POLGA WT (Fig. EV2B). Further enhanced expression of mitophagy markers (LC3B, NDP52, BNIP3, Parkin and p62) were observed in cells expressing PEO 5, PEO 6 and PEO 7 compared to POLGA WT (Fig. 1H). These PEO mutants with elevated LC3B levels showed increased colocalization with Flag-tagged PEO mutants (Fig. 1I, J). Use of the mtKeima fluorophore (Um et al, 2018) also showed enhanced mitophagy (Fig. EV2C,D), indicating deregulated mitochondrial replication and homoeostasis in cells expressing PEO mutants.

### Treatment with C4 disrupts PEO aggregates and enhances their entry into the mitoplast

**Figure EV3:**
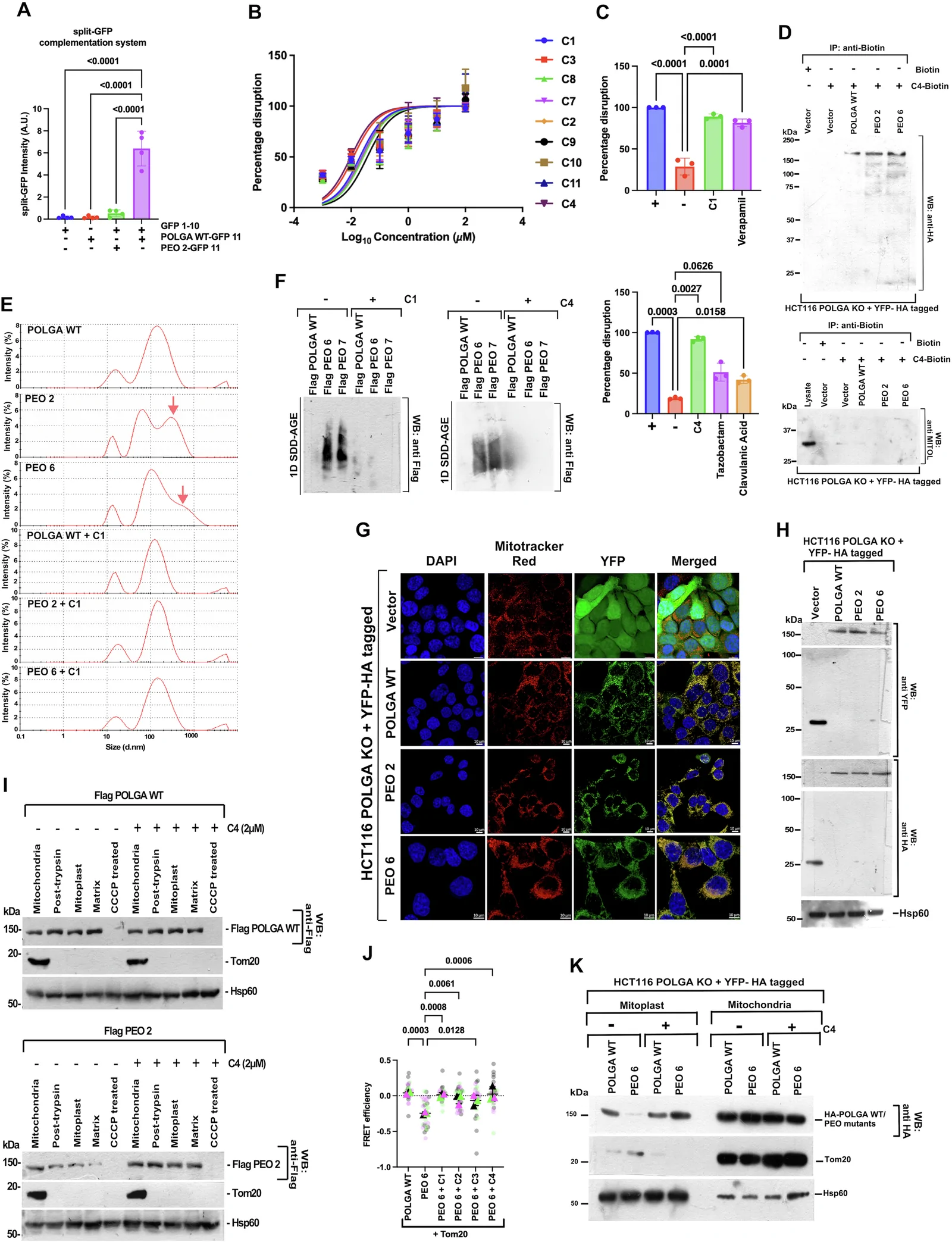
Treatment of cells expressing PEO mutants with small molecules leads to loss of aggregate formation and restores mitochondrial import. (**A**) Validation of the split-GFP complementation system for POLGA. The different split-GFP complementation pairs were expressed in HEK293T cells, and GFP fluorescence was measured and plotted as GFP fluorescence intensity. AU arbitrary units. Mean ± SD. *n* = 4 biological replicates. (**B**) Dose-response curve of small molecules that disrupt PEO 2 aggregates. A GFP-based protein complementation assay was carried out in the presence of a range of concentrations of the nine small molecules identified in the protein complementation assay. The concentrations used were 1 nM, 10 nM, 100 nM, 1 μM, 10 μM and 100 μM. *n* = 3 biological replicates. (**C**) Effect of C1 and C4 analogues on the interaction between PEO 2-GFP11 aggregates. Protein complementation assays were carried out with (Top) C1 and its analogue (Verapamil), (Bottom) C4 and its analogues (tazobactam and clavulanic acid). (+) indicates the positive control in which POLGA-GFP11 and GFP (1–10) were co-expressed. (−) indicates the negative control in which PEO 2-GFP11, along with GFP (1–10), was expressed. *n* = 3 biological replicates. (**D**) C4 interacts with POLGA WT and PEO mutants. Immunoprecipitation was performed using streptavidin beads to pull down biotin-labelled C4 compound incubated with the cell extracts of YFP-HA-tagged stable cell lines. The extent of interaction of (Top) POLGA/PEO and (Bottom) MITOL with biotinylated C4 was assessed by probing with the indicated antibodies. *n* = 3 biological replicates. (**E**) C1 treatment decreases aggregate formation by PEO mutants. Aggregation of POLGA WT, PEO 2 and PEO 6 was monitored by DLS (in ±C1 conditions) and represented as an intensity plot. The intensity plots of the proteins without C1 treatment are the same as those in Fig. 2E. *n* = 3 biological replicates. (**F**) Reversion of aggregation occurs after treatment with C1 and C4. Cell lysates were made from HEK293T cells transiently expressing Flag-tagged POLGA WT, PEO 6 and PEO 7 mutants, with and without C1 (Left) and C4 (Right) treatment. 1D SDD-AGE followed by immunoblotting was carried out with an anti-Flag antibody. n = 3 biological replicates. (**G**,** H**) YFP-HA-tagged stable cell line validation. HCT116 YFP-HA (termed vector), HCT116 POLGA WT-YFP-HA (termed POLGA WT), HCT116 (A467T)-YFP-HA (termed PEO 2) and HCT116 (L304R)-YFP-HA (termed PEO 6) stable cell lines were generated in HCT116 POLGA CRISPR knockout cells. The stable lines were validated by (**G**) immunofluorescence. Scale bar = 10 μm. (**H**) Western blotting using the indicated antibodies. *n* = 3 biological replicates. (**I**) PEO mutants show increased mitochondrial import after C4 treatment. In vitro import assay was carried out using in vitro transcribed and translated Flag-tagged POLGA WT (Top) and PEO 2 (Bottom) mutants, without and after treatment with C4. Post-incubation, each of the indicated mitochondrial fractions was subjected to Western blot analysis with the indicated antibodies. *n* = 3 biological replicates. (**J**) Identified small molecules enhance the interaction between PEO mutants and Tom20. Graphical representation, using SuperPlots, of FRET efficiency calculated based on the fluorescence intensity of the donor fluorophore for interaction between Tom20 and PEO 6 mutant, without or after treatment with C1, C2, C3 and C4 compounds. POLGA WT has been used as a positive control. *n* = 30 across three biological replicates. (**K**) C4 treatment restores the mitochondrial entry of PEO 6. Mitochondria and mitoplast fractions were isolated from HCT116 cells stably expressing YFP-HA-tagged POLGA WT and PEO 6 protein, grown without or after C4 treatment. (**J**) The relative presence of proteins in the mitoplast and mitochondrial fractions was determined by Western blot analysis using the indicated antibodies. *n* = 3 biological replicates. Data represent the mean ± SD and were analysed using an ordinary one-way (for **A**, **C** and **J**) statistical test with Sidak’s (**A**) and Dunnett’s (**C**, **J**) multiple comparisons test.

**Figure 2:**
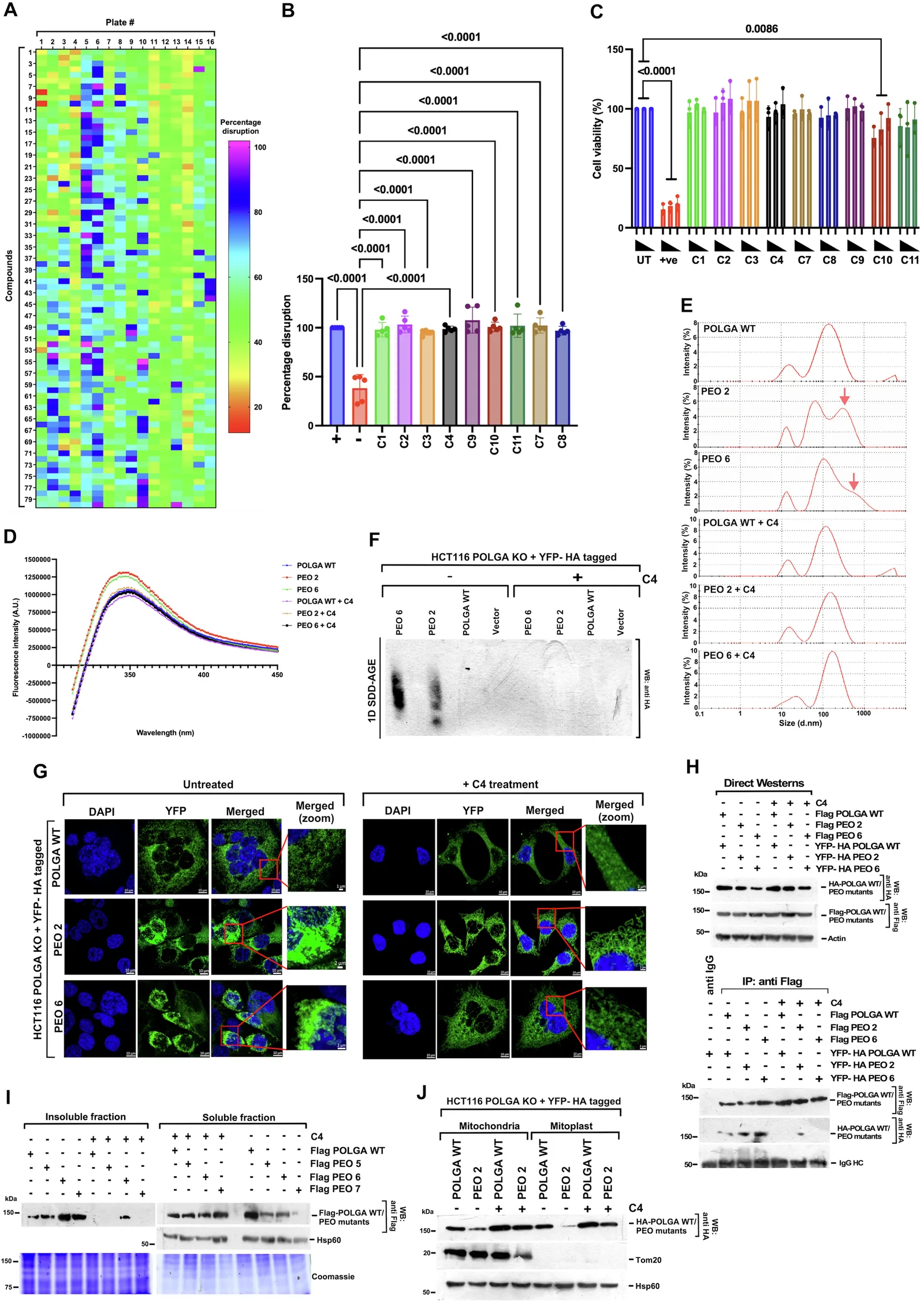
Small molecules that disrupt PEO aggregates allow their import into the mitoplast. (**A**) Heatmap showing the extent of PEO mutant disruption by the small molecules. A GFP-based protein complementation assay was carried out in the presence of the small molecules (100 μM) present in the Prestwick library by co-expressing PEO 2-GFP11 along with GFP (1–10) in HEK293T cells. The percentage of disruption was calculated relative to PEO 2-GFP11 + GFP (1–10) and was plotted as a heatmap. The screening of the entire library was done twice, and each time there were two technical duplicates. (**B**) Identification of nine small molecules that disrupt the interaction between PEO 2-GFP11 aggregates. Protein complementation assays were carried out with the nine small molecules, which had disrupted aggregation by more than 95% in (**A**). (+) indicates the positive control in which POLGA-GFP11, along with GFP (1–10), were co-expressed. (−) indicates the negative control, where PEO 2-GFP11, along with GFP (1–10) were co-expressed. *n* = 5 biological replicates. (**C**) Determining the cytotoxic impact of the identified small molecules. The cytotoxic impact was measured using the MTT assay across a concentration gradient (1 nM, 1 μM, 100 μM) of each compound. (+ve) indicates the positive control in which cells are treated with camptothecin for 16 h. *n* = 3 biological replicates. (**D**) Change in the conformation of PEO mutants occurs upon C4 treatment. Tryptophan fluorescence of POLGA WT, PEO 2 and PEO 6 proteins was recorded upon treatment without or with C4 treatment. Spectra were recorded from 300 to 450 nm. *n* = 3 biological replicates. (**E**) C4 treatment disrupts high-order aggregates in PEO mutants. POLGA WT, PEO 2 and PEO 6 proteins were monitored by DLS and represented as size-by-intensity plots. *n* = 3 biological replicates. (**F**,** G**) Small molecule reverses PEO mutant aggregation in stable cell lines. (**F**) Cell lysates of HCT116 POLGA KO cells stably expressing YFP-HA-tagged POLGA WT, PEO 2 and PEO 6 proteins were grown in the absence or presence of C4 treatment. Lysates were analysed by 1D SDD-AGE using an anti-HA antibody. *n* = 4 biological replicates. (**G**) Immunofluorescence followed by confocal microscopy was performed in HCT116 cells stably expressing YFP-HA-tagged POLGA WT, PEO 2, PEO 6, and grown without or with C4 treatment. The nucleus was stained with DAPI. Scale bar = 10 μm and 2 μm (for the zoomed image). (**H**) C4 treatment disrupts the interaction between PEO multimers in cells. Flag-tagged POLGA WT, PEO 2 and PEO 6 were transiently expressed in cell lines stably expressing YFP-HA-tagged POLGA WT, PEO 2 and PEO 6. Cells were either left untreated or treated with C4. (**H**, top) Western analysis of cell lysates was carried out with the indicated antibodies. (**H**, bottom) Anti-Flag immunoprecipitation was performed on these cell lysates, and immunoblotting was carried out with the indicated antibodies. *n* = 3 biological replicates. (**I**) PEO mutants accumulate in soluble fractions after C4 treatment. Soluble and insoluble fractions were separated from HEK293T cells expressing Flag-tagged POLGA WT, PEO 5, PEO 6 and PEO 7 mutants with and without treatment with C4. The presence of the POLGA variants in the two fractions was determined by Western analysis using the indicated antibodies. Coomassie gel shows equal protein loading in all conditions. *n* = 3 biological replicates. (**J**) C4 treatment restores PEO mutant mitochondrial entry. Mitochondria and mitoplast fractions were isolated from HCT116 cells stably expressing YFP-HA-tagged POLGA WT and PEO 2, grown without or with C4 treatment. The presence of the proteins in the mitoplast and mitochondrial fractions was determined by Western analysis using the indicated antibodies. *n* = 3 biological replicates. Data were represented as mean ± SD and analysed using RM one-way ANOVA (for **B**) and Ordinary two-way ANOVA (for **C**) statistical tests with Dunnett’s multiple comparisons test.

Next, we wanted to test whether the disruption of the PEO aggregates allowed the PEO mutants to enter the mitochondria and restore mitochondrial functions. Hence, we carried out a split-GFP-based complementation assay (Chun et al, 2007) with POLGA WT and PEO 2, with the latter known to form aggregate-like structures (Hussain et al, 2021). While POLGA WT-GFP11 complemented with GFP1–10, PEO 2-GFP11 did not (Fig. EV3A). Next, we screened the FDA/EMA/PMDA-approved small molecule Prestwick library using the split-GFP complementation assay (Fig. 2A). Nine small molecules were initially identified that disrupted PEO 2 aggregates by >95% (Fig. 2B) in the low nanomolar range, as observed from their EC_50_ value (Fig. EV3B; Table EV2). These nine small molecules did not show any cellular toxicity (Fig. 2C). Out of the above nine small molecules, two small molecules (C1 and C4) with the lowest EC_50_ values (Table EV2) and having minimal possible effect on mitochondrial homoeostasis were chosen for most of the subsequent assays. To determine the specificity of C1 and C4, analogues of C1 and C4 (from the same pharmacological class) were tested. In the split GFP-based complementation assay, verapamil (C1 analogue) showed similar PEO aggregate disruption efficiency as the C1 compound, whereas Tazobactam and Clavulanic acid (C4 analogues) showed decreased efficiency in disrupting PEO aggregates compared to C4 (Fig. EV3C).

To determine whether small molecules directly interact with POLGA and/or with MITOL, C4 was labelled with Biotin using click chemistry. Anti-Biotin pulldown showed that the interaction of C4 with PEO 2 and PEO 6 was enhanced compared to POLGA WT (Fig. EV3D, top). However, C4 did not interact with MITOL (Fig. EV3D, bottom). C4 treatment reverted the tryptophan fluorescence of PEO mutants to levels comparable to POLGA WT, indicating a change in the 3D conformation of PEO mutants post-treatment (Fig. 2D). DLS analysis of GST-tagged POLGA WT, PEO 6, and PEO 7 following C4 (Fig. 2E) and C1 (Fig. EV3E) treatment showed that the hydrodynamic radii of the PEO mutants post-treatment were decreased to POLGA WT levels. Further SDD-AGE indicated disruption of the HA- or Flag-tagged PEO mutant aggregates upon C4 (Figs. 2F and EV3F, right) or C1 (Fig. EV3F, left) treatment.

The ability of C1 and C4 to disrupt PEO 2/PEO 6 was further validated in isogenic stable lines created in HCT116 sgRNA POLGA cells (Somuncu et al, 2022). These cells were stably complemented with either YFP-HA-tagged POLGA WT or PEO 2 or PEO 6 and subsequently validated by immunofluorescence (Fig. EV3G) and western blot analysis (Fig. EV3H). Immunofluorescence and confocal microscopy indicated that the reconstituted PEO 2 and PEO 6 lines showed significantly increased aggregation in untreated conditions, which was reduced when cells were grown in the presence of C4 (Fig. 2G). The disruption of the aggregates by C4 was also observed using immunoprecipitation experiments, when Flag-tagged counterparts of POLGA/PEO were transiently expressed in the reconstituted YFP-HA lines (Fig. 2H). Cellular fractionation indicated decreased enrichment of PEO mutants in the insoluble fraction and concomitantly their increased presence in the soluble fraction after C4 treatment (Fig. 2I).

To assess the ability of the PEO mutants to enter mitochondria upon small molecule treatment, in vitro import assays were carried out using in vitro transcribed and translated Flag-tagged POLGA WT and PEO mutant proteins. Enhanced entry of PEO 2 into the mitochondrial matrix was observed following C4 treatment (Fig. EV3I, bottom), comparable to that observed for POLGA WT (Fig. EV3I, top). To quantitatively assess the ability of the PEO mutants to enter mitochondria upon small molecule treatment, a FRET-based interaction assay between YFP-tagged Tom20 and CFP-tagged POLGA WT/PEO 6 was carried out. Using the acceptor photobleaching method, decreased FRET efficiency values were observed for the PEO 6-Tom20 interaction, which was reversed upon treatment with small molecules (Fig. EV3J). Further validation came from mitochondrial fractionation, where treatment with C4 increased the enrichment of YFP-HA-tagged PEO 2 (Fig. 2J) and PEO 6 (Fig. EV3K) mutants into the mitoplast fraction.

### Treatment with C4 reverts hyperubiquitylation of PEO mutants and restores mtDNA replication

**Figure EV4:**
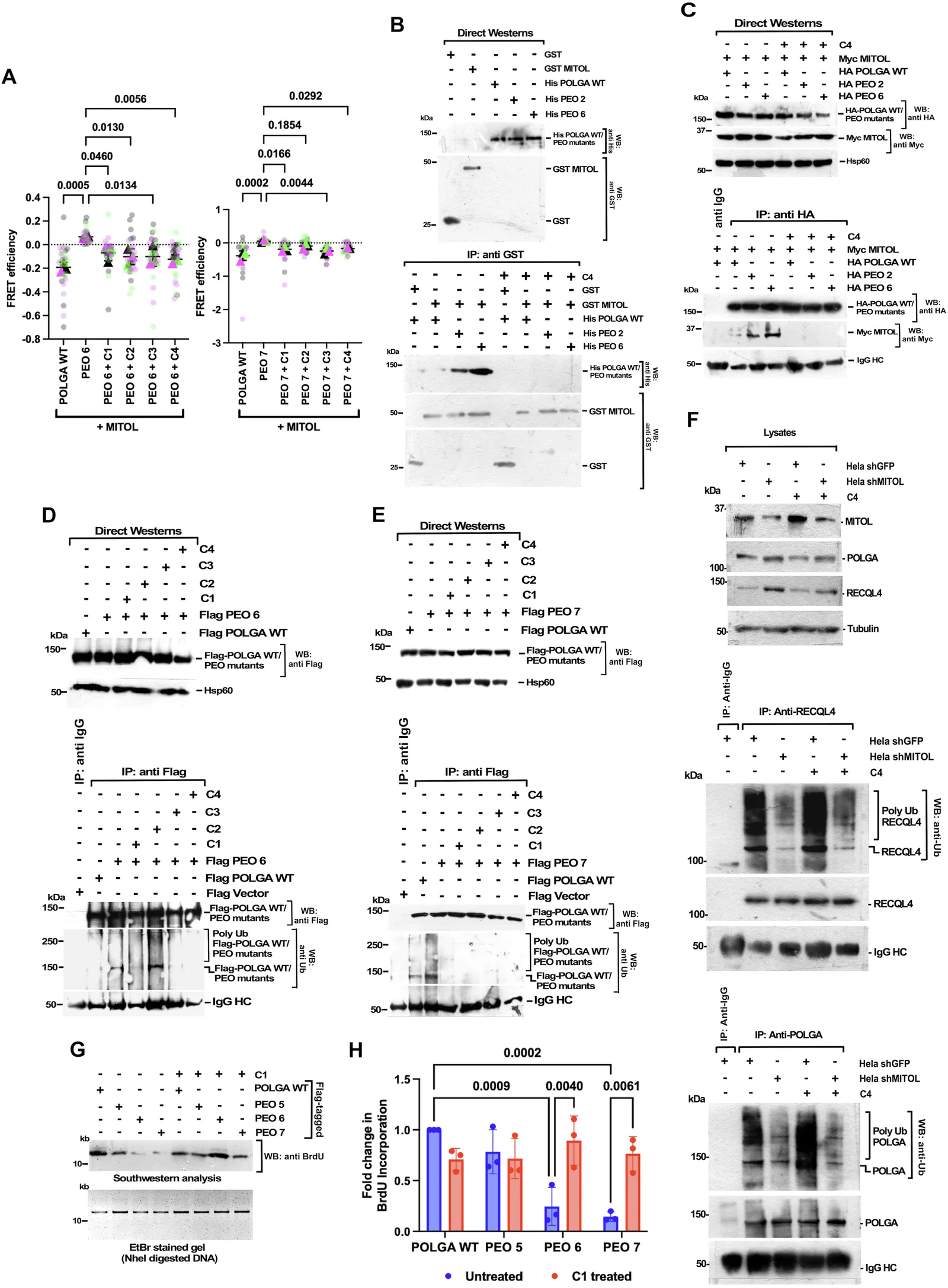
Small molecule treatment disrupts the interaction of PEO mutants with MITOL. (**A**) Identified small molecules disrupt the interaction between PEO mutants and MITOL. Graphical representation, using SuperPlots, of FRET efficiency calculated based on the fluorescence intensity of the donor fluorophore for interaction between (Left) MITOL and PEO 6 mutant or (Right) MITOL and PEO 7 mutant, without or after treatment with C1, C2, C3 and C4 compounds (*n* = 30 cells over three biological replicates). POLGA WT/MITOL FRET were used as the control. (**B**) Small molecules disrupt the interaction between GST MITOL and His-PEO mutants in vitro. In vitro interaction assays were carried out with recombinant soluble His-tagged POLGA WT, PEO 2, PEO 6 and bound GST-tagged MITOL in the absence or presence of C4. (**B**, top) Input of the recombinant proteins. (**B**, bottom) Post-interaction. For both input and post-interaction blots, Western analysis was carried out with the indicated antibodies. Mean ± SD. *n* = 4 biological replicates. (**C**) Small molecules disrupt the interaction between MITOL and PEO mutants in cells. Myc-tagged MITOL was transiently expressed in stable cell lines expressing YFP-HA-tagged POLGA WT or PEO 2 or PEO 6. Cells were either left untreated or treated with the C4 compound. (**D**, Top) The expression of the proteins was checked with the indicated antibodies. (**D**, Bottom) Anti-HA immunoprecipitation was performed on these cell lysates, and immunoblots were carried out with the indicated antibodies. *n* = 4 biological replicates. (**D**,** E**) Small molecules decrease the extent of ubiquitylation on PEO mutants. (Top of both **D**, **E**) Western blotting analysis was performed with the cell extracts of HEK293T cells transiently expressing Flag-tagged POLGA WT or (**D**) PEO 6 or (**E**) PEO 7 after treatment with C1, C2, C3, and C4. Western analysis was done with the indicated antibodies. (Bottom of both **D**, **E**) Immunoprecipitation was performed using anti-Flag beads with the same cell extracts. The extent of POLGA/PEO ubiquitylation was assessed by probing with the indicated antibodies. *n* = 4 biological replicates. (**F**) C4 treatment does not alter MITOL-mediated ubiquitylation of POLGA and RECQL4. (Top) Cell extracts were made from HeLa shGFP and HeLa shMITOL cells grown either without or with C4 treatment. Western blots were carried out with the indicated antibodies. Immunoprecipitation was carried out on the same cell extracts with (Middle) anti-RECQL4 and (Bottom) anti-POLGA antibodies. The extent of ubiquitylation was assessed by probing with anti-ubiquitin (P4D1) and anti-POLGA/RECQL4 antibodies. n = 3 biological replicates. (**G**,** H**) C1 treatment restores mtDNA replication in cells expressing PEO mutants. (**G**) mtDNA was isolated from BrdU-treated HEK293T cells expressing Flag-tagged wild-type POLGA, PEO 5, PEO 6, and PEO 7, which have been grown either with or without C1. mtDNA replication was analysed by measuring the extent of BrdU incorporation using Southwestern blot analysis with an anti-BrdU antibody. EtBr gel was used as a loading control. (**H**) Quantitation of (**G**). *n* = 3 biological replicates. Data represent the mean ± SD and were analysed using ordinary one-way (for **A**) and two-way ANOVA (for **H**) statistical tests with Dunnett’s (**A**) and Tukey’s (**H**) multiple comparisons tests.

We have earlier reported that hyperubiquitylated PEO mutants interact better with MITOL compared to POLGA WT and thereby undergo hyperubiquitylation (Hussain et al, 2021). FRET-based analysis using CFP-tagged PEO 6 and PEO 7 and YFP-tagged MITOL now indicates the reversal of enhanced PEO-MITOL interaction upon treatment with small molecules (Fig. EV4A). This effect was recapitulated using both in vitro (Fig. EV4B) and in cells (Fig. EV4C) interaction assays between MITOL and POLGA WT/PEO mutants. Further, treatment with C4 decreased the hyperubiquitylation status of PEO 6 (Fig. EV4D), whereas both C1 and C4 decreased the hyperubiquitylation status of PEO 7 (Fig. EV4E) compared to untreated conditions.

To further determine whether C4 influences the E3 ligase function of MITOL by modulating the ubiquitylation of another MITOL target like RECQL4 (Hussain et al, 2023), ubiquitylation assays were conducted in HeLa shGFP and HeLa shMITOL cells following C4 treatment. C4 treatment did not alter the ubiquitylation patterns of either RECQL4 or POLGA in HeLa shGFP cells, suggesting that C4 treatment neither affects MITOL E3 ligase activity nor influences the ubiquitylation status of its substrates (Fig. EV4F).

**Figure 3:**
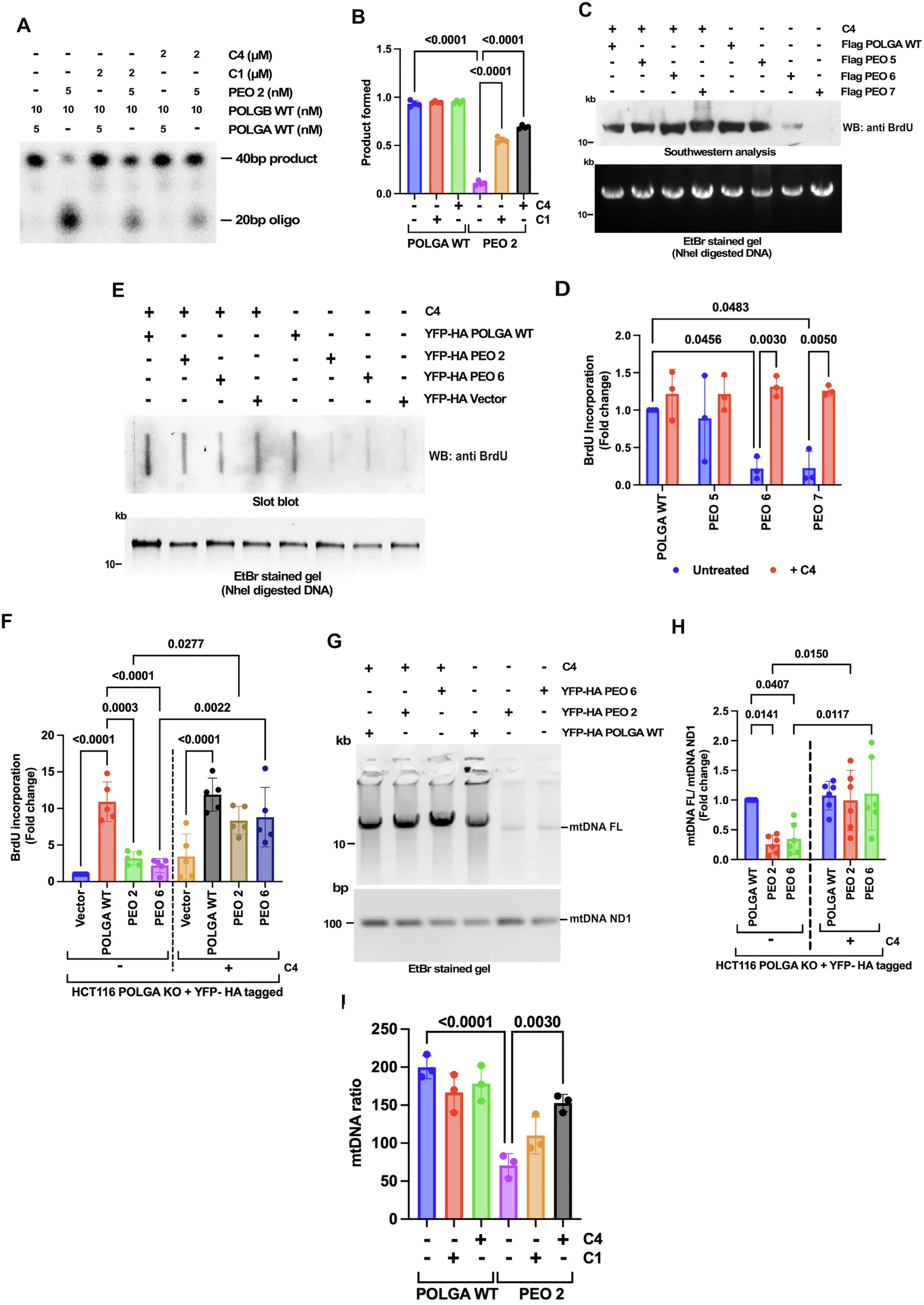
C4 treatment restored mtDNA replication and integrity in PEO mutant-expressing cells. (**A**,** B**) C1 and C4 treatments restored in vitro polymerase activity of PEO 2. (**A**) In vitro primer extension assay was performed with GST-tagged POLGA WT or PEO 2, using a radiolabelled annealed substrate. The reaction was performed with the indicated concentration of PEO 2 and POLGA WT, without or in the presence of C1 or C4. (**B**) Quantitation of (**A**). *n* = 4 biological replicates. (**C**–**F**) mtDNA replication in cells expressing PEO mutants is restored following C4 treatment. mtDNA was isolated from BrdU-treated (**C**) HEK293T cells transiently expressing Flag-tagged POLGA WT, PEO 5, PEO 6, PEO 7 or from (**E**) HCT116 cells stably expressing POLGA WT, PEO 2, PEO 6. In both cases, cells were either grown without or in the presence of C4. mtDNA replication was analysed by measuring the extent of BrdU incorporation using (**C**) Southwestern blot and (**E**) Slot blot analysis. Immunoblots were carried out with anti-BrdU antibody. EtBr gel for mtDNA serves as a loading control. (**D**, **F**) Quantitation of (**C**, **E**). *n* = 3 biological replicates for (**C**) and *n* = 5 biological replicates for (**E**). (**G**, **H**) mtDNA integrity in cells expressing PEO mutants is restored upon C4 treatment. mtDNA was isolated from YFP-HA-tagged stable cell lines expressing either POLGA WT, PEO 2, or PEO 6 and grown either untreated or treated with C4. PCR reaction was carried out to amplify either (Top) the complete 16.6 kb mtDNA or (Bottom) a 100 bp long mtND1 amplicon (used as a control). In both cases, mtDNA was visualised by EtBr staining in agarose gels. (**H**) Quantitation of (**G**). *n* = 6 biological replicates. (**I**) mtDNA content in cells expressing the PEO 2 mutant is increased following C4 treatment. The mtDNA ratio was estimated from stable cells expressing POLGA WT or PEO 2, either untreated or treated with C1 or C4 compound. qPCR analysis was done using β-globin as the nuclear gene and cytochrome b as the mitochondrial gene, *n* = 3 biological replicates. Data represent the mean ± SD and were analysed using one-way ANOVA (for **B**, **F**, **I**) and ordinary two-way ANOVA (for **D**, **H**) statistical tests with Tukey’s multiple comparison test.

Next, we wanted to determine whether the restored mitochondrial entry impacts mtDNA replication and integrity. Using the in vitro primer extension assay, it was found that both C1 and C4 treatments enhanced the replication capability of the PEO 2 mutant (Fig. 3A,B). Both Southwestern (Figs. 3C,D and EV4G,H) and Slot blot (Fig. 3E,F) analysis further showed increased BrdU incorporation in the PEO mutant following C4 and C1 treatment, indicating restoration of mtDNA replication. In addition, the mtDNA integrity assay indicated that the replication of the entire 16.6 kb of mtDNA was restored for PEO 2 and PEO 6 expressing cells following C4 treatment (Fig. 3G,H). This culminates in an increase in the mtDNA ratio in the presence of either C1 or C4 compared to the untreated condition (Fig. 3I).

### C4 treatment restored mitochondrial homoeostasis and decreased mitophagy

**Figure 4:**
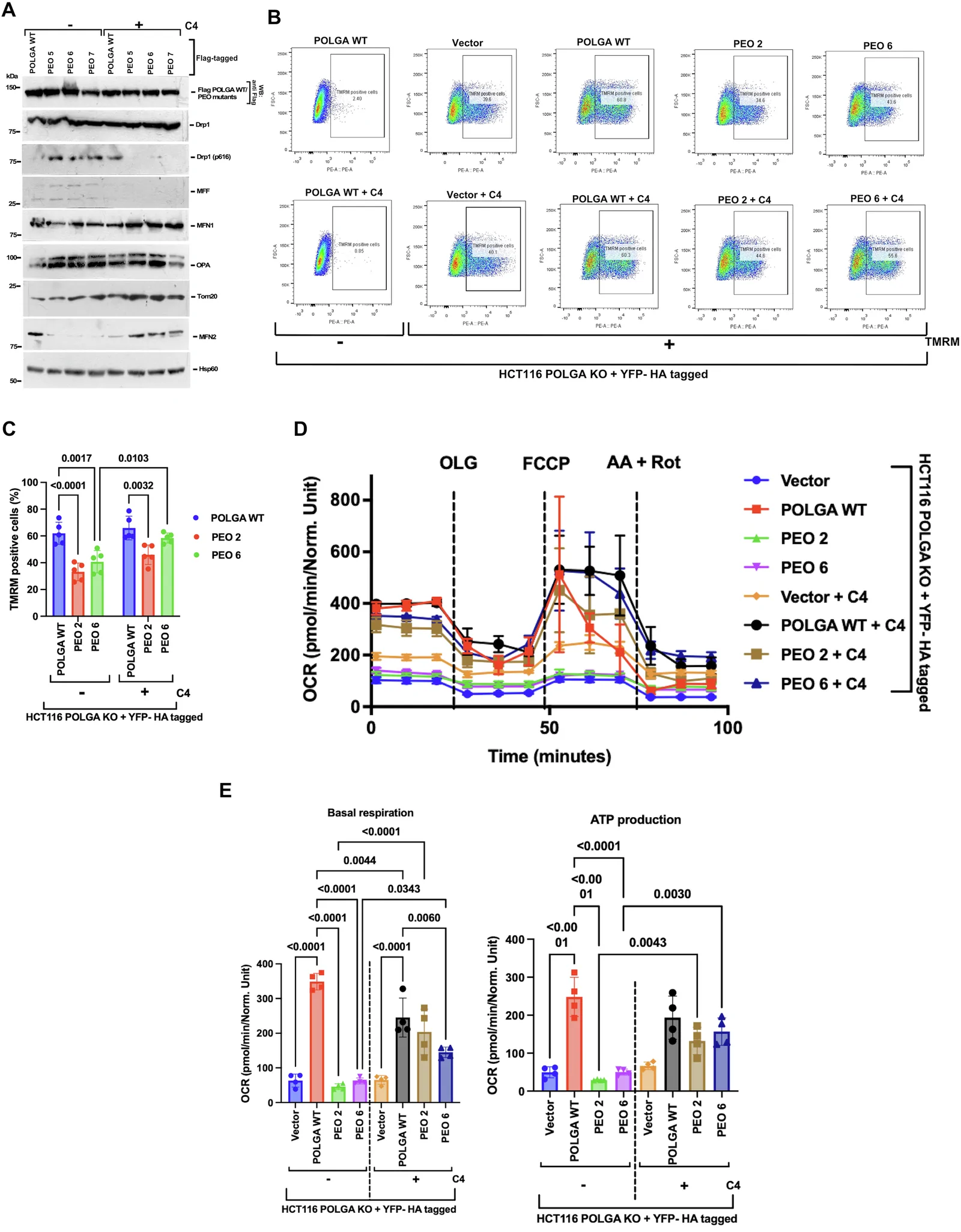
C4 treatment restored mitochondrial homoeostasis in PEO mutant-expressing cells. (**A**) Mitochondrial fusion is upregulated in cells expressing PEO mutants after C4 treatment. Cell lysates were made from HEK293T cells transiently expressing Flag-tagged POLGA WT, PEO 5, PEO 6 and PEO 7 mutants grown either without or in the presence of C4. Western blots were carried out using the indicated antibodies. *n* = 3 biological replicates. (**B**,** C**) Mitochondrial membrane potential is restored in cells expressing PEO mutants upon C4 treatment. (**B**) Mitochondrial potential was analysed using TMRM in stable lines expressing YFP-HA-tagged POLGA WT, PEO 2 and PEO 6 and grown either in the absence or presence of C4. The dot plot shows the percentage of TMRM-positive cells. (**C**) Quantitation of (**B**). *n* = 5 biological replicates. (**D**) Oxygen Consumption Rate in cells expressing PEO mutants is enhanced after C4 treatment. Oxygen consumption rate (OCR) was determined in stable lines expressing YFP-HA-tagged POLGA WT, PEO 2 and PEO 6 and grown in the absence or presence of C4 by carrying out a Seahorse mitostress test on live cells by sequentially injecting oligomycin (OLG), FCCP and antimycin A + rotenone  (AA + Rot). Mean ± SEM. *n* = 4 biological replicates. (**E**) Mitochondrial respiration is restored upon C4 treatment. Seahorse mitostress test was performed in stable lines expressing YFP-HA-tagged POLGA WT, PEO 2 and PEO 6 and grown in the absence or presence of C4 to estimate the rates of basal respiration and ATP production. *n* = 4 biological replicates. Data represent the mean ± SD and were analysed using one-way ANOVA (for **E**) and an ordinary two-way ANOVA (for **C**) statistical test with Tukey’s multiple comparison test.

Next, we wanted to determine whether the treatment with small molecules affected mitochondrial homoeostasis. C4 treatment led to decreased expression of mitochondrial fission markers [like MFF and Drp1(p616)] and concomitantly increased levels of the fusion markers (like MFN2) in cells transiently expressing PEO mutants (Fig. 4A). Both PEO 2 and PEO 6 mutants showed decreased mitochondrial membrane potential compared to POLGA WT in the untreated conditions (Fig. 4B, top). Following C4 treatment, mitochondrial membrane potential was significantly improved and almost reached the levels of POLGA WT (Fig. 4B,C).

**Figure 5:**
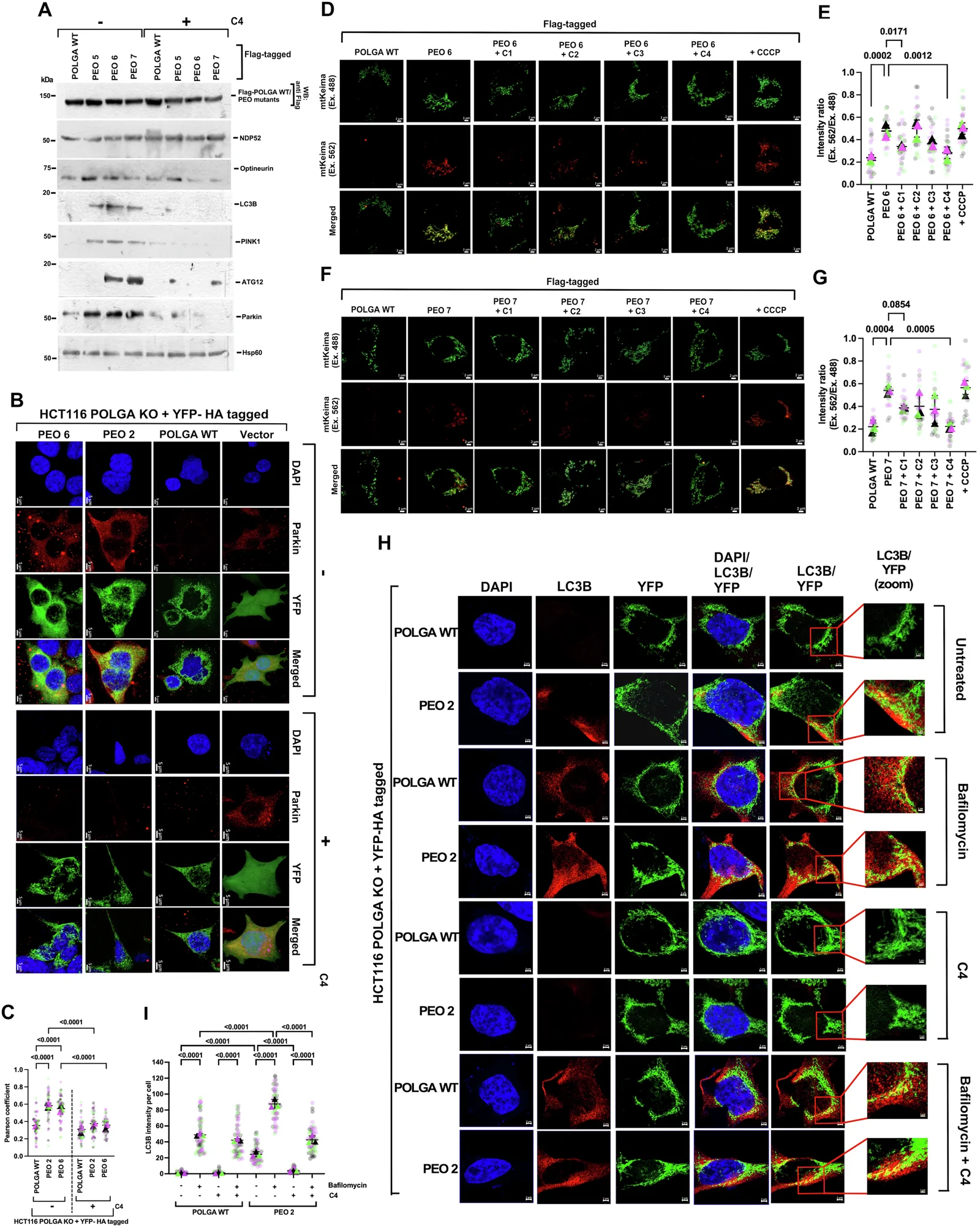
C4 treatment reduced mitophagy in PEO mutant-expressing cells. (**A**) Expression of mitophagy markers is decreased in cells expressing PEO mutants upon C4 treatment. Flag-tagged POLGA WT, PEO 5, PEO 6, and PEO 7 were transiently expressed in HEK293T cells, and cell lysates were prepared. Western blots were carried out with the indicated antibodies. n = 3 biological replicates. (**B**, **C**) Mitochondrial recruitment of Parkin is decreased in PEO-expressing cells post C4 treatment. Immunofluorescence followed by confocal microscopy was performed on stable lines expressing YFP-HA-tagged POLGA WT, PEO 2 and PEO 6 mutants grown without or upon C4 treatment. (**B**) Immunofluorescence was carried out with antibodies against Parkin. The nucleus was stained with DAPI. Scale bar = 5 μm. (**C**) Quantitation of (**B**) using SuperPlots. *n* = 60 cells across 3 independent biological replicates. (**D**–**G**) mtKeima assay shows decreased mitophagy in PEO mutants upon small molecule treatment. Mitophagy was monitored in HEK293T cells expressing (**D**) POLGA WT, PEO 6 and (**F**) POLGA WT, PEO 7 using the mtKeima assay. For this purpose, mtKeima was expressed without or in the presence of either C1, C2, C3 or C4. CCCP was used as a positive control. The immunofluorescence-based readout was monitored. Scale bar = 2 μm. (**E**, **G**) Quantitation of (**D**, **F**) using SuperPlots. *n* = 25 cells across 3 independent biological replicates. (**H**,** I**) PEO 2 mutant-expressing cells display enhanced autophagic flux upon bafilomycin treatment. Immunofluorescence followed by confocal microscopy was performed on stable cell lines expressing YFP-HA-tagged POLGA WT and PEO 2 mutant, grown asynchronously or upon C4 or bafilomycin treatment. (**H**) Immunofluorescence was carried out with antibodies against LC3B. The nucleus was stained with DAPI. Scale bar = 2 μm and 1 μm (for zoomed image). (**I**) Quantitation of (**H**) using SuperPlots. *n* = 60 cells across 3 independent biological replicates. Data represent the mean ± SD and were analysed using one-way ANOVA (for **C**, **E**, **G**, **I**) statistical test with Dunnett’s (for **E**, **G**) and Tukey’s (for **C**, **I**) multiple comparison test.

To further investigate the effect of small molecules on mitochondrial homoeostasis, we determined the status of the different mitochondrial respiration parameters in stable lines expressing YFP-HA-tagged POLGA WT, PEO 2 and PEO 6 in both untreated and C4-treated conditions using the Seahorse XFe24 system (Fig. 4D). The decreased basal respiration and ATP production in stable lines expressing PEO 2 and PEO 6 were reversed upon treatment with C4 (Fig. 4E). Furthermore, the improved mitochondrial homoeostasis parameters affected mitophagy in PEO mutants. Hence, post-C4 treatment, PEO mutant-expressing cells manifested decreased expression of the mitophagy markers (like LC3B, PINK1, ATG12 and Parkin) compared to the untreated condition, as determined by Western analysis (Fig. 5A) and immunofluorescence and confocal microscopy (Fig. 5B,C). To further validate the decreased mitophagy upon treatment with small molecules, we performed the live-cell imaging-based mtKeima mitophagy detection assay. The enhanced fluorescence observed in cells expressing PEO 6 and PEO 7 mutants upon excitation at 562 nm wavelength (acidic pH) in the untreated conditions was decreased upon treatment with C1 and C4 (Fig. 5D–G). LC3B expression was significantly elevated in PEO 2 mutant cells compared with the POLGA WT control. Upon bafilomycin treatment, a further accumulation of LC3B was observed, consistent with increased autophagic flux (Fig. 5H). Notably, co-treatment with C4 and bafilomycin resulted in a significant reduction in LC3B signals in PEO 2 (Fig. 5H,I), suggesting that C4-mediated restoration of mitochondrial homoeostasis prevents elevated mitophagy in PEO 2 mutant-expressing cells.

### Small-molecule treatment restored motor functions in PEO 2-expressing knock-in mice

**Figure 6:**
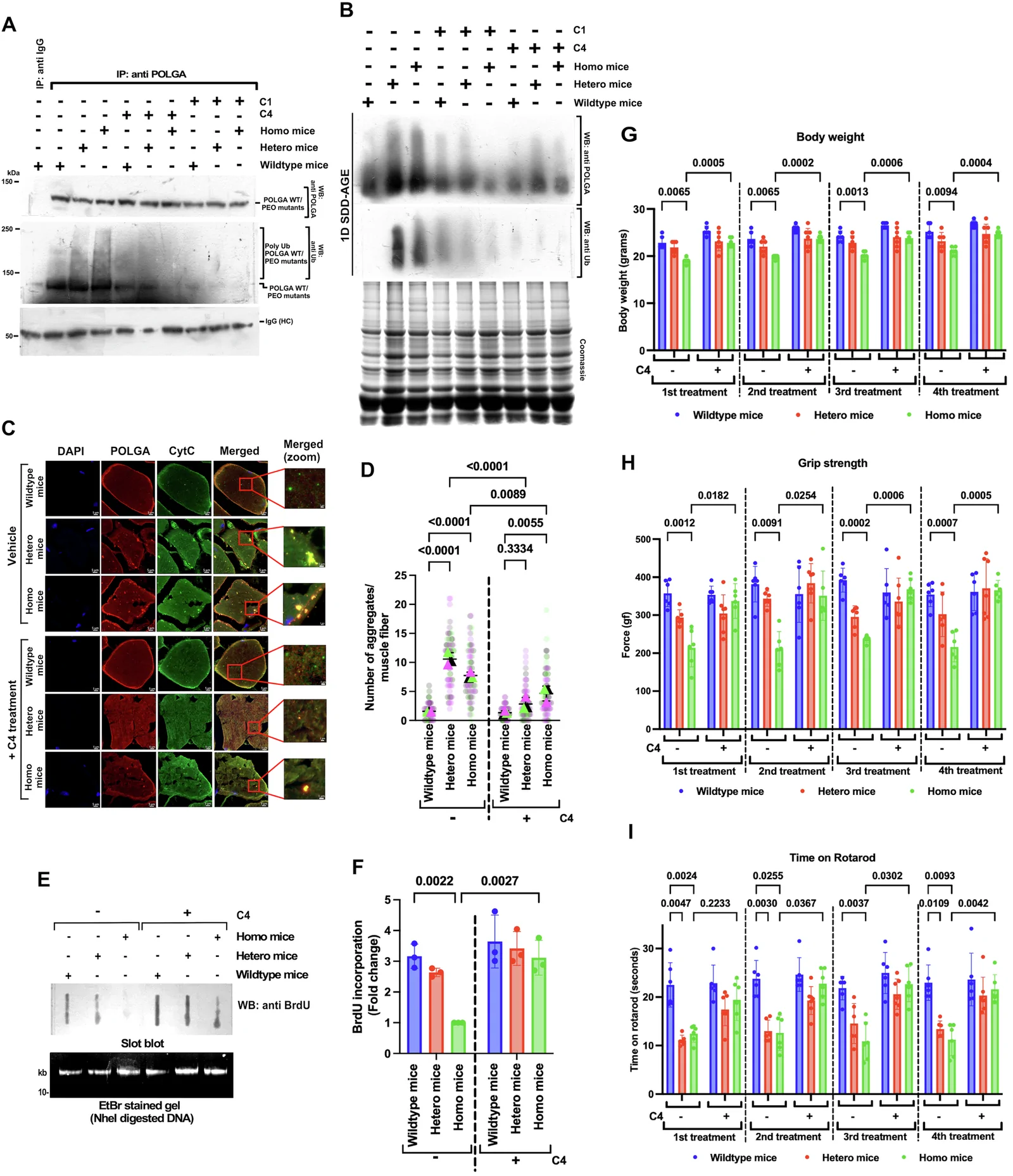
Mitochondrial homoeostasis and motor functions are restored in PEO mutant-expressing mice after C4 treatment. (**A**) Small molecules revert POLGA hyperubiquitylation in Hetero (POLG^WT/A449T^) and Homo (POLG^A449T/A449T^) mice. Skeletal muscle tissue extracts were prepared from POLGA WT, Hetero and Homo mice treated with either vehicle, C1 or C4. Immunoprecipitation with anti-POLGA antibody was performed with the extracts indicated above. The extent of ubiquitylation was assessed by probing with anti-ubiquitin (P4D1) antibody. *n* = 3 mice of each genotype. (**B**–**D**) C4 treatment disrupts POLGA aggregates in Hetero (POLG^WT/A449T^) and Homo (POLG^A449T/A449T^) mice skeletal muscles. (**B**) Skeletal muscle tissue extracts were prepared from POLGA WT, Hetero, and Homo mice treated with either vehicle, C1 or C4. The extracts were subjected to 1D SDD-AGE and analysed using antibodies against POLGA and ubiquitin. Coomassie-stained gel was used as a loading control. *n* = 3 mice of each genotype. (**C**) The extent of aggregation in the mice was analysed using immunofluorescence followed by confocal microscopy by staining the skeletal tissue sections with antibodies against POLGA and cytochrome c (CytC). Scale bar = 5 µm and 1 μm (for zoomed image). (**D**) Quantitation of (**C**) using SuperPlots. Mean ± SD. *n* = 80 muscle fibres from three biological replicates. (**E**,** F**) mtDNA replication is restored in the PEO mice model following C4 treatment. In vivo BrdU incorporation assay was performed after treatment with either the vehicle or C4 in 7-week-old POLGA WT, Hetero, Homo mice. mtDNA was isolated from skeletal muscle tissues. (**E**) BrdU incorporation was analysed by slot blot using an anti-BrdU antibody. EtBr-stained gel acts as a loading control. (**F**) Quantitation of (**E**). *n* = 3 mice of each genotype. (**G**–**I**) Motor functions are restored in mice following C4 treatment. Seven-week-old POLGA WT, Hetero, and Homo mice were intravenously treated four times with either vehicle or C4. (**G**) Body weight, (**H**) Grip strength and (**I**) Time spent on the rotarod were measured every 5th day post-treatment for 4 weeks. Mean ± SD. *n* = 6 mice of each genotype. Data represent the mean ± SD and were analysed using a two-way ANOVA statistical test (for **D**, **F**) and mixed-effects analysis (for **G**, **H**, **I**) with Tukey’s multiple comparison test.

**Figure EV5:**
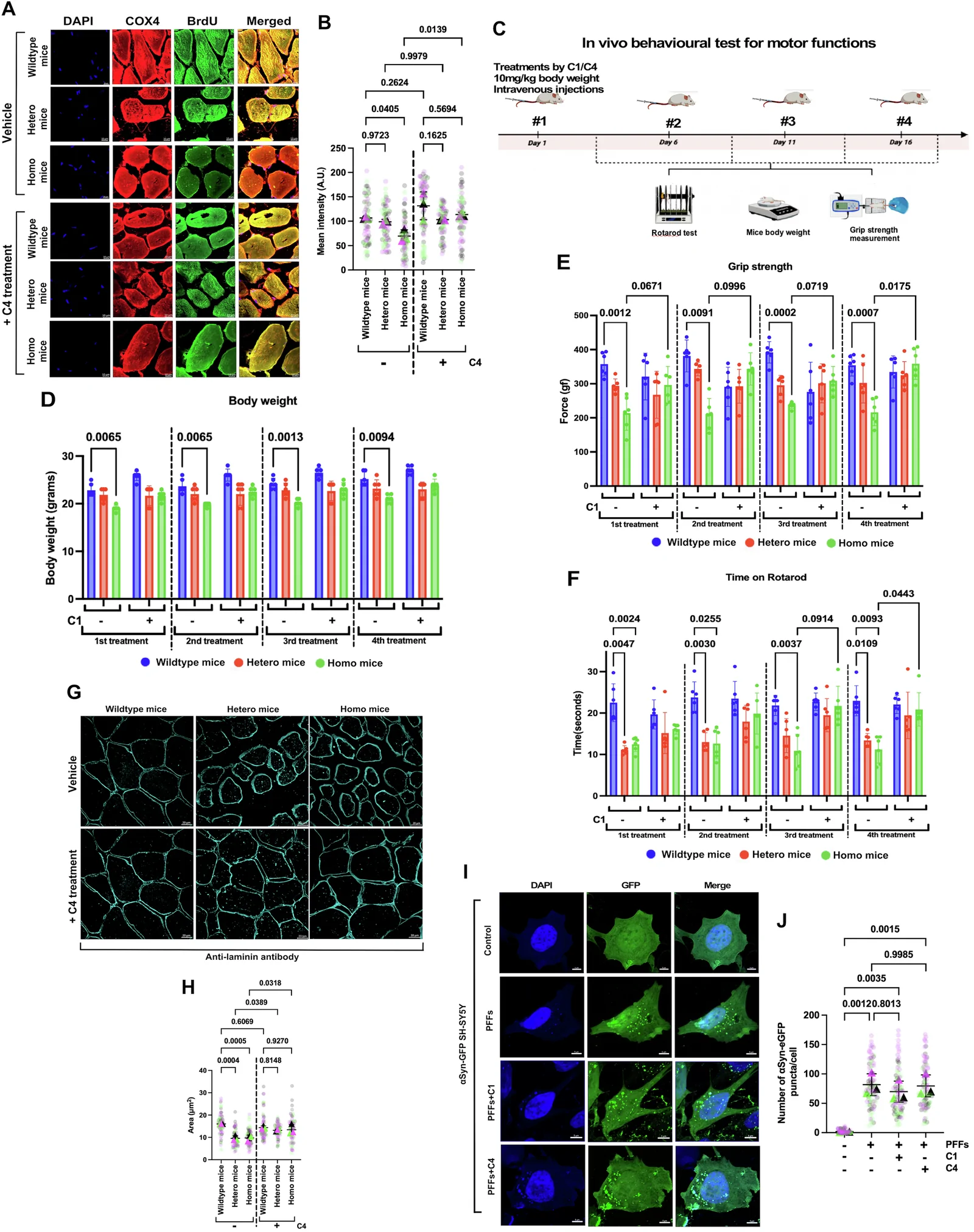
Treatment with C1 or C4 enhances multiple parameters of mitochondrial functions in the mice model. (**A**,** B**) C4 treatment restored mtDNA replication in the PEO mice model. (**A**) In vivo BrdU incorporation assay in mice skeletal muscle was analysed using immunofluorescence. Mice skeletal tissue sections from POLGA WT, Hetero (POLG^WT/A449T^), and Homo (POLG^A449T/A449T^) mice were stained with antibodies against BrdU and COX4. (**B**) BrdU signal intensity per muscle fibre was quantitated using SuperPlots. Mean ± SD. *n* = 70 muscle fibres across three biological replicates. (**C**) Schematic diagram depicting how POLGA WT, Hetero (POLG^WT/A449T^) and Homo (POLG^A449T/A449T^) mice were subjected to treatment with small molecules. (**D**–**F**) C1 treatment restored clinical phenotypes to the basal level in the PEO mice model. Seven-week-old POLGA WT, Hetero (POLG^WT/A449T^) and Homo (POLG^A449T/A449T^) mice were evaluated for clinical phenotypes, including (**D**) Body weight, (**E**) Grip strength and (**F**) Time spent on the rotarod without or after treatment with C1. Measurements were performed on the day following injections for four weeks. Mean ± SD. *n* = 6 mice. (**G**,** H**) C4 treatment enhances muscle fibre areas in Hetero (POLG^WT/A449T^) and Homo (POLG^A449T/A449T^) mice. (**G**) Same as (**A**), except the muscle fibre area was analysed using an antibody against laminin. Scale bar = 20 μm. (**H**) Quantitation of (**G**) using SuperPlots. *n* = 70 muscle fibres over three biological replicates. (**I**,** J**) C1 or C4 treatment do not disrupt α-synuclein aggregate formation in pre-formed fibrils (PFF)-treated ɑSyn-GFP SH-SY5Y cells. Immunofluorescence followed by confocal microscopy was performed in stably expressing ɑSyn-GFP SH-SY5Y cells, which were either untreated or treated with PFFs followed by treatment with C1 or C4. The nucleus was stained with DAPI. Scale bar = 5 μm. (**J**) Quantitation of (**I**) using SuperPlots. *n* = 84 cells across 3 biological replicates. Data represent the mean ± SD and were analysed using ordinary one-way (for **B**, **H**, **J**) and mixed-effects analysis (for **D**, **E**, **F**) statistical tests with Tukey’s multiple comparisons test.

To validate the functions of the small molecules in vivo, we utilised knock-in mice corresponding to the human A467T (PEO 2) mutation [three different mice genotypes analysed, namely POLGA WT (POLG^A449/A449^), Hetero (POLG^A449/A449T^) and Homo (POLG^A449T/A449T^)] (Silva-Pinheiro et al, 2021). First, we investigated the ubiquitylation status of the PEO mutants post C1 and C4 treatment of the three mice genotypes in their respective skeletal muscle extracts. PEO mutants were hyperubiquitylated in the skeletal muscle extracts of Hetero and Homo mice compared to WT mice, which was reversed upon treatment with small molecules (Fig. 6A). The formation of ubiquitylated PEO aggregates (as determined by 1D SDD-AGE), which were enhanced in Hetero and Homo mice, was reduced to near POLGA WT levels upon C1 and C4 treatment (Fig. 6B). Similar results were recapitulated by immunofluorescence, which showed decreased aggregate formation in both Hetero and Homo mice upon C4 treatment (Fig. 6C,D). To understand whether the disrupted PEO mutants can now enter the mitochondria and affect mtDNA replication in vivo, a BrdU incorporation assay was carried out with mtDNA isolated from the skeletal muscles of the mice without or after C4 treatment. Significant rescue in the levels of BrdU incorporation was seen in Homo mice (Fig. 6E,F). In parallel, an immunofluorescence assay carried out on the skeletal muscle tissues also showed enhanced BrdU incorporation in Homo mice upon C4 treatment (Fig. EV5A,B).

We next sought to investigate the status of the physiological parameters of the three mice genotypes without or after four intravenous injections of C1 or C4 every 5th day (Fig. EV5C).

Three-month-old homozygous mice have shown a 19.6% reduction in treadmill endurance and about a 40% reduction in spontaneous rearing compared to wild-type littermates (Silva-Pinheiro et al, 2021). In contrast to the earlier findings reported, which did not observe significant differences in body weight or rotarod performance in homozygous mice at ~3 months of age, we observed significant differences in body weight, grip strength, and rotarod performance in both hetero and homo mice at 7 weeks of age (Fig. 6G). Treatment with either C4 increased all three tested parameters—i.e., body weight (Fig. 6G), grip strength (Fig. 6H) and time spent on rotarod (Fig. 6I). Similarly, C1 significantly increased grip strength (Fig. EV5E) and time spent on rotarod (Fig. EV5F), but no significant improvement in body weight was observed (Fig. EV5D). Furthermore, an immunofluorescence experiment with an anti-Laminin antibody indicated the reversal of the muscle fibre area in Hetero and Homo mice following the 4th C4 treatment (Fig. EV5G,H), which probably allowed the regaining of motor functions in these PEO 2 mutant mice.

Finally, we evaluated the efficacy of C1 and C4 in an alternate model of protein aggregation, namely the alpha-synuclein (αSyn) aggregation model. Treatment with C1 and C4 did not result in any significant change in the number of α-synuclein puncta per cell (Fig. EV5I,J), indicating that the aggregate-disrupting effects of C1 and C4 are specific to PEO aggregates.

## Discussion

Mutations in POLGA can lead to a range of disorders, like ptosis and PEO, impacting both neurological and non-neurological systems (Lv et al, 2020; Nurminen et al, 2017; Van Goethem et al, 2001). We have recently demonstrated that in a subset of PEO patients, dysfunctional POLGA was due to its hyperubiquitylation by mitochondria E3 ligase MITOL, and the formation of aggregate-like structures that prevented it from entering the mitochondrial matrix and carrying out mtDNA replication (Hussain et al, 2021). Using cellular, biochemical and biophysical studies, we now provide evidence that the formation of PEO aggregates is a common phenomenon. Further, upon screening the Prestwick library consisting of FDA/EMA/PMDA-approved small molecules using a GFP-based protein complementation assay, we identified two small molecules, Diltiazem hydrochloride (C1) and Sulbactam (C4), which disrupted the PEO aggregates by inducing conformational changes in the PEO mutant proteins and led to enhanced import of the mutant POLGA into the mitochondrial matrix. The increased mitochondrial import following C1 and C4 treatment resulted in improvement in mtDNA replication (demonstrated by BrdU incorporation in a slot blot assay) and in intrinsic polymerase activity in vitro (shown by a primer extension assay), which possibly culminated in enhanced mtDNA content in PEO mutant-expressing cells. Although small molecule treatment rescued the defective polymerisation activity and increased mtDNA content, it remains unclear whether this rescue also represents restoration of the overall fidelity of mtDNA replication. It will be important to determine whether the compounds also restore exonuclease activity and consequently, whether the newly accumulated mtDNA is replicated with improved fidelity or continues to harbour elevated levels of misincorporation-derived errors.

A subset of PEO-associated POLGA mutants has previously been shown to undergo hyperubiquitylation (Hussain et al, 2021). Our findings extend the earlier observation to additional PEO mutants. Treatment with C4 reduced the elevated ubiquitylation of these mutant proteins. Notably, C4 did not interact with MITOL and did not affect its intrinsic E3 ligase activity, suggesting that the reduction in hyperubiquitylation is unlikely to result from direct inhibition of the ligase activity. Instead, based on evidence provided here, it is highly likely that C1/C4 modulated the conformation of the PEO mutants and/or their interaction with MITOL, thereby reducing their susceptibility to hyperubiquitylation.

**Figure 7:**
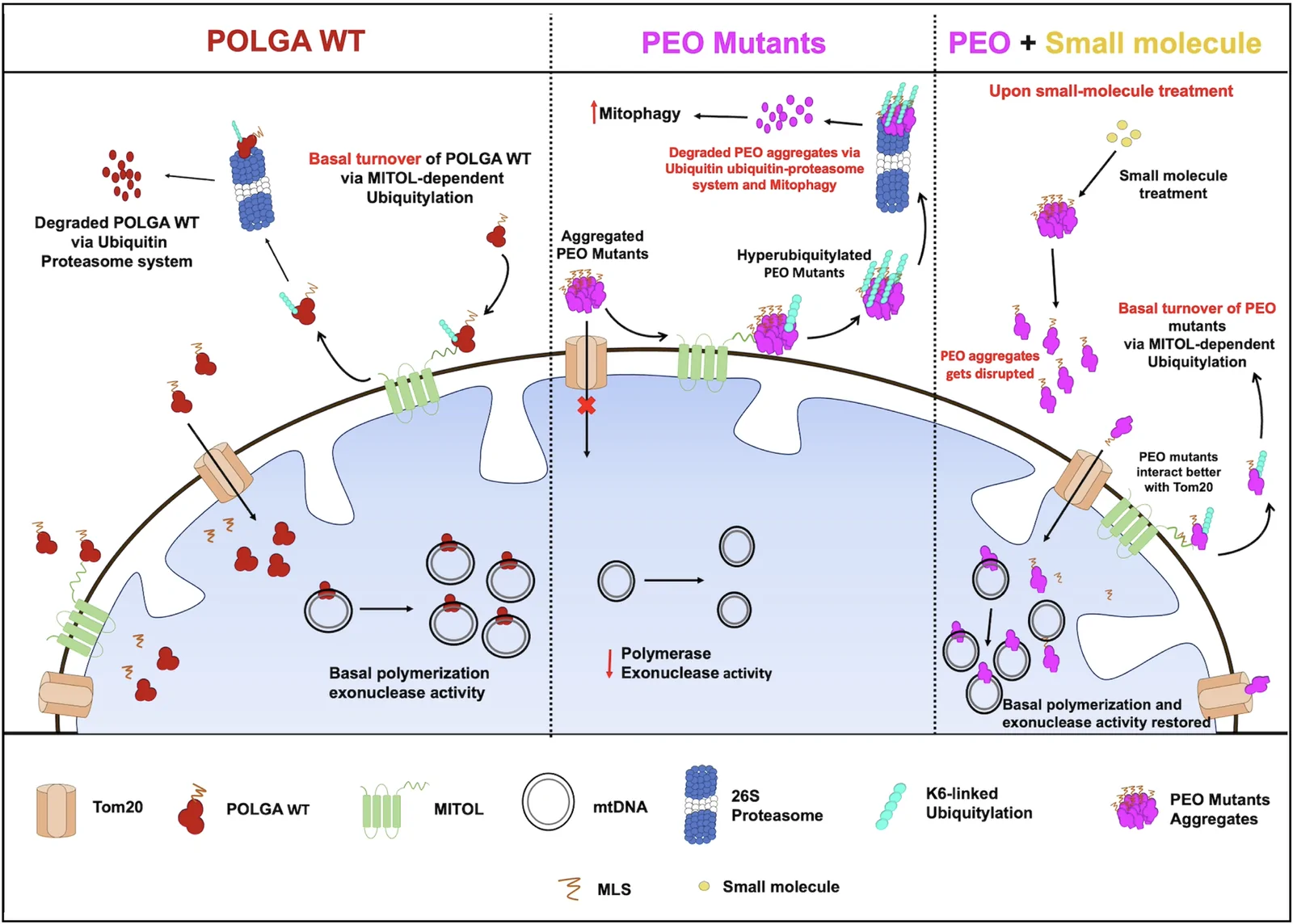
Schematic diagram showing the effect of small molecules on the reactivation of PEO mutants. (Left) MITOL ubiquitinates POLGA WT to a basal level. While ubiquitylated POLGA WT undergoes basal-level turnover, non-ubiquitylated POLGA WT can enter the mitochondrial matrix and carry out mtDNA replication. (Middle) MITOL hyperubiquitylates PEO mutants. The hyperubiquitylated PEO mutants show decreased interaction with Tom20 and form aggregates on the mitochondrial surface. The impaired mitochondrial import of the PEO mutant results in compromised mtDNA replication and destabilised mitochondrial homoeostasis. This destabilisation induces an upregulation of mitochondrial fission and clearance of damaged mitochondria via mitophagy. (Right) Treatment of PEO mutant-expressing cells with small molecules leads to restoration of mitochondrial function. Treatment with C1 and C4 leads to a change in the conformation of PEO mutants, disrupting PEO-PEO aggregates. This resulted in mitochondrial import of the PEO mutants, which restored mtDNA integrity and mitochondrial respiration, thereby enhancing mitochondrial homoeostasis to near-optimal levels.

Usage of C4 led to the activation of one of the most common PEO mutants, A467T, to near wild-type POLGA levels not only in cells but also in skeletal muscles of the mutant knock-in mice. The phenotypes observed in our A449T mice differ in some respects from those reported by (Silva-Pinheiro et al, 2021). In that study, homo mice did not show significant differences in body weight or rotarod performance at ~3 months of age, whereas we detected differences in body weight, grip strength, and rotarod performance in both hetero and homo mice at 7 weeks of age. In addition, differences in sex, genetic background, housing/husbandry conditions, and behavioural assay protocols may contribute to differences in phenotype between studies. Indeed, phenotypic variability arising from environmental and facility-specific factors has been documented even in genetically homogeneous mouse populations (Kovacs and Pearce, 2013) (Jaric et al, 2022) (Kentner et al, 2021). Therefore, the differences between our findings and those of (Silva-Pinheiro et al, 2021) should not necessarily be considered contradictory, but may possibly reflect differences in the age and experimental context in which the A449T phenotype was assessed. Post-treatment with C1 and C4, both the homozygous and heterozygous PEO mice were able to regain their motor functions almost to the same level as their wild-type counterparts (Fig. 7).

An unexpected finding is that heterozygous animals also show accumulation of aggregates outside mitochondria. This was surprising given the strictly recessive nature of the A467T mutation. However, the accumulation of these aggregates in heterozygous animals may not be sufficient to cause a phenotype, either because it does not interfere with cellular function or because its effects are compensated by the WT allele. In homozygous animals, this compensatory mechanism is absent, potentially leading to the accumulation of aggregates and, ultimately, mtDNA instability. We therefore interpret the A467T/WT phenotype as an early biochemical/mitochondrial vulnerability rather than a dominant disease phenotype. Age, tissue-specific mitochondrial stress and secondary mtDNA abnormalities may further influence phenotypic expression. Importantly, the heterozygous phenotype was not uniformly equivalent to the homozygous phenotype across all endpoints. We have avoided extrapolating the mouse heterozygous phenotype directly to human clinical disease.

Taken together, C1/C4 treatment possibly prevented the recognition of mutant proteins by MITOL, thereby reversing their hyperubiquitylation, preventing aggregate formation and consequently restoring their mitochondrial import. But these effects are unlikely to fully account for the observed functional rescue. This is because C1/C4 also potentiated the in vitro polymerisation activity of the mutant proteins, which contributed to the restoration of mitochondrial DNA (mtDNA) replication and the maintenance of mitochondrial homoeostasis. Thus, the effects of these two compounds may reflect a combination of multiple factors, including changes in PEO mutant conformation, their interaction with MITOL, mitochondrial import and improvement in polymerase activity.

PEO is one of the most common symptoms of mitochondrial disorder (Hirano and Pitceathly, 2023), ~20% of all the diagnosed patients with mitochondrial disorders were afflicted with PEO. There is no permanent cure for PEO patients other than symptomatic treatment (like surgery) (Eshaghi et al, 2021) and supplements (like CoQ10, B-vitamins, and antioxidants) to enhance mitochondrial functions and reduce oxidative stress (Ahmed et al, 2018). Based on the above results, C4 and C1 may represent potential therapeutic candidates for the treatment of POLGA-associated PEO; however, further studies are required to validate their efficacy and safety before clinical translation. Both C4 and C1 are FDA/EMA/PMDA-approved small molecules and do not have obvious deleterious effects. While C4 is an effective beta-lactamase inhibitor and is used in combination with other antibiotics, C1 is a calcium channel blocker that relaxes the blood vessels, lowers blood pressure, and increases the supply of blood and oxygen to the heart while reducing its workload. Though mild side effects are present for both, neither of these small molecules has such deleterious effects that prevent them from being used in severely afflicted PEO patients.

Recently, using a hit-to-lead optimisation approach, a small molecule (PZL-A) has shown the potential to restore the functions of multiple POLGA mutants seen in PEO (Valenzuela et al, 2025). PZL-A carries out its functions by binding to an allosteric site at the interface between the catalytic POLGA subunit and the proximal POLGB subunit. It was shown that PZL-A activates mtDNA synthesis—both in vitro and in cells. While the end result for all the compounds may be the same, the mechanism by which C1 and C4 activate POLGA is different compared to PZL-A. Unlike PZL-A, C1 and C4 disrupt the aggregate formed by the PEO mutants, thereby allowing the mutant PEO to enter mitochondria and activate mtDNA functions and restore mitochondrial homoeostasis to wild-type levels. Further, the functioning of C1 and C4 (unlike in the case of PZL-A) has been validated in both cells and mice. In fact, Sulbactam (C4) or in newer formulations like Sulbactam-durlobactam are the subject of a large number of completed and ongoing clinical trials (https://clinicaltrials.gov/search?intr=Sulbactam). The major advantage of C1 and C4 has been their validation in PEO mutant knockin mice, where motor functions of the animals were found to be restored. Interestingly, modelling of POLGA mutations in mice has been shown to provide crucial and accurate insights into the pathogenesis of PEO and is postulated to be a robust model for therapeutic development (Corra et al, 2025). It will also be interesting to know whether our identified small molecules can reverse the integrated stress response and disrupt the mitochondrial folate cycle as seen in a muscle-specific POLGA exonuclease-deficient mice model (Bond et al, 2025).

Protein conformational states change in response to cellular changes, intrinsic mutations, and misfolding, which often lead to exposed hydrophobic aggregation-prone regions interacting with other exposed hydrophobic regions, leading to protein-protein interactions resulting in aggregation (De Baets et al, 2015; Hartl et al, 2011; Tamas et al, 2014; Vabulas et al, 2010). Protein aggregation is triggered by stressful conditions like high temperature and oxidative stress, leading to protein misfolding and aggregation, a phenomenon associated with over 50 human diseases (Iadanza et al, 2018). Proper folding during translocation and refolding is essential to prevent misfolding and aggregation in the mitochondria, which results in clogging the translocases (Weidberg and Amon, 2018) (Kang et al, 1990) (Wang and Chen, 2015) (Wrobel et al, 2015). The small molecule-based approach has shown the potential to disrupt or inhibit amyloid β oligomerisation (Daval et al, 2010; Necula et al, 2007). Recently, the tau protofibril, found in chronic traumatic encephalopathy, was disrupted using quercetin and gallic acid (Tang et al, 2023). Mitochondrial protein import clogging has been suggested to be one of the mechanisms of diseases, including adPEO in humans (Coyne et al, 2023). Recently, an inducible mitochondria-specific protein degradation system has been demonstrated to be effective in vivo in human cells (Sanyal et al, 2024). Thus, our present study adds to the growing concept that reactivation of the mitochondria in mitochondrial disorders like PEO would be an effective way for future therapy. Incidentally, the POLGA A467T mutation is also implicated in other mitochondrial disorders like Alpers syndrome and juvenile spinocerebellar ataxia-epilepsy syndrome. Hence, reactivation of mutant POLGA with small molecules may have therapeutic benefits for patients afflicted with other mitochondrial disorders where mtDNA replication is compromised. However, the use of C1 and C4 could not rescue alpha-synuclein aggregates, indicating that a certain amount of specificity is present in the functioning of these two small molecules.

Taken together, the present findings offer encouraging preclinical evidence supporting further investigation of therapeutic strategies for PEO. Nevertheless, additional work is required to assess the potential for clinical translation, including evaluation of pharmacokinetic properties and long-term safety, and determination of whether the response is consistent across different POLG variants. Such studies will be essential to strengthen the therapeutic relevance and potential clinical applicability of these findings.

## Methods

**Table:** Reagents and tools table

Reagent/resource	Reference or source	Identifier or Catalogue Number
**Experimental models**		
HEK293T	Present in the lab	ATCC Cat# CRL-3216
HCT116 POLGA CRISPR KO	Prof. Dr. Meltem Muftuoglu, Acibadem Mehmet Ali Aydinlar University, Istanbul, Turkey	(Somuncu et al, 2022)
HCT116 POLGA YFP HA	This study	N/A
HCT116 PEO 2-YFP HA	This study	N/A
HCT116 PEO 6-YFP HA	This study	N/A
HeLa shGFP	Shigeru Yanagi (Tokyo University of Pharmacy and Life Sciences, Japan)	(Sugiura et al, 2013)
HeLa shMITOL	Shigeru Yanagi (Tokyo University of Pharmacy and Life Sciences, Japan)	(Sugiura et al, 2013)
αSyn-GFP SH-SY5Y	Prof. Dr. Tushar Kanti Maiti (RCB, Faridabad, India)	N/A
FVB POLGA WT (referred to as wild-type mice)	Carlo Viscomi (University of Padova, Italy)	(Silva-Pinheiro et al, 2021)
FVB POLGA^WT/A449T^ (referred to as heterozygous mice)	Carlo Viscomi (University of Padova, Italy)	(Silva-Pinheiro et al, 2021)
FVB POLGA^A449T/A449T^ (referred to as homozygous mice)	Carlo Viscomi (University of Padova, Italy)	(Silva-Pinheiro et al, 2021)
**Recombinant DNA**		
pLVX-IRES-Hyg-POLGA-YFP-HA (referred to as POLGA-YFP HA)	This study	N/A
pLVX-IRES-Hyg-POLGA-GFP11 (referred to as POLGA-GFP11)	This study	N/A
pLVX-MITOL-YFP	This study	N/A
pLVX-Tom20-YFP	This study	N/A
pLVX-IRES-Hyg-CFP-Flag	This study	N/A
pLVX-IRES-POLGA-CFP-Flag	This study	N/A
pLVX-IRES-Hyg-YFP-HA	This study	N/A
pHAGE-mt-mKeima	Addgene (Cat# 131626)	(Vargas et al, 2019)
pHR-SFFV-GFP1-10	Addgene (Cat# 80409)	(Kamiyama et al, 2016)
pcDNA3.1GFP (1–10)	Addgene (Cat# 70219)	(Kamiyama et al, 2016)
pCMV6-Myc-DDK-PEO 5 (K1060R)	This study	N/A
pCMV6-Myc-DDK-PEO 6 (K1060R)	This study	N/A
pCMV6-Myc-DDK-PEO 7 (K1060R)	This study	N/A
pCMV6-Myc-DDK-PEO 5	This study	N/A
pCMV6-Myc-DDK-PEO 6	This study	N/A
pCMV6-Myc-DDK-PEO 7	This study	N/A
pLVX-IRES-Hyg-PEO 2-YFP-HA (referred to as PEO 2-YFP HA)	This study	N/A
pLVX-IRES-Hyg-PEO 6-YFP-HA (referred to as PEO 6-YFP HA)	This study	N/A
pLVX-IRES-Hyg-PEO 5-CFP-FLAG (referred to as PEO 5-CFP)	This study	N/A
pLVX-IRES-Hyg-PEO 6-CFP-FLAG (referred to as PEO 6-CFP)	This study	N/A
pLVX-IRES-Hyg-PEO 7-CFP-FLAG (referred to as PEO 7-CFP)	This study	N/A
pLVX-MITOL-YFP (referred to as Mitol-YFP)	This study	N/A
pLVX-Tom20-YFP (referred to as Tom20-YFP)	This study	N/A
pLVX-IRES-Hyg-PEO 2-GFP11 (referred to as PEO 2-GFP11)	This study	N/A
pGEX4T-1-POLGA (53-1239) (referred to as GST-POLGA)	Present in the lab	(Hussain et al, 2021)
pGEX4T-1-PEO 2 (53-1239) (referred to as GST-PEO 2)	Present in the lab	(Hussain et al, 2021)
pGEX4T-1-PEO 5 (53-1239) (referred to as GST-PEO 6)	This study	N/A
pGEX4T-1-PEO 6 (53-1239) (referred to as GST-PEO 5)	This study	N/A
pGEX4T-1-PEO 7 (53-1239) (referred to as GST-PEO 7)	This study	N/A
pET28b-His-POLGA (53-1239) (referred to as His-POLGA)	Present in the lab	(Hussain et al, 2021)
pET28b-His-PEO 2 (53-1239) (referred to as His-PEO 2)	Present in the lab	(Hussain et al, 2021)
pET28b-His PEO 5 (53-1239) (referred to as His-PEO 5)	This study	N/A
pET28b-His PEO 6 (53-1239) (referred to as His-PEO 6)	This study	N/A
pET28b-His PEO 7 (53-1239) (referred to as His-PEO 7)	This study	N/A
pcDNA3-Flag-POLGA	Present in the lab	(Hussain et al, 2021)
pcDNA3-Flag-PEO 2	Present in the lab	(Hussain et al, 2021)
pcDNA3-Flag-PEO 5	This study	N/A
pcDNA3-Flag-PEO 6	This study	N/A
pcDNA3-Flag-PEO 7	This study	N/A
pcDNA3-Flag-PEO 2 K1060R	Present in the lab	(Hussain et al, 2021)
pGEX4T-1-Mitol (referred to as GST-Mitol)	Present in the lab	(Hussain et al, 2021)
**Antibodies**		
Hsp60, Rabbit polyclonal (used for WB)	Abcam	Cat# ab46798
MITOL, Rabbit polyclonal (gift from Dr. Shigeru Yanagi, Associate Prof, Tokyo University) (used for WB)	(Yonashiro et al, 2006)	N/A
Anti-Ubiquitin (P4D1), Mouse Monoclonal (used for WB)	Santa Cruz Biotechnology	Cat# Sc 8017
Flag M2, Mouse Monoclonal (used for WB)	Merck	Cat# F1804
Anti-FLAG M2 Affinity Gel (for IP)	Millipore	Cat# A2220
Anti-BrdU, Sheep Polyclonal (used for WB, IF)	Abcam	Cat# ab1893
p62 (D5E2), Rabbit Monoclonal (used for WB)	Cell Signaling Technology	Cat# 8025
NDP52 (D1E4A), Rabbit Monoclonal (used for WB)	Cell Signaling Technology	Cat# 60732
Optineurin (D2L8S), Rabbit monoclonal (used for WB)	Cell Signaling Technology	Cat# 58981
Parkin (Prk8), Mouse monoclonal (used for WB, IF)	Cell Signaling Technology	Cat# 4211
PINK1(D8G3), Rabbit monoclonal (used for WB)	Cell Signaling Technology	Cat# 6946
BNIP3 (D7U1T), Rabbit monoclonal (used for WB)	Cell Signalling Technology	Cat# 44060
BNIP3L/Nix (D4R4B), Rabbit monoclonal (used for WB)	Cell Signalling Technology	Cat# 12396
LC3B (D11), Rabbit monoclonal (used for WB, IF)	Cell Signalling Technology	Cat# 3868
DNA PolG antibody (G-6), Mouse monoclonal (used for WB, IP)	Santa Cruz Biotechnology	Cat# Sc 390634
HA-tag antibody (C29F4), Rabbit monoclonal (used for WB, IP)	Cell Signalling Technology	Cat# 3724
DNA polymerase gamma antibody, Rabbit polyclonal (used for WB, IP)	Novus Biologicals	Cat# NBP1-33633
Full-Length A.v. Polyclonal Antibody, Rabbit polyclonal, living colours antibody (used for YFP) (used for WB, IP)	Takara	Cat# 632460
Tom20 (D8T4N), Rabbit monoclonal (used for WB)	Cell Signalling Technology	Cat# 42406
OPA1 (D6U6N), Rabbit monoclonal (used for WB)	Cell Signalling Technology	Cat# 80471
DRP1 (D6C7), Rabbit monoclonal (used for WB)	Cell Signalling Technology	Cat# 8570
Phospho-DRP1 (Ser616) (D9A1), Rabbit monoclonal (used for WB)	Cell Signalling Technology	Cat# 4494
Phospho-DRP1 (Ser637) (D3A4), Rabbit monoclonal (used for WB)	Cell Signalling Technology	Cat# 6319
MFF (E5W4M), Rabbit monoclonal (used for WB)	Cell Signalling Technology	Cat# 84580
MFN-1 (D6E2S), Rabbit monoclonal (used for WB)	Cell Signalling Technology	Cat# 14739
MFN2 (D1E9), Rabbit monoclonal (used for WB)	Cell Signalling Technology	Cat# 11925
Anti-Laminin, Rabbit antibody (used for IF)	Sigma-Aldrich	Cat# L9393
Anti-ATG12 antibody, Rabbit Polyclonal (used for WB)	Abcam	Cat# ab155589
Anti-Cytochrome C antibody, Mouse monoclonal (used for IF)	Novus Biologicals	Cat# NB100-56503
COX IV antibody, Rabbit antibody (used for IF)	Cell Signalling Technology	Cat# 4844
Myc-Tag (71D10), Rabbit monoclonal antibody (used for WB)	Cell Signalling Technology	Cat# 2278
Anti-His, Mouse monoclonal antibody (used for WB)	Santa Cruz Biotechnology	Cat# Sc 8036
Anti- β actin, Mouse monoclonal antibody (used for WB)	Santa Cruz Biotechnology	Cat# Sc 47778
Anti-GST, Rabbit antibody (used for WB)	Thermo Fisher Scientific	Cat# 71-7500
DNA Polymerase gamma Antibody; Rabbit Polyclonal (used for IF)	Thermo Fisher Scientific	Cat# BS-13017R
Anti-RECQL4 (used for WB, IP)	Santa Cruz Biotechnology	Cat# sc-366840
**Oligonucleotides and other sequence-based reagents**		
Cloning primers	This study	Table EV3
Genotyping PCR primer	(Silva-Pinheiro et al, 2021)	Table EV3
Long-range mtDNA amplification primer	(Chi et al, 2012)	Table EV3
Primer extension assay primer	(Gupta et al, 2014; Hussain et al, 2021)	Table EV3
Exonuclease assay primer	(Gupta et al, 2014)	Table EV3
mtDNA copy number primer	N/A	Table EV3
**Chemicals, enzymes and other reagents**		
IPTG	Merck	Cat# I6758; CAS Number 367-93-1
Paraformaldehyde	Merck	Cat# 158127; CAS Number 30525-89-4
PMSF	Merck	Cat# P7626; CAS Number 329-98-6
Acrylamide	Merck	Cat# 110784; CAS Number 79-06-1
DTT	Merck	Cat# D0632; CAS Number 3483-12-3
Sodium deoxycholate	Merck	Cat# D6750; CAS Number 302-95-4
Tween 20	Sigma-Aldrich	Cat# P7949; CAS Number 9005-64-5
Triton X-100	Merck	Cat# T9284; CAS Number 9002-93-1
ATP	Merck	Cat# A9187; CAS Number 74804-12-9
DMSO	Merck	Cat# D2650; CAS Number 67-68-5
PMSF	Sigma-Aldrich	Cat# P7626; CAS Number 329-98-6
Imidazole	Bio Basic	Cat# IB0277; CAS Number 288-32-4
Lysozyme	Sigma-Aldrich	Cat# L4919; CAS Number 12650-88-3
Proteinase K	Bio Basic	Cat# PB0451; CAS Number 39450-01-6
L-Glutathione reduced	Merck	Cat# G6529; CAS Number 70-18-8
N, N′-Methylenebisacrylamide	Sigma-Aldrich	Cat# M7279; CAS Number 110-26-9
Puromycin	Sigma-Aldrich	Cat# P8833; CAS Number 58-58-2
HEPES	Sigma-Aldrich	Cat# H3375; CAS Number 7365-45-9
EDTA	Merck	Cat# 324503; CAS Number 6381-92-6
EGTA	Merck	Cat# 324626; CAS Number 67-42-5
Na_2_HPO_4_	Merck	Cat# 106559; CAS Number 7558-79-4
KCl	Merck	Cat# 104936; CAS Number 7447-40-7
NaCl	Merck	Cat# 106406; CAS Number 7647-14-5
MgCl_2_	Sigma-Aldrich	Cat# 814733; CAS Number: 7786-30-3
BSA	Sigma-Aldrich	Cat# SRE0098; CAS Number: 9048-46-8
2-Mercaptoethanol	Sigma-Aldrich	Cat# 63689; CAS Number: 60-24-2
SDS	Sigma-Aldrich	Cat# L3771; CAS Number: 151-21-3
Tris(hydroxymethyl)aminomethane	Millipore	Cat# 108382; CAS Number: 77-86-1
Glycine	Millipore	Cat# 3570; CAS Number: 56-40-6
Sucrose	Sigma-Aldrich	Cat# S9378; CAS Number: 57-50-1
Methanol	Merck	CAS Number: 67-56-1
CCCP	Sigma-Aldrich	Cat# C2759; CAS Number 555-60-2
BrdU	Sigma-Aldrich	Cat# B5002; CAS Number 59-14-3
IPTG	Sigma-Aldrich	Cat# I6758; CAS Number 367-93-1
MG132	Merck	Cat# 474787; CAS Number 133407-82-6
Uridine	Sigma-Aldrich	Cat# U3003; CAS Number 58-96-8
Verapamil hydrochloride	Merck	CAS Number 152-11-4
Tazobactam	Merck	CAS Number 89786-04-9
Clavulanic acid	Merck	CAS Number 58001-44-8
D (-)-Mannitol Pure	Merck	Cat# M9647; CAS Number 69-65-8
Ampicillin	Bio Basic	Cat# AB0028
Kanamycin	Bio Basic	Cat# KB0286
Hygromycin B	Sigma-Aldrich	Cat# H7772
Penicillin-Streptomycin-Amphotericin B Solution	Biological industries	Cat# 03-033-1B
KH_2_PO_4_	Merck	Cat# 104873; CAS Number 7778-77-0
Ethanol	Merck	Cat# 100983; CAS Number 64-17-5
Glycerol	Merck	Cat# 107051; CAS Number 56-81-5
TEMED	Bio-Rad	Cat# 1610801
Ammonium Persulfate	Sigma-Aldrich	Cat# A3678; CAS Number 7727-54-0
Coomassie brilliant blue-R-250	Bio-Rad	Cat# 1610400
Ethidium bromide (EtBr)	Sigma-Aldrich	Cat# E7637
Luria Bertani Broth, Miller	Himedia	Cat# M1245
Luria Bertani Agar, Miller	Himedia	Cat# M1151
Skim milk powder	Himedia	Cat# GRM1254
Agarose	Sigma-Aldrich	Cat# A9539
Foetal bovine Serum	Thermo Fisher Scientific	Cat# 10082147
Advanced DMEM	Thermo Fisher Scientific	Cat# 12491-023
DMEM/F-12 medium	Thermo Fisher Scientific	Cat# 11320033
Horse serum	Thermo Fisher Scientific	Cat# 16050130
OptiMem reduced serum medium	Thermo Fisher Scientific	Cat# 31985-088
Complete Protease Cocktail inhibitor	Roche	Cat# 116974980
BL21 (DE3) competent cells	Thermo Fisher Scientific	Cat# EC0114
Poly-Prep Chromatography column	Bio-Rad	Cat# 73101550
Mounting medium with DAPI	Vector Laboratories	Cat# H-1200
Nhe1-HF	New England BioLabs	Cat# R3131
Not1-HF	New England BioLabs	Cat# R3189
Spe1-HF	New England BioLabs	Cat# R3133
XhoI	New England BioLabs	Cat# R0146
BamHI-HF	New England BioLabs	Cat# R3136
HindIII-HF	New England BioLabs	Cat# R3104
Glutathione S- Sepharose beads	Cytiva	Cat# 17-5279-02
ANTI-FLAG® M2 Affinity Gel	Sigma-Aldrich	Cat# A2220
Sepharose™ CL-6B	Sigma-Aldrich	Cat# CL6B200
3X Flag peptide	Sigma-Aldrich	Cat# F4799
Lipofectamine 2000	Thermo Fisher Scientific	Cat# 11668019
LongAmp Taq DNA polymerase	New England Biolabs	Cat# M0323L
Immobilon Western Chemiluminescent HRP substrate	Merck	Cat# WBKLS0500
10Χ PBS	Sartorius	Cat# 02-023-5 A
Trypsin (2.5%)	Thermo Fisher Scientific	Cat# 15090046
L-Glutamine solution	Biological industries	Cat# 03-020-1B
Prestained Low Molecular weight markers	Bio-Rad	Cat# 1610374
Wide range DNA marker	Sigma-Aldrich	Cat# D7058
Nitrocellulose membrane	Bio-Rad	Cat# 1620115
Streptavidin Agarose	Millipore	Cat# 69203
T7 Quick-coupled Transcription/ Translation system	Promega	Cat# L2080
**Software**		
GraphPad Prism 9	https://www.graphpad.com/	
ImageJ	https://imagej.net/Fiji	
Wave	https://www.agilent.com/en/product/cell-analysis/real-time-cell-metabolic-analysis/xf-software/seahorse-wave-desktop-software-740897	
FlowJo v10	https://www.flowjo.com/	
**Other**		
HOOK™-Biotin-PEG2-Amine Labelling Kit	G-Biosciences	Cat# BS-16
Mitochondrial DNA isolation kit	BioVision Inc.	Cat# K280-50
QuikChange II XL Site-Directed Mutagenesis Kit	Agilent	Cat# 200522
QIAamp DNA Mini Kit	Qiagen	Cat# 51304
QIAamp DNA Midi Kit	Qiagen	Cat# 12143

### Reagents, antibodies, primers and recombinants

All antibodies, reagents (including chemicals, recombinant proteins, cell lines, peptides and oligonucleotides), animals, deposited data and other materials used are described in structured methods. All primers used are described in Table EV3.

### Cell culture

HEK293T, HeLa shGFP and HeLa shMITOL cell lines were maintained and cultured in Dulbecco’s modified Eagle’s medium supplemented with 10% foetal bovine serum, 1% L-glutamine and 1% Penicillin-Streptomycin-Amphotericin B solution. Plasmid transfections were carried out using Lipofectamine 2000 according to the manufacturer’s protocol for 24 to 48 h. For Western and immunofluorescence analysis, 0.75 to 1 μg of Flag-tagged POLGA WT or PEO mutant plasmid DNA was transfected into HEK293T cells using Lipofectamine 2000 according to the manufacturer’s protocol. POLGA CRISPR KO cells were cultured in DMEM/F12 medium (10% foetal bovine serum, 1% Penicillin-streptomycin-amphotericin B, 0.2 mM uridine and 0.5 mM sodium pyruvate). All cell lines were maintained at 37 °C and 5% CO_2_. To isolate the soluble and insoluble fractions, the transfected HEK293T cells were lysed in a lysis buffer (50 mM Tris-HCl, pH 7.4, 150 mM NaCl, 5 mM EDTA, 1% Triton X-100 and protease inhibitor cocktail) by pipetting gently and rotating on an end-to-end rotor for 10 min at 4 °C. Soluble and insoluble fractions were separated by centrifugation at 20,000×*g* for 30 min at 4 °C (Hussain et al, 2021).

### Stable cell line generation

HCT116 POLGA-YFP-HA, HCT116 PEO 2-YFP-HA and HCT116 PEO 6-YFP-HA stable cell lines were generated by selection using 200 µg/mL hygromycin B. These stable cell lines were generated in a POLGA knockout HCT116 background cells which was created by the CRISPR/Cas9 method (Somuncu et al, 2022). For virus production, HEK293T cells were seeded at 60–70% confluency in a 10 cm plate. The lentiviral expression plasmids, namely pLVX-IRES-Hyg-YFP-HA, pLVX-IRES-Hyg-POLGA-YFP-HA, pLVX-IRES-Hyg-PEO 2-YFP-HA, pLVX-IRES-Hyg-PEO 6-YFP-HA, were co-transfected with packaging (pSPAX2) and envelop (pMD2.G) plasmids (in 4:3:1 plasmid molar ratio) using lipofectamine 3000 (in 1:2 ratio). Cells were supplemented with complete medium 6 h post-transfection. The viral supernatant was collected 24 h post-transfection and centrifuged at 800 x g for 10 min. The supernatant was concentrated using a 4Χ lentivirus concentrator [40% PEG-8000 (w/v) and 1.5 M NaCl in 1Χ PBS (pH 7.4)] in a 1:3 ratio and incubated on an end-to-end rotor for 10–12 h at 4 °C, followed by centrifugation at 1800×*g* for 1 h. The viral pellet was dissolved in 1Χ PBS. POLGA CRISPR KO cells were transduced in 12-well cluster plates using polybrene (10 μg/mL). Cells were supplemented with fresh medium 16 h post-transduction. Cells were selected with 200 μg/mL hygromycin for 14–16 days for stable integration of the transgene.

### Preparation and seeding of αSyn amyloid fibrils in αSyn-GFP SH-SY5Y cells

αSyn fibrils were prepared following a published protocol (Bisht et al, 2026). Seeds were prepared by sonicating fibrils using a probe sonicator at 20% amplitude for 60 s at 0.5-s intervals. αSyn-GFP SH-SY5Y cells were maintained in Dulbecco’s modified Eagle’s medium (DMEM) HI-Glucose with 10% FBS, 3.0 mM glutamine, 400 μg/mL G418, and 1X Pen-Strep antibiotic at 37 °C and 5% CO_2_. For seeding assays, cells were seeded at 60% confluency in 12-well plates with glass coverslips. Before treatment, cells were serum-starved for 12 h in incomplete media. Following starvation, 1 μM aSyn seeds or pre-formed fibrils (PFFs) in complete media were added to the cells and incubated for 72 h. Post-incubation, the media was discarded, and the cells on coverslips were fixed with 4% paraformaldehyde dissolved in 1X PBS for 10 min at RT. Washing was performed thrice with 1X PBS. The coverslips were mounted onto glass slides with a mounting medium with 4’, 6-diamidino-2-phenylindole (DAPI). Cells were imaged using Airyscan super-resolution mode in a Zeiss LSM 980 Airyscan confocal microscope with a 63x/1.4 oil objective lens. The quantification was done using ImageJ software.

### Immunoblotting

For immunoblotting, cell lysates were prepared using M2 lysis buffer (50 mM Tris, pH 7.4, 150 mM NaCl, 0.5 mM EDTA, 0.5 mM EGTA, 1% Triton X-100, 10% glycerol, 1X protease inhibitor cocktail (PIC) and 1 mM phenylmethylsulphonyl fluoride (PMSF). Tissue lysates were made using RIPA lysis buffer (1 mM Tris-HCl, pH 8.0, 50 mM NaCl, 2% Triton X-100, 1% (w/v) sodium deoxycholate, 0.1% (w/v) SDS, along with 1X PIC and 1 mM PMSF). Cell lysates (50 μg) or tissue lysates (60–80 μg) were mixed with reducing loading buffer (final concentrations: 62.5 mM Tris–Cl, pH 6.8, 4% SDS, 10% glycerol, 5% β-mercaptoethanol and 0.002% bromophenol blue) and boiled for 10 min. The samples were resolved in sodium dodecyl sulphate-polyacrylamide gel electrophoresis (SDS-PAGE) and subjected to immunoblotting with respective antibodies.

### Immunofluorescence

Cells were seeded on flame-sterilised 22 mm coverslips in the six-well plate. At 65–70% confluency, cells were fixed with 4% paraformaldehyde (in 1X PBS) for 30 min at room temperature. Post-fixation, cells were washed twice with 1X PBS and permeabilised with 0.1% Triton X-100 or 0.5% saponin in 1X PBS. The cells were blocked with 10% Normal Chicken Serum in 1X PBST (1X PBS + 0.2% Tween 20) for 1 h at room temperature, followed by washing with 1X PBST. Cells were incubated with primary antibody for 90 min at room temperature or overnight at 4 °C, followed by washing and incubation with secondary antibody for 1 h at room temperature. The coverslips were mounted onto glass slides with a mounting medium with DAPI. Cells were imaged using Airyscan super-resolution mode in a Zeiss LSM 980 Airyscan confocal microscope with a 63x/1.4 oil objective lens. The laser lines used were 445 nm (for CFP), 488 nm (for FITC), 514 nm (for YFP), diode 594 nm/DPSS 561 nm (for Mitotracker) and diode 405 nm (for DAPI). Data analysis was done using Zeiss Zen 3.8 software.

For tissue immunofluorescence, vastus lateralis (skeletal muscle) was exercised, washed with chilled 1X PBS and fixed in 4% Paraformaldehyde (made in 1X PBS) for 5–6 h at 4 °C. Muscle tissue was washed twice with 1X PBS and incubated with 30% sucrose at 4 °C for 16–18 h until the tissue sank. The frozen tissue blocks were prepared with Optimal Cutting Temperature (OCT) compound, and 7-μm-thick cryosections were transferred to poly-L-lysine-coated slides and utilised for subsequent staining. The slides were hydrated with wash buffer (0.5% Tween 20 in 1X PBS) followed by blocking with blocking buffer (2% bovine serum albumin (BSA) in 1X PBS) for 2 h at room temperature. Permeabilisation was done with 2% Triton X-100 in 1X PBS for 20 min at room temperature. Sections were washed twice with wash buffer for 5 min each and then stained with primary antibody in a dark, humidified chamber for 2 h at room temperature. Post-incubation sections were washed twice with wash buffer and incubated with secondary antibody for 1 to 2 h at room temperature. After two washes for 5 min each, sections were mounted with mounting medium (with DAPI). Quantification was done using FIJI software.

### Interactions and immunoprecipitations

For endogenous protein immunoprecipitation, 1 mg lysate was incubated with 1 µg of specific antibody (anti-POLGA or anti-RECQL4 or the corresponding IgG control) overnight at 4 °C on an end-to-end rotor. Post-incubation, 30 µL of Protein G Sepharose bead slurry (equilibrated with 1X PBS  +  0.1% NP-40) was added and incubated further for 3 h at 4 °C. After binding, the beads were washed three times with 1 mL of 1X PBS containing 0.1% NP-40. The reducing loading buffer was added to the beads and boiled for 12 min. Samples were resolved either in 10% SDS-PAGE gels or Nu-PAGE 4–12% gels using 1X MOPS buffer and transferred to a nitrocellulose membrane for subsequent immunoblotting.

In vitro interactions between MITOL-POLGA/PEO mutants and POLGA/PEO-POLGA/PEO were performed as described (Hussain et al, 2021). 750 ng of eluted His-tagged POLGA/PEO mutants were incubated with 500 ng of bead-bound GST-tagged MITOL for 6 h at 4 °C. Post-incubation, beads were washed twice with PBST (1X PBS + 0.1% NP-40). The samples were boiled after adding SDS sample buffer (final concentration 50 mM Tris-Cl, pH 6.8, 0.1% bromophenol blue, 10% glycerol, 2% SDS and 5% β-ME) and resolved on an 8% SDS-PAGE gel. The bound protein was detected by immunoblotting.

GST-tagged POLGA WT, GST-tagged PEO mutants, GST-tagged MITOL and His-tagged POLGA WT and PEO mutants proteins were expressed in *Escherichia coli* BL21(DE3) competent cells by adding 1 mM of Isopropyl-1-thio-β-D-galactopyranoside (IPTG) and incubating the culture at 180 rpm, 16 °C for 16 h, and subsequently purified according to standard protocols by binding to glutathione S-Sepharose beads (for GST-tagged protein) or Ni-NTA beads (for His-tagged protein). The bound proteins were eluted using a concentration gradient of reduced glutathione (0.5–5 mM) or imidazole (100–400 mM). The eluted fractions containing the purified protein were dialysed and used for the assays.

For Flag-tagged protein purification from mammalian cells, 3 μg of Flag-tagged POLGA WT and PEO mutant plasmids were transfected into HEK293T cells. The expression of Flag-tagged proteins was checked by immunoblotting using an anti-Flag antibody. Next, anti-Flag M2 beads (5 μL/IP) were mixed with 10 μL of Sepharose CL-6 beads and incubated with 2 mg of lysate on an end-to-end rotor for 150 min at 4 °C. The bead-protein complex was washed twice with the M2 lysis buffer for 4 min. The Flag-tagged protein-bound beads were incubated with Flag peptide (final concentration 100 μg/mL) for 90 min with rigorous tapping at 10-minute intervals. The centrifugation was done at 1400 rpm for 10 min at 4 °C, and the supernatant was collected. Purified eluted proteins were run on SDS-PAGE and checked by Coomassie brilliant blue staining.

### In vitro transcription cum translation and import assay

To assess the relative rate of import between POLGA WT and PEO mutants and to examine the effect of the C4 compound on the import of PEO mutants, an in vitro import assay was carried out. At first, in vitro transcription and translation were carried out with 2 µg of supercoiled DNA in the presence of cold methionine using T7 Quick-coupled Transcription/translation kit (Promega). The reaction was incubated at 30 °C for 90 min, after which the products were confirmed using SDS-PAGE followed by Western blot analysis. Both the Flag-tagged POLGA WT and PEO mutants were used in import assays as described previously (Hussain et al, 2021). For the first reaction involving both POLGA WT and PEO mutants, the assay was set up in parallel with mitochondria isolated from HEK293T cells. The import assay was carried out with 4 µL of the in vitro transcribed and translated protein for both POLGA WT and PEO mutants with 200 µg of isolated mitochondria in import buffer (0.25 M sucrose, 1.5 mM MgCl_2_, 2.5 mg/mL BSA and 10 mM HEPES, pH 7.2) supplemented with 2 mM ATP, 5 mM magnesium acetate, 20 mM KCl and 2 mM sodium succinate. The reaction was initiated at 37 °C by ATP and carried out for an hour. For the second reaction, the above import assay was carried out for both POLGA WT and PEO mutants, and after that, the mitochondria were treated with trypsin (20 µg/ml) on ice for 25 min to remove the outer membrane proteins. Subsequently, the trypsin treatment was inhibited by trypsin inhibitor (50 µg/mL), and the mitochondria were isolated by passing them through a sucrose cushion (0.8 M sucrose in 10 mM HEPES, pH 7.2) and centrifuging at 13,000 rpm at 4 °C for 8 min. For the third reaction, the import assay was carried out, and then mitochondria were resuspended in hypotonic buffer (20 mM KCl and 10 mM HEPES, pH 7.2) and incubated on ice for 20 min, after which the mitoplasts were isolated by centrifuging at 13,000 rpm, 4 °C for 15 min and passed through a sucrose cushion to remove outer membrane proteins. In the fourth reaction, after isolating mitoplasts for both WT and PEO mutants, the mitochondrial matrix was isolated by resuspending the mitoplasts in a hypotonic buffer (5 mM Tris-HCl, pH 7.4 and 1 mM EDTA) for 20 min on ice and sonicated mildly (2 pulses, 15 s each, with 30 s off between cycles). The sonicated samples were ultracentrifuged at 40,000 rpm at 4 °C for 1 h. The pellet constituted the proteins of the inner mitochondrial membrane, and the supernatant contained the matrix fraction. The supernatant was precipitated using 0.6 M Trichloroacetic acid (1 volume of TCA to 4 volumes of supernatant) to concentrate the matrix. In the fifth reaction, a negative control for the assay was set up for both POLGA WT and PEO mutants using CCCP (20 µM) to dissipate the membrane potential. After that, the mitochondria were trypsinised (20 µg/mL) and incubated on ice for 25 min, following which the trypsin treatment was inhibited using trypsin inhibitor (50 µg/mL) and passed through a sucrose cushion (0.8 M sucrose in 10 mM HEPES, pH 7.2) by centrifugation at 13,000 rpm at 4 °C for 8 min to remove trypsin-lysed proteins. For the next five reactions for both POLGA WT and PEO mutants, the import assay was carried out as described above, along with the addition of 2 µM of C4 compound. All the reactions were analysed using western blotting.

### Primer extension assay

Primer extension assays were carried out according to published (Achanta et al, 2005; Gupta et al, 2014). The primer-template pair (Table EV3) comprised the first 40 nucleotides of the mtDNA replication origin. For substrate annealing, a [γ-32P] ATP radiolabelled 20-mer primer was annealed to the 40-mer template. The annealed substrate (0.0375 μM) was incubated at 37 °C for 20 min with indicated amounts of POLGA WT/PEO 2 and POLGB in a buffer containing 10 mM Tris-HCl, pH 7.4, 8 mM MgCl_2_, 0.1 mM DTT, 100 μg BSA/mL, and 50 μM each of dCTP, dGTP, dATP, and dTTP. For rescue experiments, 2 μM of the C1/C4 small molecule was added to the reaction mixture. The products were analysed in a 15% acrylamide-7M urea gel. Primer extension was visualised by the polymerisation of a [γ-32P] labelled 20 bp oligo, which resulted in 40 bp product formation due to the enzymatic activity of mitochondrial DNA polymerase. The exposure was captured using a phosphorimager. Quantification was done using FIJI software.

### Mitochondrial isolation

Mitochondria were isolated from HEK293T and HCT116 POLGA/PEO-YFP-HA cell lines as described (Hussain et al, 2021). Cells were seeded into 15 cm culture dishes at a 4 × 10^5^ cells seeding density for 24 h, and once they reached 80–90% confluency, cells were rinsed thrice with 1X PBS. Cells were then suspended in a mitochondria isolation buffer [20 mM HEPES (pH 7.5), 1.5 mM MgCl_₂_, 1 mM EDTA, 1 mM EGTA, 210 mM sucrose and 70 mM mannitol]. This cell suspension was homogenised using a Dounce homogeniser for 60 to 70 strokes. The cell homogenate was centrifuged at 1000×*g* for 5 min at 4 °C to remove nuclei, followed by a second centrifugation at 10,000×*g* for 15 min at 4 °C. The mitochondrial pellet obtained was washed twice and then resuspended in 200 µL of resuspension buffer (250 mM sucrose, 5 mM magnesium acetate, 40 mM potassium acetate, 10 mM sodium succinate, 1 mM DTT and 20 mM Hepes) and stored at −80 °C till further use.

### Long-range mtDNA amplification assay

Integrity of mtDNA in different cell types was analysed using a Long-range mtDNA amplification assay as described (Hussain et al, 2021). Cells were seeded into 15 cm culture dishes at a 4 × 10^5^ cells seeding density for 24 h; mitochondria were isolated using the above-mentioned protocol, and mitochondrial DNA was isolated using the Mitochondrial DNA isolation kit (BioVision). About 25 ng of mtDNA template was used to amplify full-length mtDNA and mtND1 (as a control) using Phusion high-fidelity DNA polymerase. PCR conditions used were as follows: initial denaturation at 98 °C for 30 s, (i) denaturation at 98 °C for 15 s, (ii) annealing at 65 °C for 30 s, followed by (iii) extension at 72 °C for 15 min. Steps (i)-(iii) were repeated for 24 cycles. A final extension step at 72 °C for 10 min was performed. The PCR products were separated by electrophoresis on an ethidium bromide (EtBr) stained 0.8% agarose gel.

### Slot blot and southwestern blot analysis

To determine the extent of mtDNA replication, a BrdU incorporation assay was carried out as described (Hussain et al, 2021). HEK293T and HCT116 stable lines expressing POLGA WT and PEO mutants were treated with 10 μM BrdU and incubated for 12–16 h. Post-treatment cells were harvested, and mtDNA was isolated using the mtDNA isolation kit (BioVision), according to the manufacturer’s protocol. For Slot blot analysis, mtDNA (1 μg) was denatured by incubating at 98 °C for 10 min. The denatured mtDNA was transferred onto a nitrocellulose membrane using a slot blot apparatus (Bio-Rad). For Southwestern blotting, 2 μg of the isolated mtDNA was digested with the NheI restriction enzyme and resolved on a 0.8% agarose gel. DNA was transferred onto a nitrocellulose membrane using the VacuGene XL apparatus (GE Healthcare) according to the manufacturer’s protocol, and the membrane was crosslinked with UV light using auto crosslink mode in a Stratalinker UV crosslinker apparatus (using a 254 nm light source). The membrane was then blocked with 100% normal horse serum for 90 min at room temperature and probed using anti-BrdU antibody to examine the extent of BrdU incorporation.

### Aggregation analysis using Dynamic Light Scattering (DLS)

The ability of PEO mutants to form high-order aggregate-like structures was analysed using DLS. Wild-type or mutant GST- and Flag-tagged POLGA proteins (750 ng) were equilibrated at 25 °C for 30 s in 1X PBS. The sizes of the proteins were measured using the Zetasizer Nano S (Malvern) using a minimum of 4 analysis cycles (5 × 10 s) at 25 °C, and the sizes of protein molecules were presented as the distribution of size by intensity.

### Tryptophan fluorescence

The changes in the 3D conformation of proteins upon point mutation were analysed using tryptophan fluorescence (Swain et al, 1997). GST- and Flag-tagged wild-type and mutant POLGA (750 ng) were equilibrated in 1X PBS at 25 °C for 30 s. The tryptophan fluorescence was measured using the Fluoromax Plus spectrofluorometer (HORIBA); the samples were excited at 295 nm, and tryptophan emission was recorded from 300 to 450 nm.

### FRET using acceptor photobleaching

Four potential FDA/EMA/PMDA-approved small-molecule disruptors with the lowest EC_50_ values were used to determine their effect on the interaction between CFP-tagged POLGA/PEO mutants (donor) and YFP-tagged MITOL/Tom20 (acceptor) by FRET as described (Karpova et al, 2003). For this purpose, CFP-tagged POLGA/PEO mutants were co-expressed with YFP-tagged MITOL/Tom20. Bleaching was done using 100% power, and bleaching of the acceptor fluorophore was validated using CFP/YFP intensity ratio plots. If interaction occurs between the two proteins, then upon bleaching the acceptor fluorophore, there will be a rescue in the donor fluorescence in the post-bleaching condition. FRET efficiency was calculated based on the before (I_pre_) and post-bleach (I_post_) intensity of the donor fluorophore. Using the formula

$${{{\rm{FRET\; efficiency}}}}\,\left({{{{\rm{E}}}}}_{{{{\rm{FRET}}}}}\right)=\left(1-\left({{{{\rm{I}}}}}_{{{{\rm{pre}}}}}/{{{{\rm{I}}}}}_{{{{\rm{post}}}}}\right)\right.$$

A positive value above 0 and <1 indicates proximity of two fluorophores and energy transfer between donor and acceptor fluorophores because of the interaction between two proteins.

### Mitochondrial respiration assay

For mitochondrial respiration studies using the Seahorse XFe24 Analyser, 4.5 × 10^4^ HCT116 POLGA-YFP-HA, HCT116 PEO 2-YFP HA and HCT116 PEO 6-YFP-HA cells were plated in a Seahorse XF24 Cell Culture plate and incubated for 18 h at 37 °C and 5% CO_2_. The XFe24 Sensor Cartridge was hydrated with ultrapure water overnight. The cartridge was incubated with XF Calibrant solution at 37 °C in a CO_2_-free humidified incubator for 50 min to 1 h before the commencement of the experiment. The cell-containing plate was washed with XF DMEM Assay medium, pH 7.4, supplemented with d-glucose 10 mM, pyruvate 1 mM, and L-glutamine 2 mM at 37 °C according to the manufacturer’s recommended protocol. Injection ports were loaded with the mitochondrial inhibitors to inject a final concentration of 1.5 μM oligomycin, 1 μM FCCP and 0.5 μM rotenone + 0.5 μM Antimycin A. The Oxygen Consumption Rate (OCR) was measured using a Seahorse XFe24 analyser (Agilent Technologies) and normalised to the total cell number. The analysis was done using Wave software.

### Semi-denaturing detergent agarose gel electrophoresis (SDD-AGE)

Whole-cell extracts from HEK293T cells transiently expressing Flag-tagged POLGA WT, PEO mutants and mice tissue lysates were analysed by semi-denaturing detergent agarose gel electrophoresis (SDD-AGE) as described (Halfmann and Lindquist, 2008). Fresh whole-cell or tissue extracts (50 μg) were supplemented with one-third volume of 4X loading dye (20% glycerol, 8% SDS and bromophenol blue in 2X TAE buffer (40 mM Tris, 20 mM acetic acid and 0.4 mM EDTA). Samples were incubated for 7 min at room temperature and resolved on a 1.8% agarose gel in 1X TAE (with 0.1% SDS). Electrophoresis was performed at 60 V for 200 min at 4 °C. Proteins were transferred onto a nitrocellulose membrane using capillary transfer carried out overnight at room temperature in a transfer buffer containing 1X TBS (137 mM NaCl, 2.7 mM KCl and 19 mM Tris-base). Membranes were blocked with 5% skim milk in 1X TBST (137 mM NaCl, 2.7 mM KCl, 19 mM Tris-base and 0.1% Tween 20), followed by immunoblotting with anti-Flag and anti-POLGA antibodies.

### Small molecule screening using the split-GFP complementation system

Prestwick library of FDA/EMA/PMDA-approved small molecules was screened using a split GFP complementation assay (Chun et al, 2007). About 1.5 μg of POLGA-GFP11 or PEO 2 (A467T)-GFP11 was overexpressed along with 1.5 μg of GFP (1–10) plasmid in HEK293T cells. If aggregation is absent, then GFP11 will be available for complementation with GFP (1–10), and GFP fluorescence will be seen. However, if there is aggregation, then GFP11 will not be available for complementation with GFP1-10, and fluorescence intensity will be decreased. Cells overexpressing both pairs were individually treated with each small molecule (100 μM final concentration) in the Prestwick library for 6 h, and GFP fluorescence (excitation at 488 nm/emission at 520 nm) was measured using a Varioskan Flash (Thermo Fisher Scientific) multimode plate reader.

### MTT assay

To analyse the cytotoxic effects of small molecules, HEK293T cells were plated at a seeding density of 1 × 10^4^ cells in a 96-well plate overnight, and the cell viability was determined using the 3-(4,5-dimethylthiazol-2-yl)-2,5-diphenyltetrazolium bromide (MTT) assay. The cells were treated with a concentration gradient (1, 10 and 100 µM) of the small molecule for 6 h, with camptothecin (5 µM, 6 h) treatment being used as a positive control. After incubation, cells were treated with the MTT solution (final concentration, 1 mg/mL) for 2 h. The purple formazan crystals formed in the cells were dissolved with 100 µL DMSO, and the absorbance at 570 nm was recorded using a plate reader. Results were plotted as the percentage of cell viability obtained in the treated cells, assuming that the absorbance of control cells was 100%.

### Biotinylated small molecule immunoprecipitation and interaction studies

C4 small molecule was labelled with biotin using HOOK™-Biotin-PEG2-Amine Labelling Kit (G-Biosciences), following the manufacturer’s protocol. For anti-Biotin tag immunoprecipitations, 700 to 1000 μg of lysates expressing the respective proteins were incubated with 10–15 μL biotinylated C4 compound for 12–16 h. Using streptavidin beads (5 to 7 μL), the protein-small molecule complex was immunoprecipitated for 4 h at 4 °C, after which the complexes were washed thrice with the respective lysis buffers at 4 °C. To determine the interacting proteins, the immunoprecipitates were resolved on SDS-PAGE and probed with the respective antibodies.

### mtDNA copy number

HCT116 stable cells expressing POLGA WT and PEO 2 mutant were seeded in 60 mm dishes, and once they reached 70% confluency, cells were treated with vehicle control or 1 µM of C1 or C4 compound. After 6 h of treatment, cells were harvested, and total DNA was extracted using the QiAmp DNA mini kit according to the manufacturer’s protocol. Quantitative PCR analysis was carried out to check mtDNA copy number using β-globin as the nuclear gene and cytochrome b as the mitochondrial gene, using the following cycling parameters: 95 °C for 7 min; 40 cycles of 95 °C for 10 s, 60 °C for 30 s on the QuantStudio™ 3 Real-Time PCR System (Applied Biosystems, USA). The mtDNA copy number was analysed as mentioned in (Maggo et al, 2024).

### Mice

All the mice studies were carried out at the National Institute of Immunology according to approved animal ethics protocols (IAEC DS/2024/203; IAEC DS/2025-63/032). FVB *POLG*^*A449T/A449T*^ (Homozygous), POLG^A*449/A449T*^ (Heterozygous) and wild-type mice were used as controls (Silva-Pinheiro et al, 2021). All mice were housed in a pathogen-free environment and had free access to water and food. The mice were treated with C1 and C4 compounds every 5th day for four treatment cycles and sacrificed by cervical dislocation at 4, 7, and 20 weeks of age for subsequent analysis.

### Sex as a biological variant in mice studies

Both male and female mice were used in this study in equal proportions. Therefore, in this study, sex was not considered sex as a biological variable.

### Mice genomic DNA isolation and mice genotyping

For genomic DNA isolation, 0.4–0.6 mm mice tail was excised and incubated with DNA digestion buffer (50 mM Tris-HCl, pH 8.0, 100 mM EDTA, pH 8.0, 100 mM NaCl and 1% SDS) and proteinase K at a final concentration of 0.5 mg/mL as described (Wang and Storm, 2006). The mixtures were then incubated overnight at 55 °C with gentle shaking till the tails were completely disrupted. Genomic DNA was precipitated with chilled 100% ethanol, followed by centrifugation at maximum speed for 30 min. Next, the pellet was washed twice with 70% ethanol and air-dried. Finally, the pellet was resuspended in 300 µL of TE buffer. 20 ng of genomic DNA was used for mice genotyping PCR.

Genomic DNA was isolated from the tail of *POLG*^*A449T/A449T*^ (Homo), *POLG*^*A449/A449T*^ (Hetero) and wild-type mice. The genotyping was done using PCR followed by restriction digestion with the PvuII enzyme as described earlier (Silva-Pinheiro et al, 2021). PCR was carried out using Taq DNA polymerase (GeNei). The PCR conditions used are as follows: initial denaturation at 95 °C for 3 min, (i) denaturation at 95 °C for 30 s, (ii) annealing at 64 °C for 30 s, followed by (iii) extension at 72 °C for 1 min. Steps (i–iii) were repeated for 35 cycles. A final extension step at 72 °C for 10 min was performed, followed by digestion with the PvuII restriction enzyme. The wild-type allele produced a fragment of 769 bp, which is cleaved in the A449T allele, producing two fragments of 490 bp + 279 bp.

### mtDNA isolation from mice tissue and Slot blot analysis

FVB mice were injected with 70 mg/kg body weight of BrdU every other day for a total of three injections. On the 6th day, the mice were sacrificed, and mitochondrial DNA (mtDNA) was isolated as described (Kauppila et al, 2018). About 500 mg of vastus lateralis and vastus intermedius muscle tissue was cut into small pieces on ice and washed with chilled 1X PBS. The mice tissue was homogenised for 60 strokes in Mito buffer (320 mM sucrose, 20 mM Tris-HCl, 1 mM EGTA, 0.2% BSA and pH 7.2) using a Dounce homogeniser, and using differential centrifugation, different subcellular fractions were separated, and mitochondria were purified. The mitochondrial pellet was resuspended in 500 μL Mito-DNase buffer (300 mM sucrose, 10 mM MgCl_2_, 20 mM Tris-HCl, pH 7.5, 0.25% BSA, 0.03 mg/mL DNase I type IV and 170 ng/μL RNase A) and incubated at 37 °C for 1 h to digest nuclear DNA and RNA. Post-incubation, mitochondria were re-pelleted at 13,000×g for 20 min at 4 °C and washed with 500 μL Mito buffer. Mitochondria were lysed with 500 μL lysis buffer (20 mM Tris-HCl, 150 mM NaCl, 20 mM EDTA, 1% SDS, pH 8.75, 0.2 mg/ml Proteinase K, 0.2 mg/ml RNase A) at 56 °C for 3–12 h. 100 μL of 6 M Potassium acetate was then added, and DNA was mixed with 500 μL chloroform: isoamyl alcohol (24:1). The aqueous phase fraction was separated at 16,000×*g* for 20 min, 200 mg of RNase A was added to the aqueous phase, and samples were incubated at 37 °C for 1 h to digest the RNA. mtDNA was ethanol precipitated, and the DNA pellet was resuspended in 50 μL TE buffer. The extent of BrdU incorporation was then analysed by the Slot blot as described.

### Assessment of motor activities in mice

#### Grip strength measurements

The grip strength of the four limbs was measured with a digital grip-strength metre (Orchid Scientific) as described (Ha et al, 2018). Mice were first acclimatised for 5 min and then allowed to grip the metal mesh with all four paws. They were then gently pulled backwards by their tails in the horizontal plane until the grip was broken. The force generated at the moment of release was recorded four times, and the average of four readings was plotted.

#### Rotarod

The accelerated rotarod test (Ugo Basile, Gemonio, Italy, model 47600) was used to assess motor coordination and learning in mice by measuring their ability to stay on and run on an accelerating rod as described (Deacon, 2013). The experiment ended when the mouse either fell off the rod or the mice began to rotate without running. After an initial training session, mice underwent three trials, with a 10-min recovery period between each trial.

#### Analysis

To communicate reproducibility across biological replicates, SuperPlots analysis (Lord et al, 2020) has been done wherever applicable.

## Supplementary information

Table EV1

Table EV2

Table EV3

Peer Review File

Expanded View Figures

## Data Availability

All the raw data are available at BioStudies Submission - S-BSST3264, having the URL: https://www.ebi.ac.uk/biostudies/studies/S-BSST3264?key=2ceaa6e1-e05a-4509-853e-4c31184e7097. The source data of this paper are collected in the following database record: biostudies:S-SCDT-10_1038-S44321-026-00532-3.
