# Supplementary material for: Small molecules rescue pathogenic POLGA aggregation and restore its functions in a mouse model of PEO
Source: EMBO Mol Med. 2026 Oct 6;18(11):4494–528. doi: 10.1038/s44321-026-00532-3 (PMC13748654; doi:10.1038/s44321-026-00532-3)
Supplement: Supplementary file 1 [file 44321_2026_532_MOESM4_ESM.pdf]

EMBO Molecular Medicine

Small molecules rescue pathogenic POLG aggregation and restore its functions in a mouse model of PEO

Aftab Mohammed, Tavleen Kaur, Priyanka Panwar, Samanwita Ghosh, Hanif Ahmad, Sadananda Kumbhakar, Meltem Muftuoglu, Carlo Viscomi, and Sagar Sengupta

Corresponding author: Sagar Sengupta (ssg2@nibmg.ac.in)

Review Timeline:

Revision Received:	18th Oct 25
Editorial Decision:	16th Dec 25
Revision Received:	12th Jun 26
Editorial Decision:	17th Jul 26
Editor's Correspondence:	24th Jul 26
Revision Received:	1st Sep 26
Accepted:	16th Sep 26

Editor: Zeljko Durdevic

Transaction Report:

(Note: With the exception of the correction of typographical or spelling errors that could be a source of ambiguity, letters and reports are not edited. Depending on transfer agreements, referee reports obtained elsewhere may or may not be included in this compilation. Referee reports are anonymous unless the Referee chooses to sign their reports.)

16th Dec 2025

Dear Dr. Sengupta,

Thank you for the submission of your manuscript to EMBO Molecular Medicine, and please accept my apologies for the delay in getting back to you, which is due to the fact that one referee needed more time to complete his/her review. We have now received feedback from the three reviewers who agreed to evaluate your manuscript. As you will see from the reports pasted below, all three referees recognize interest of the study but also raise serious concerns that should be addressed in a major revision. If you would like to discuss further the points raised by the referees, I am available to do so via email or video. Let me know if you are interested in this option.

Further consideration of a revision that addresses reviewers' concerns in full will entail a second round of review. EMBO Molecular Medicine encourages a single round of revision only and therefore, acceptance or rejection of the manuscript will depend on the completeness of your responses included in the next, final version of the manuscript. For this reason, and to save you from any frustrations in the end, I would strongly advise against returning an incomplete revision.

We would welcome the submission of a revised version within three months for further consideration. Please let us know if you require longer to complete the revision.

I look forward to receiving your revised manuscript.

Yours sincerely,

Zeljko Durdevic

Zeljko Durdevic
Senior Editor
EMBO Molecular Medicine

*****
When preparing your revised manuscript, please refer to our guidelines: https://link.springer.com/journal/44321/submission-guidelines#cms-Revised-submissions. We perform an initial quality control of all revised manuscripts before re-review; failure to include requested items will delay the evaluation of your revision.

We require:

1) A .docx formatted version of the manuscript text (including legends for main figures, EV figures and tables). Please make sure that the changes are highlighted to be clearly visible.

2) Individual production quality figure files as .eps, .tif, .jpg (one file per figure). For guidance, download the 'Figure Guide PDF': https://media.springernature.com/original/springer-cms/rest/v1/content/27825798/data/v1.

3) A .docx formatted letter INCLUDING the reviewers' reports and your detailed point-by-point responses to their comments. As part of the EMBO Press transparent editorial process, the point-by-point response is part of the Review Process File (RPF), which will be published alongside your paper.

4) A complete author checklist, which you can download from our author guidelines. Please insert information in the checklist that is also reflected in the manuscript. The completed author checklist will also be part of the RPF.

5) Please note that all corresponding authors are required to supply an ORCID ID for their name upon submission of a revised manuscript.

6) It is mandatory to include a 'Data Availability' section after the Materials and Methods. Before submitting your revision, primary

datasets produced in this study need to be deposited in an appropriate public database, and the accession numbers and database listed under 'Data Availability'. Please remember to provide a reviewer password if the datasets are not yet public.

In case you have no data that requires deposition in a public database, please state so in this section. Note that the Data Availability Section is restricted to new primary data that are part of this study.

7) For data quantification: please specify the name of the statistical test used to generate error bars and P values, the number (n) of independent experiments (specify technical or biological replicates) underlying each data point and the test used to calculate p-values in each figure legend. The figure legends should contain a basic description of n, P and the test applied. Graphs must include a description of the bars and the error bars (s.d., s.e.m.).

8) At EMBO Press we ask authors to provide source data for the main manuscript figures. You will receive a separate email with instructions for providing source data with your revised manuscript, including how to upload and organize the files.

9) Our journal encourages inclusion of *data citations in the reference list* to directly cite datasets that were re-used and obtained from public databases. Data citations in the article text are distinct from normal bibliographical citations and should directly link to the database records from which the data can be accessed. In the main text, data citations are formatted as follows: "Data ref: Smith et al, 2001" or "Data ref: NCBI Sequence Read Archive PRJNA342805, 2017". In the Reference list, data citations must be labeled with "[DATASET]". A data reference must provide the database name, accession number/identifiers and a resolvable link to the landing page from which the data can be accessed at the end of the reference.

10) We replaced Supplementary Information with Expanded View (EV) Figures and Tables that are collapsible/expandable online. A maximum of 5 EV Figures can be typeset. EV Figures should be cited as 'Figure EV1, Figure EV2" etc... in the text and their respective legends should be included in the main text after the legends of regular figures.

- For the figures that you do NOT wish to display as Expanded View figures, they should be bundled together with their legends in a single PDF file called *Appendix*, which should start with a short Table of Content. Appendix figures should be referred to in the main text as: "Appendix Figure S1, Appendix Figure S2" etc.

- Additional Tables/Datasets should be labeled and referred to as Table EV1, Dataset EV1, etc. Legends have to be provided in a separate tab in case of .xls files. Alternatively, the legend can be supplied as a separate text file (README) and zipped together with the Table/Dataset file.

11) The paper explained: EMBO Molecular Medicine articles are accompanied by a summary of the articles to emphasize the major findings in the paper and their medical implications for the non-specialist reader. Please provide a draft summary of your article highlighting

- the medical issue you are addressing,

- the results obtained and

- their clinical impact.

This may be edited to ensure that readers understand the significance and context of the research. Please refer to any of our published articles for an example.

12) Author contributions: You will be asked to provide CRediT (Contributor Role Taxonomy) terms in the submission system. These replace a narrative author contribution section in the manuscript.

13) A Conflict of Interest statement should be provided in the main text.

14) Every published paper includes a 'Synopsis' to further enhance discoverability. Synopses are displayed on the journal webpage and are freely accessible to all readers. They include a short stand first (maximum of 300 characters, including space) as well as 2-5 one-sentences bullet points that summarizes the paper. Please write the bullet points to summarize the key NEW findings. They should be designed to be complementary to the abstract - i.e. not repeat the same text. We encourage inclusion of key acronyms and quantitative information (maximum of 30 words / bullet point). Please use the passive voice. Please attach these in a separate file or send them by email, we will incorporate them accordingly.

Please also suggest a striking image or visual abstract to illustrate your article as a PNG file 550 px wide x 300-600 px high.

15) Include a Reagents and Tools Table as part of the Methods section, which can be downloaded from our author guidelines.

EMBO Molecular Medicine has a "scooping protection" policy, whereby similar findings that are published by others during

review or revision are not a criterion for rejection. Should you decide to submit a revised version, I do ask that you get in touch after three months if you have not completed it, to update us on the status.

***** Reviewer's comments *****

Referee #1 (Comments on Novelty/Model System for Author):

1. Technical Quality:
The experiments are well designed and statistically sound. Data reproducibility and clarity of analysis are satisfactory and support the main conclusions.

2. Novelty:
The discovery that small molecules can dissolve aggregates of PEO-type PolgA mutants and restore mitochondrial function represents a conceptually novel and original therapeutic approach.

3. Medical Impact:
The study has strong translational significance, providing a potential disease-modifying treatment for PEO and related mitochondrial disorders using clinically approved compounds.

4. Adequacy of the Model System:
Both the cellular and PolgA A449T knock-in mouse models are appropriate and convincingly demonstrate the in vivo efficacy of the compounds. It would be valuable to assess whether C4 acts selectively on PEO mutants or more broadly on other aggregation-prone proteins. Ethical use of animals appears compliant and unproblematic.

Referee #1 (Remarks for Author):

Reviewer Comments
This is an excellent and highly original study that provides compelling evidence for the chemical reactivation of pathogenic POLG mutants responsible for progressive external ophthalmoplegia (PEO). By identifying two clinically approved small molecules, diltiazem hydrochloride (C1) and sulbactam (C4), that dissolve mutant PolgA aggregates and restore mitochondrial import and mtDNA replication, the authors present a conceptually novel and translationally significant therapeutic approach. The work is of high scientific quality and clinical relevance, and I strongly support its publication after minor revisions. Below are several specific comments that may help to clarify the mechanism and strengthen the interpretation of the results.
1. Specificity and mechanism of C4 action
The study demonstrates that C4 treatment dissolves aggregates formed by PEO mutants, leading to restoration of mitochondrial localization and mtDNA replication. Based on the data shown in Fig. 2D and related analyses, C4 appears to act directly on mutant PolgA proteins to relieve aggregation. However, in cellular contexts, it remains unclear whether C4 acts specifically on PEO mutants or whether the observed effects might also involve modulation of MITOL activity or MITOL-dependent ubiquitination pathways. Although the authors show that C4 does not directly bind MITOL in vitro, additional data confirming that C4 does not alter MITOL's ubiquitination activity (for example, by assessing ubiquitination of other known MITOL substrates or testing C4 effects in MITOL-deficient cells) would provide stronger support for the proposed direct mechanism and target specificity of C4.
2. Relationship between PEO mutant enzymatic activity and C4 effects
Previous studies (e.g., PMID: 40404629) have reported that PEO mutants such as A467T exhibit significantly reduced polymerase activity. In this study, C4 treatment restores mtDNA replication nearly to wild-type levels (Fig. 3E), even though the catalytic activity of these mutants is known to be compromised. It would be helpful if the authors could further clarify how C4 treatment leads to such a strong recovery-for instance, whether C4 enhances the intrinsic enzymatic activity of PolgA, or instead increases the mitochondrial import and availability of the mutant enzyme to a degree that compensates for its partial loss of activity. If feasible, in vitro polymerase assays with or without C4, or correlation analyses between mitochondrial PolgA abundance and mtDNA copy number, would make the mechanistic interpretation more convincing.
3. Specificity of C4 toward PEO aggregates
The authors show that C4 effectively dissolves PEO mutant aggregates. However, it remains unclear whether this disaggregating effect is specific to PolgA mutants or whether C4 exerts a more general action on other misfolded or insoluble proteins. Demonstrating that C4 does not broadly disrupt unrelated protein aggregates, or explicitly discussing this limitation, would further substantiate the specificity of the compound's effect.
4. It would also be informative to examine whether the observed effects of C1 are specific to diltiazem or if they are shared by other calcium channel blockers. Testing additional compounds within the same pharmacological class could help clarify whether this activity is unique to C1 or represents a broader property of calcium antagonists.

Referee #2 (Comments on Novelty/Model System for Author):

Mouse model system is not a problem. Cell line choice is a problem.

Referee #2 (Remarks for Author):

Mohammed et al. "Small molecules disrupt aggregates formed by PEO mutants and restore mitochondrial functions in vivo." The authors characterized several PEO-associated mutants, particularly focusing on their hyperubiquitylated state that contributes to aggregate formation and impaired mitochondrial matrix entry. This work builds off their previous publication in 2021. They then screen FDA approved chemical libraries to identify and validate small molecules capable of preventing aggregate formation in these mutants. Although the Polg A467T knock-in mouse model does not exhibit a pronounced skeletal muscle phenotype or the hallmark ophthalmoplegia seen in PEO patients, treatment with the identified small molecules successfully ameliorated certain motor deficits in the model, demonstrating potential therapeutic potential in vivo. There are many exciting aspects of this paper, but there are many issues with the data that make it hard for the review to interpret the results.

Major concerns:

1. The authors selectively pick and choose when to provide quantifications for their western blot data. The legends state that there are 3 biological replicates for many of the experiments with blots, so the data is there for analysis. The western blotting data is also not always the best with blown out bands for the loading controls or obvious loss of signals from poor transfer like in Figure 1H or 3G, 3H, etc.
2. Many of the experiments are overexpression experiments in cancer cell lines even though the investigators could have reconstituted their PolgA KO cells since some experiments did this. It is also clear that the transfection efficiency is extremely variable in the study (see Figure 1G, 2H, 3H, EV2A, etc.) so, without quantification, one representative western blot is not enough to accurately assess the data.
3. In Figure 1I, How many experimental replicates were performed? If this was just 1, this is not adequate. Same comment for all the experiments with Peason's coefficient quantifications.
4. Discussion could include additional insight into the conformational changes observed in PEO mutants following small molecule treatment, particularly regarding whether these changes represent a full reversion to the wild-type conformation. Additionally, while polymerization activity appears to be improved post-treatment, it would be relevant to address the status of exonuclease and proofreading functions in the corrected enzymes within the discussion.

Minor comments:

1. Figure EV3B last graph: missing a Y axis.
2. Figure EV4C, missing +ve control description in the legend despite it being in the material and methods.
3. Grammatical issue: In Introduction, "This culminated in their enhanced their". 'Their' is used twice.
4. In Results, describing Figure 1H, "were observed in cells expressing PEO mutants compared to PolgA WT"- It seems true for all except PEO2.
5. In Results, "This effect was also recapitulated both in vitro (Figure EV5B, EV5C) or in vivo (Figure EV5D, EV5E)". Both experiments were conducted in vitro.
6. Figure EV4F legend says loading control is HSP60, but it is not on the panel in the actual figure.
7. Insoluble fractionation for western blots is missing in the materials and methods.
8. Scale bars missing on the insets.

Referee #3 (Remarks for Author):

Thank you for sending me this manuscript to evaluate, and please convey my apologies to the authors for the delay in sharing my review.

The authors had previously shown that some PEO mutants are hyperubiquitylated by the E3 ligase MITOL. Here, they extend the study to additional mutants and demonstrate that these mutants aggregate, likely due to conformational changes, and exhibit reduced mitochondrial import. Using a split-GFP complementation assay, they screened for FDA/EMA/approved compounds and identified Diltiazem and Sulbactam as small molecules capable of disrupting POLG aggregates, restoring their mitochondrial import, mtDNA replication, mitochondrial homeostasis, and motor function in an A467T knock-in mouse model.

The study is technically strong, mechanistically insightful, and of translational relevance. While the conclusions are largely supported by the data, several conceptual and experimental issues should be addressed:

-Causality between aggregation and mitochondrial dysfunction.
The central model proposes that POLG aggregation is the primary pathogenic event leading to impaired mitochondrial import and mtDNA replication. While the data correlate aggregation with dysfunction, definitive causality is not fully established.
Suggested experiments:
-Measure POLG activity of PEO mutants in vitro in the presence and absence of C1/C4 to determine whether rescue is import-dependent or if the compounds act as allosteric activators.
-In cells expressing PEO mutants treated with C1/C4, inhibit mitochondrial import (e.g., CCCP) to test whether rescue is abolished.

Figure 1: Mitophagy readouts (LC3B colocalization) could be strengthened by flux assays using bafilomycin controls.
Figure 2: The screen threshold (>95% disruption) is stringent, but the hit selection rationale should be better justified.
No structure-activity relationship or negative analog control is shown.
Figure 3: What about mtDNA copy number levels?
Reduction in mitophagy markers could reflect improved mitochondrial health; therefore autophagic flux controls should be performed.

Response to reviewers' comments

Referee #1:

General comments:

1. Technical Quality:

The experiments are well designed and statistically sound. Data reproducibility and clarity of analysis are satisfactory and support the main conclusions.

2. Novelty:

The discovery that small molecules can dissolve aggregates of PEO-type PolgA mutants and restore mitochondrial function represents a conceptually novel and original therapeutic approach.

3. Medical Impact:

The study has strong translational significance, providing a potential disease-modifying treatment for PEO and related mitochondrial disorders using clinically approved compounds.

4. Adequacy of the Model System:

Both the cellular and PolgA A449T knock-in mouse models are appropriate and convincingly demonstrate the in vivo efficacy of the compounds. It would be valuable to assess whether C4 acts selectively on PEO mutants or more broadly on other aggregation-prone proteins. Ethical use of animals appears compliant and unproblematic.

Response:

We thank the reviewer for finding the manuscript to have good level of technical quality, novelty, medical impact and justified usage of the model system. We feel that the manuscript has been further strengthened in the revised version courtesy to the comments made by the reviewer.

Specific comments:

This is an excellent and highly original study that provides compelling evidence for the chemical reactivation of pathogenic POLG mutants responsible for progressive external ophthalmoplegia (PEO). By identifying two clinically approved small molecules, diltiazem hydrochloride (C1) and sulbactam (C4), that dissolve mutant PolgA aggregates and restore mitochondrial import and mtDNA replication, the authors present a conceptually novel and translationally significant therapeutic approach. The work is of high scientific quality and clinical relevance, and I strongly support its publication after minor revisions. Below are several specific comments that may help to clarify the mechanism and strengthen the interpretation of the results.

Response:

We thank the reviewer for his/her insightful comments and constructive critiques. Please see below our point-by-point response to the reviewer's comments.

Query #1: Specificity and mechanism of C4 action

The study demonstrates that C4 treatment dissolves aggregates formed by PEO mutants, leading to restoration of mitochondrial localization and mtDNA replication. Based on the data shown in Fig. 2D and related analyses, C4 appears to act directly on mutant PolgA proteins to relieve aggregation. However, in cellular contexts, it remains unclear whether C4 acts specifically on PEO mutants or whether the observed effects might also involve modulation of MITOL activity or MITOL-dependent ubiquitination pathways. Although the authors show that C4 does not directly bind MITOL in vitro, additional data confirming that C4 does not alter MITOL's ubiquitination activity (for example, by assessing ubiquitination of other known MITOL substrates or testing C4 effects in MITOL-deficient cells) would provide stronger support for the proposed direct mechanism and target specificity of C4.

Response:

We appreciate the reviewer's concern. We have performed additional experiments and checked the effect of C4 on the ubiquitylation of a mitochondrial helicase RECQL4, apart from PolgA in HeLa shGFP and HeLa shMITOL in ± C4 conditions. Incidentally we have previously shown that RECQL4 and PolgA are both MITOL substrates (PMIDs 33657094, 37495109). As shown in Figure EV4F (Page 8 in the revised version), C4 treatment did not modulate the E3 ligase activity of MITOL and like PolgA, had no effect on the ubiquitylation of RECQL4.

Query #2:

Relationship between PEO mutant enzymatic activity and C4 effects
Previous studies (e.g., PMID: 40404629) have reported that PEO mutants such as A467T exhibit significantly reduced polymerase activity. In this study, C4 treatment restores mtDNA replication nearly to wild-type levels (Fig. 3E), even though the catalytic activity of these mutants is known to be compromised. It would be helpful if the authors could further clarify how C4 treatment leads to such a strong recovery, for instance, whether C4 enhances the intrinsic enzymatic activity of PolgA, or instead increases the mitochondrial import and availability of the mutant enzyme to a degree that compensates for its partial loss of activity. If feasible, in vitro polymerase assays with or without C4, or correlation analyses between mitochondrial PolgA abundance and mtDNA, would make the mechanistic interpretation more convincing.

Response:

We appreciate his/her concerns and suggestions. Hence we have carried out the following experiments

1. We have performed an *in vitro* primer extension assay in ± C4 and C1 conditions (Figure 3A, 3B, Page 8 in revised version). Both the compounds enhanced the polymerisation function of PEO 2 mutant.

2. We also examined the mitochondrial import of PEO mutants compared to PolgA WT in ± C4 via in vitro import assay (Figure EV3I, Page 7 in revised version). Increased mitochondrial import of the PEO mutant in the matrix was observed following C4 treatment.

3. The mtDNA copy number was also enhanced following C4 treatment in PEO mutant cells (Figure 3I, Page 8 in revised version).

Based on the above results, we hypothesize that the effect of C4 is a combination of enhanced intrinsic activity and increased imports that resulted in rescue in mtDNA replication in PEO mutant expressing cells following the treatment of the small molecule. This has been elaborated in the Discussion section also.

Query #3: Specificity of C4 toward PEO aggregates

The authors show that C4 effectively dissolves PEO mutant aggregates. However, it remains unclear whether this disaggregating effect is specific to PolgA mutants or whether C4 exerts a more general action on other misfolded or insoluble proteins. Demonstrating that C4 does not broadly disrupt unrelated protein aggregates, or explicitly discussing this limitation, would further substantiate the specificity of the compound's effect.

Response:

We appreciate the reviewer's concern. We have performed additional experiments and examined the disaggregation effect of C1 and C4 on α-Synuclein aggregates (Figure EV5I and EV5J, Page 11 in the revised version). C1 and C4 did not alter the number of α-Synuclein aggregates puncta/cell, indicating the specificity of C1 and C4 disaggregation effect on PEO aggregates.

Query #4:

It would also be informative to examine whether the observed effects of C1 are specific to diltiazem or if they are shared by other calcium channel blockers. Testing additional compounds within the same pharmacological class could help clarify whether this activity is unique to C1 or represents a broader property of calcium antagonists.

Response:

We thank the reviewer for this insightful comment. We addressed the raised concern by performing the split GFP-based PEO aggregation disruption experiment with Verapamil (same pharmacological class as Diltiazem i.e. C1), Tazobactam and Clavulanic Acid (same pharmacological class as Sulbactam i.e. C4). C1 and C4 were used in parallel in these assays. Verapamil showed similar efficacy to Diltiazem, whereas Tazobactam and Clavulanic Acid showed decreased efficacy in disrupting PEO aggregates compared to Sulbactam (Figure EV3C in revised manuscript, Page 6). The results highlights the differences between C1 and C4 and also the specificity of these compounds.

Referee #2:

General comments:

Mouse model system is not a problem. Cell line choice is a problem.

The authors characterized several PEO-associated mutants, particularly focusing on their hyperubiquitylated state that contributes to aggregate formation and impaired mitochondrial matrix entry. This work builds off their previous publication in 2021. They then screen FDA approved chemical libraries to identify and validate small molecules capable of preventing aggregate formation in these mutants. Although the Polg A467T knock-in mouse model does not exhibit a pronounced skeletal muscle phenotype or the hallmark ophthalmoplegia seen in PEO patients, treatment with the identified small molecules successfully ameliorated certain motor deficits in the model, demonstrating potential therapeutic potential in vivo. There are many exciting aspects of this paper, but there are many issues with the data that make it hard for the review to interpret the results.

Response:

We thank the reviewer for his/her insightful comments and constructive critiques. Please see below our point-by-point response to the reviewer's comments.

Major comments:

Query #1:

1. The authors selectively pick and choose when to provide quantifications for their western blot data. The legends state that there are 3 biological replicates for many of the experiments with blots, so the data is there for analysis. The western blotting data is also not always the best with blown out bands for the loading controls or obvious loss of signals from poor transfer like in Figure 1H or 3G, 3H, etc.

Response:

We highly appreciate the reviewer's concern. As suggested, we have done the quantification of all the graphs of western blots in an Appendix (Excel) file and also replaced the blown-out bands and saturated western blots.

Query #2:

Many of the experiments are overexpression experiments in cancer cell lines even though the investigators could have reconstituted their PolgA KO cells since some experiments did this. It is also clear that the transfection efficiency is extremely variable in the study (see Figure 1G, 2H, 3H, EV2A, etc.) so, without quantification, one representative western blot is not enough to accurately assess the data.

Response:

We appreciate the reviewer's concern, and as suggested, we have the quantification graphs of all the western blots in the attached Appendix (Excel) file. It may also be noted that a significant amount of work has been carried out on well characterised stable cells expressing PolgWT, PEO 2 and PEO 6 which did not involve transient transfections.

Query #3:

In Figure 1I, How many experimental replicates were performed? If this was just 1, this is not adequate. Same comment for all the experiments with Peason's coefficient quantifications.

Response:

We thank the reviewer for raising this important point, and we apologise for the unclear figure legends in the earlier version. In the revised version we have mentioned the number of biological replicates done for each assay and the number of cells analysed confocal based experiments. All the raw data for each experiment (western quantification, confocal experiments, live cell experiments, library screening etc) has also submitted and link given in the Methods section and also in Table EV5.

4. Discussion could include additional insight into the conformational changes observed in PEO mutants following small molecule treatment, particularly regarding whether these changes represent a full reversion to the wild-type conformation. Additionally, while polymerisation activity appears to be improved post-treatment, it would be relevant to address the status of exonuclease and proofreading functions in the corrected enzymes within the discussion.

Response:

We thank the reviewer for prompting us to strengthen the discussion section. We have revised the discussion section as suggested (Page 26).

Minor comments:

Query #1:

Figure EV3B last graph: missing a Y axis.

Response:

We are sorry for the oversight. We have now updated the y-axis in Figure EV2B (in revised manuscript).

Query #2:

Figure EV4C, missing +ve control description in the legend despite it being in the material and methods.

Response:

Described +ve control in the figure legend (Figure 2C, page 32 in revised manuscript).

Query #3:

Grammatical issue: In Introduction, "This culminated in their enhanced their". 'Their' is used twice.

Response:

Corrected the sentence to "This culminated in their enhanced mitochondrial entry and allowed the reinitiation of mtDNA synthesis" (Page 3 in the revised version). .

Query #4:

In Results, describing Figure 1H, "were observed in cells expressing PEO mutants compared to PolgA WT"- It seems true for all except PEO2.

We thank the reviewer for pointing out the oversight. The revised version of the manuscript has the following sentence: "Further enhanced expression of mitophagy markers (LC3B, BNIP3, Atg12, NDP52, Parkin, and p62) were observed in cells expressing PEO expressing PEO 5, PEO 6 and PEO 7 compared to PolgA WT (Figure 1H) (page 6)".

Query #5:

In Results, "This effect was also recapitulated both in vitro (Figure EV5B, EV5C) or in vivo (Figure EV5D, EV5E)". Both experiments were conducted in vitro.

Response:

We thank the reviewer for pointing out this oversight. In the revised manuscript (Page 8) we have changed the sentence to "This effect was recapitulated using both *in vitro* (Figure EV4B) or *in cellulo* (Figure EV4C)…."

Query #6:

Figure EV4F legend says loading control is HSP60, but it is not on the panel in the actual figure.

Response:

We thank the reviewer for also pointing out this mistake. In the revised version we have removed HSP60 from figure legend (Figure EV3F in revised manuscript).

Query #7:

Insoluble fractionation for western blots is missing in the materials and methods.

Response:

We have now added Soluble and insoluble fractionation protocol in the Method section of the revised manuscript (Page 13).

Query #8:

Scale bars missing on the insets.

Response:

All scale bar has been added to the insets and the values also motioned in the figure legends.

Referee #3:

General comments:

Thank you for sending me this manuscript to evaluate, and please convey my apologies to the authors for the delay in sharing my review.

The authors had previously shown that some PEO mutants are hyperubiquitylated by the E3 ligase MITOL. Here, they extend the study to additional mutants and demonstrate that these mutants aggregate, likely due to conformational changes, and exhibit reduced mitochondrial import. Using a split-GFP complementation assay, they screened for FDA/EMA/approved compounds and identified Diltiazem and Sulbactam as small molecules capable of disrupting POLG aggregates, restoring their mitochondrial import, mtDNA replication, mitochondrial homeostasis, and motor function in an A467T knock-in mouse model.

The study is technically strong, mechanistically insightful, and of translational relevance. While the conclusions are largely supported by the data, several conceptual and experimental issues should be addressed.

Response:

We thank the reviewer for finding the manuscript to be technical strong, mechanistically insightful and having translational relevance. We feel that the manuscript has been further strengthened in the revised version courtesy to the comments made by the reviewer.

Query #1:

-Causality between aggregation and mitochondrial dysfunction.

The central model proposes that POLG aggregation is the primary pathogenic event leading to impaired mitochondrial import and mtDNA replication. While the data correlate aggregation with dysfunction, definitive causality is not fully established.

Suggested experiments:

-Measure POLG activity of PEO mutants in vitro in the presence and absence of C1/C4 to determine whether rescue is import-dependent or if the compounds act as allosteric activators.

Query #1:

-In cells expressing PEO mutants treated with C1/C4, inhibit mitochondrial import (e.g., CCCP) to test whether rescue is abolished.

Response:

We appreciate the suggestion made by the reviewer. Hence we have performed the following experiments to address the raised concerns,

1 As suggested, we performed an *in vitro* primer extension assay in ± C4 and ±C1 conditions (Figure 3A, 3B in revised version, page 8), and both compounds enhanced the intrinsic polymerisation function.

2. We also examined the mitochondrial import of PEO mutants compared to PolgA WT in ± C4 via in vitro import assay (Figure EV3I, page 7 in revised version). Increased mitochondrial import of the PEO mutant in the matrix was observed following C4 treatment.

3. The mtDNA content was also enhanced following C4/C1 treatment in PEO mutant cells (Figure 3I, page 8 in revised version).

Based on the above results, treatment with C1 and C4 leads to conformation change of the mutant PolgA which causes a combination of enhanced increased import of the PEO mutants and restoration of mtDNA replication – leading to increased mtDNA content. This has also been elaborated in the Discussion section.

Query #2:

Figure 1: Mitophagy readouts (LC3B colocalization) could be strengthened by flux assays using bafilomycin controls.

Response:

We appreciate the reviewer's concern, and as suggested, we included the bafilomycin control in a confocal-based LC3B colocalization experiment (Figure 5H and 5I, page 9 in the revised manuscript).

Query #3:

Figure 2: The screen threshold (>95% disruption) is stringent, but the hit selection rationale should be better justified.

Response:

We thank the reviewer for the insightful inquiry and apologise for any ambiguity in our initial description that may have confused. He have now reframed the sentence in Page 6 as follows: "Out of the above nine small molecules, two small molecules (C1 and C4) with the lowest EC_{50} values and having minimal possible effect on mitochondrial homeostasis were chosen for most of the subsequent assays".

Query #4:

No structure-activity relationship or negative analogue control is shown.

Response:

We thank the reviewer for constructive critique. We addressed the raised concern by performing the split GFP-based PEO aggregation disruption experiment with Verapamil (same pharmacological class as Diltiazem i.e. C1), Tazobactam and Clavulanic Acid (same pharmacological class as Sulbactam i.e. C4). C1 and C4 were used in parallel in these assays.

Verapamil showed similar efficacy to Diltiazem, whereas Tazobactam and Clavulanic Acid showed decreased efficacy in disrupting PEO aggregates compared to Sulbactam (Figure EV3C in revised manuscript, Page 6). The results highlights the differences between C1 and C4 and also the specificity of these compounds.

Query #4:

What about mtDNA copy number levels?

Response:

We thank the reviewer for the pertinent question. We examined the mtDNA copy number following C1 and C4 treatment. Treatment with C1, C4 improved the mtDNA copy number in PEO mutant expressing cells, almost comparable to the level of PolgA WT expressing cells (Figure 3I, page 8 in revised manuscript).

Query #5:

Reduction in mitophagy markers could reflect improved mitochondrial health; therefore, autophagic flux controls should be performed.

Response:

We appreciate the reviewer's concern, and as suggested, we included the bafilomycin control in a confocal-based LC3B colocalization experiment following C4 and bafilomycin treatment (Figure 5H and 5I, Page 9 in the revised manuscript). The LC3B levels were upregulated after bafilomycin treatment, highlighting active autophagy flux in cells expressing PEO2 but not in cells expressing PolgA WT. Treatment with C4, restored the mitochondrial health to near wildtype levels.

17th Jul 2026

Dear Dr. Sengupta,

Thank you for the submission of your revised manuscript to EMBO Molecular Medicine and please accept my apologies for the delay in getting back to you. We have received feedback from two of the three reviewers who agreed to re-evaluate your manuscript. As the referee #3 despite several reminders will unfortunately not be able to return his/her report in a timely manner, we evaluated your responses to the referee #3 concerns editorially and concluded that you have addressed all points satisfactorily.

Therefore, I am pleased to inform you that we will be able to accept your manuscript pending the following final amendments:

1) Referee reports: Please address all remaining referees' concerns.
- Please implement referee #1 suggestions regarding more cautious interpretations.
- Referee #2 is highlighting several unresolved issues:
o Point 1 - I compared the western blots (WB) from previous and current versions and checked quantifications and to me it seems that this has been resolved. However, please carefully check all the western blot images, source data and quantifications and make sure that all of them are correct. Please normalize all WB quantifications to loading controls.
o Point 2 - Please make sure that the information about the cell lines in complete and accurate in the Methods section.
o Point 3 - Please extend the discussion by addressing the status of exonuclease and proofreading functions in the corrected enzymes.
o Point 4 - Please correct the figure legend and make sure that the information in the figure legends is accurate.
2) Figures: Please upload EV figures as individual high-resolution files, with their legends in the manuscript text. Please check "Author Guidelines" for more information:
https://www.embopress.org/page/journal/17574684/authorguide#figureformat
https://www.embopress.org/page/journal/17574684/authorguide#expandedview
3) Authors Checklist: Please upload the excel file. https://www.embopress.org/pb-assets/embo-site/EMBO%20Press%20Author%20Checklist-1642513524327.xlsx
4) Please consider revising the title to better highlight the main findings, e.g. "Small molecules rescue pathogenic POLG aggregation and restore mitochondrial function in a mouse model of progressive external ophthalmoplegia".
5) Although accepted in the field, in the abstract please refer to the PEO mutants as PEO-associated POLG mutants for the accurate indexing by the search engines. In the introduction you can introduce PEO mutant term and use it in the rest fo the manuscript.
6) Abstract: I have gone through your text and revised it (see below). Please review it for accuracy and amend as you see fit:

POLG, the catalytic subunit of the mitochondrial DNA polymerase, is essential for mitochondrial DNA (mtDNA) replication and maintenance. Mutations in POLG cause progressive external ophthalmoplegia (PEO), but the underlying pathogenic mechanisms remain unclear. Here, we show that the mitochondrial E3 ligase MITOL hyperubiquitylates PEO-associated POLG mutants at lysine 1060, triggering conformational changes and formation of insoluble aggregates that impair mitochondrial import and consequently compromise mtDNA replication and mitochondrial homeostasis. To identify compounds that restore mutant POLG function, we screened an FDA/EMA/PMDA-approved drug library using a split-GFP assay for POLG aggregation. We identified two clinically approved compounds, diltiazem hydrochloride and sulbactam, that disrupted POLG aggregates, prevented aberrant MITOL recognition, restored mitochondrial import, and rescued mtDNA replication and mitochondrial function. Importantly, treatment with either compound significantly improved motor performance in a knock-in mouse model carrying the disease-relevant A467T-equivalent POLG mutation. These findings establish pathogenic aggregation as a previously underrecognized disease mechanism and demonstrate that disrupting POLG aggregates with repurposed drugs can restore mitochondrial function in vivo, providing a potential therapeutic strategy for POLG-associated mitochondrial disease.

7) In the main manuscript file, please do the following:
- Please address all comments suggested by our data editors listed below:
o Figure legends:
1. Please note that the exact p values are not provided in the legends of figures 1F, J; 2B, C; 3B, D, F, H, I; 4C, E; 5C, E, G, I; 6D, F, H, I, J, K; EV1 C, E; EV2 A, B, D; EV3A, C, J; EV4 A, H; EV5 B, D, E, F, H, J.
- Please correct the order and headings of the sections in the manuscript to: Abstract / Keywords / The Paper Explained / Introduction / Results / Discussion / Methods / Data Availability / Acknowledgements / Disclosure and Competing Interests Statement / References / Figure Legends / Expanded View Figure legends
- Please upload Reagents and Tools Table using our .doc template. More information on how to adhere to the format as well as downloadable templates (.docx) for the Reagents and Tools Table can be found in our author guidelines:
https://www.embopress.org/page/journal/17574684/authorguide#structuredmethods
- Please merge two "Disclosure and competing interests statement" into one.
- Author contributions: Please remove it from the manuscript and specify author contributions in our submission system. CRediT has replaced the traditional author contributions section because it offers a systematic machine-readable author contributions

format that allows for more effective research assessment. You are encouraged to use the free text boxes beneath each contributing author's name to add specific details on the author's contribution. More information is available in our guide to authors:
https://www.embopress.org/page/journal/17574684/authorguide#authorshipguidelines
- In data availability please use the following format to report the accession number of your data:

The datasets produced in this study are available in the following databases:
[data type]: [full name of the resource] [accession number/identifier] ([doi or URL or identifiers.org/DATABASE:ACCESSION])

Please check "Author Guidelines" for more information.
https://www.embopress.org/page/journal/17574684/authorguide#availabilityofpublishedmaterial
8) Funding: Please remove the information from the comments field in our system and add it to the line-by-line list of funders. Please ensure that the information in our system is complete and accurate, as it will be linked in PubMed after publication.
9) Tables: Please upload the EV tables together with their legends as separate files. Also, move the references to the main manuscript file.
10) The Paper Explained: Please provide "The Paper Explained" and add it to the main manuscript text. Please check any of recently published EMBO Mol Med research articles for reference.
11) Synopsis: Every published paper now includes a 'Synopsis' to further enhance discoverability. Synopses are displayed on the journal webpage and are freely accessible to all readers. They include separate synopsis image and synopsis text.
- Synopsis image: Please provide a visual abstract as a high-resolution jpeg file exactly 550 px-wide x 300 pixels high to illustrate your article.
- Synopsis text: Please provide a short standfirst (maximum of 300 characters, including space) as well as 2-5 one sentence bullet points that summarise the paper as a .doc file. Please write the bullet points to summarise the key NEW findings. They should be designed to be complementary to the abstract - i.e. not repeat the same text. We encourage inclusion of key acronyms and quantitative information (maximum of 30 words / bullet point). Please use the passive voice.
- Please check your synopsis text and image before submission with your revised manuscript. Please be aware that in the proof stage minor corrections only are allowed (e.g., typos).
12) As part of the EMBO Publications transparent editorial process (see our Editorial at http://embomolmed.embopress.org/content/2/9/329), EMBO Molecular Medicine will publish online a Review Process File (RPF) to accompany accepted manuscripts. This file will be published in conjunction with your paper and will include the anonymous referee reports, your point-by-point response and all pertinent correspondence relating to the manuscript. Let us know if you want to remove or not any figures from it prior to publication. Please note that the Authors checklist will be published at the end of the RPF.
13) Please provide a point-by-point letter INCLUDING my comments as well as the reviewer's reports and your detailed responses (as Word file).

I look forward to reading a new revised version of your manuscript as soon as possible.

Yours sincerely,

Zeljko Durdevic

Zeljko Durdevic
Senior Editor
EMBO Molecular Medicine

*** Instructions to submit your revised manuscript ***

*** PLEASE NOTE *** As part of the EMBO Publications transparent editorial process initiative (see our Editorial at https://www.embopress.org/doi/pdf/10.1002/emmm.201000094), EMBO Molecular Medicine will publish online a Review Process File to accompany accepted manuscripts.

In the event of acceptance, this file will be published in conjunction with your paper and will include the anonymous referee reports, your point-by-point response and all pertinent correspondence relating to the manuscript. If you do NOT want this file to be published, please inform the editorial office at contact@embomolmed.org.

To submit your manuscript, please follow this link:

*****

When preparing your revised manuscript, please refer to our guidelines: https://link.springer.com/journal/44321/submission-guidelines#cms-Revised-submissions. We perform an initial quality control of all revised manuscripts before re-review; failure to include requested items will delay the evaluation of your revision.

We require:

1) A .docx formatted version of the manuscript text (including legends for main figures, EV figures and tables). Please make sure that the changes are highlighted to be clearly visible.

2) Individual production quality figure files as .eps, .tif, .jpg (one file per figure). For guidance, download the 'Figure Guide PDF': https://media.springernature.com/original/springer-cms/rest/v1/content/27825798/data/v1.

3) A .docx formatted letter INCLUDING the reviewers' reports and your detailed point-by-point responses to their comments. As part of the EMBO Press transparent editorial process, the point-by-point response is part of the Review Process File (RPF), which will be published alongside your paper.

4) A complete author checklist, which you can download from our author guidelines. Please insert information in the checklist that is also reflected in the manuscript. The completed author checklist will also be part of the RPF.

5) Please note that all corresponding authors are required to supply an ORCID ID for their name upon submission of a revised manuscript.

6) It is mandatory to include a 'Data Availability' section after the Materials and Methods. Before submitting your revision, primary datasets produced in this study need to be deposited in an appropriate public database, and the accession numbers and database listed under 'Data Availability'. Please remember to provide a reviewer password if the datasets are not yet public.

In case you have no data that requires deposition in a public database, please state so in this section. Note that the Data Availability Section is restricted to new primary data that are part of this study.

7) For data quantification: please specify the name of the statistical test used to generate error bars and P values, the number (n) of independent experiments (specify technical or biological replicates) underlying each data point and the test used to calculate p-values in each figure legend. The figure legends should contain a basic description of n, P and the test applied. Graphs must include a description of the bars and the error bars (s.d., s.e.m.).

8) At EMBO Press we ask authors to provide source data for the main manuscript figures. You will receive a separate email with instructions for providing source data with your revised manuscript, including how to upload and organize the files.

9) Our journal encourages inclusion of *data citations in the reference list* to directly cite datasets that were re-used and obtained from public databases. Data citations in the article text are distinct from normal bibliographical citations and should directly link to the database records from which the data can be accessed. In the main text, data citations are formatted as follows: "Data ref: Smith et al, 2001" or "Data ref: NCBI Sequence Read Archive PRJNA342805, 2017". In the Reference list, data citations must be labeled with "[DATASET]". A data reference must provide the database name, accession number/identifiers and a resolvable link to the landing page from which the data can be accessed at the end of the reference.

10) We replaced Supplementary Information with Expanded View (EV) Figures and Tables that are collapsible/expandable online. A maximum of 5 EV Figures can be typeset. EV Figures should be cited as 'Figure EV1, Figure EV2" etc... in the text and their respective legends should be included in the main text after the legends of regular figures.

- For the figures that you do NOT wish to display as Expanded View figures, they should be bundled together with their legends in a single PDF file called *Appendix*, which should start with a short Table of Content. Appendix figures should be referred to in the main text as: "Appendix Figure S1, Appendix Figure S2" etc.

- Additional Tables/Datasets should be labeled and referred to as Table EV1, Dataset EV1, etc. Legends have to be provided in a separate tab in case of .xls files. Alternatively, the legend can be supplied as a separate text file (README) and zipped together with the Table/Dataset file.

11) The paper explained: EMBO Molecular Medicine articles are accompanied by a summary of the articles to emphasize the major findings in the paper and their medical implications for the non-specialist reader. Please provide a draft summary of your article highlighting

- the medical issue you are addressing,

- the results obtained and

- their clinical impact.

This may be edited to ensure that readers understand the significance and context of the research. Please refer to any of our published articles for an example.

12) Author contributions: You will be asked to provide CRediT (Contributor Role Taxonomy) terms in the submission system. These replace a narrative author contribution section in the manuscript.

13) A Conflict of Interest statement should be provided in the main text.

14) Every published paper includes a 'Synopsis' to further enhance discoverability. Synopses are displayed on the journal webpage and are freely accessible to all readers. They include a short stand first (maximum of 300 characters, including space) as well as 2-5 one-sentences bullet points that summarizes the paper. Please write the bullet points to summarize the key NEW findings. They should be designed to be complementary to the abstract - i.e. not repeat the same text. We encourage inclusion of key acronyms and quantitative information (maximum of 30 words / bullet point). Please use the passive voice. Please attach these in a separate file or send them by email, we will incorporate them accordingly.

Please also suggest a striking image or visual abstract to illustrate your article as a PNG file 550 px wide x 300 px high.

15) Include a Reagents and Tools Table as part of the Methods section, which can be downloaded from our author guidelines.

EMBO Molecular Medicine has a "scooping protection" policy, whereby similar findings that are published by others during review or revision are not a criterion for rejection. Should you decide to submit a revised version, I do ask that you get in touch after three months if you have not completed it, to update us on the status.

*Additional important information regarding Figures

Each figure should be given in a separate file and should have the following resolution:
Graphs 800-1,200 DPI
Photos 400-800 DPI
Colour (only CMYK) 300-400 DPI"

Figures are not edited by the production team. All lettering should be the same size and style; figure panels should be indicated by capital letters (A, B, C etc). Gridlines are not allowed except for log plots. Figures should be numbered in the order of their appearance in the text with Arabic numerals. Each Figure must have a separate legend and a caption is needed for each panel.

*Additional important information regarding figures and illustrations can be found at
https://media.springernature.com/original/springer-cms/rest/v1/content/27825798/data/v1

***** Reviewer's comments *****

Referee #1 (Comments on Novelty/Model System for Author):

The cellular models and the PolgA A449T knock-in mouse are appropriate for assessing the proposed mechanism and in vivo therapeutic potential. Although the mouse model does not fully reproduce all clinical features of human PEO, it provides meaningful evidence of functional benefit. The animal experiments appear to have been conducted under approved ethical protocols, with no major ethical concerns identified.

Referee #1 (Remarks for Author):

This revised manuscript provides a comprehensive and technically strong analysis of PEO-associated POLG mutants, showing their aggregation, enhanced MITOL-associated hyperubiquitylation, and impaired mitochondrial import. The authors further demonstrate that diltiazem (C1) and sulbactam (C4) improve these abnormalities and rescue mitochondrial and motor phenotypes in cellular and knock-in mouse models.

The revision has substantially strengthened the study through additional biochemical, cellular, and in vivo experiments. Overall, I consider the manuscript suitable for publication, subject to the following minor revisions.

First, the causal interpretation should be moderated. Because C1/C4 also improve intrinsic polymerase activity in vitro, the current data do not establish that aggregate dissolution and improved mitochondrial import are the sole or primary basis for functional rescue. The authors should therefore describe the effects of C1/C4 as potentially involving combined changes in POLG conformation, MITOL interaction, mitochondrial import, and polymerase activity.

Second, the discussion of C4 and MITOL-dependent ubiquitylation should be clarified. The data suggest that C4 does not broadly inhibit MITOL activity, but may selectively reduce mutant POLG hyperubiquitylation by altering mutant POLG conformation and/or its aberrant interaction with MITOL. This distinction should be stated clearly.

Finally, statements regarding clinical translation should be softened. The findings provide promising preclinical support for therapeutic development in PEO, but further studies are needed regarding patient-derived cells, pharmacokinetics, long-term safety, and responsiveness across POLG variants.

These revisions concern interpretation and presentation rather than major additional experiments. Provided that they are addressed, I support publication.

Referee #2 (Comments on Novelty/Model System for Author):

The problem is that upon review, the authors said that they have resolved issues and have not. There are multiple minor and major issues that are still unresolved.

Referee #2 (Remarks for Author):

My review comments are in the ** sections after the reviewers' responses. There are still unresolved issues from the last review and wording saying that they have corrected issues when they have not.
1. The authors selectively pick and choose when to provide quantifications for their western blot data. The legends state that there are 3 biological replicates for many of the experiments with blots, so the data is there for analysis. The western blotting data is also not always the best with blown out bands for the loading controls or obvious loss of signals from poor transfer like in Figure 1H or 3G, 3H, etc.
Response:
We highly appreciate the reviewer's concern. As suggested, we have done the quantification of all the graphs of western blots in an Appendix (Excel) file and also replaced the blown-out bands and saturated western blots.
**They now have quantifications for all the blot figures in Appendix but not all of them are reported in the in the Source Data file. Since, I can't access the older version and some of it still looks smeared/overexposed/etc., I am not entirely sure about the replaced blot pictures. I would assume they have replaced the whole blot, especially where the loading control needed to be changed. 1G, H, 3D, 3F, 3H, 5A, 6B, 6F, EV 3F were not normalized to loading control.
Query #2:
Many of the experiments are overexpression experiments in cancer cell lines even though the investigators could have reconstituted their PolgA KO cells since some experiments did this. It is also clear that the transfection efficiency is extremely variable in the study (see Figure 1G, 2H, 3H, EV2A, etc.) so, without quantification, one representative western blot is not enough to accurately assess the data.
Response:
We appreciate the reviewer's concern, and as suggested, we have the quantification graphs of all the western blots in the attached Appendix (Excel) file. It may also be noted that a significant amount of work has been carried out on well characterised stable cells expressing Polg WT, PEO 2 and PEO 6 which did not involve transient transfections.
**There is no mention in the method that flag tagged HEK293T cell lines are stable cell lines. Additionally, Figure 2H uses transiently expressed flag tagged PEO mutants in stably expressing YFP-HA tagged cell lines.
4. Discussion could include additional insight into the conformational changes observed in PEO mutants following small molecule treatment, particularly regarding whether these changes represent a full reversion to the wild-type conformation. Additionally, while polymerization activity appears to be improved post-treatment, it would be relevant to address the status of exonuclease and proofreading functions in the corrected enzymes within the discussion.
Response:
We thank the reviewer for prompting us to strengthen the discussion section. We have revised the discussion section as suggested (Page 26).
**This still hasn't fully been addressed in the Discussion.
Figure EV4F legend says loading control is HSP60, but it is not on the panel in the actual figure.
Response:

We thank the reviewer for also pointing out this mistake. In the revised version we have removed HSP60 from figure legend (Figure EV3F in revised manuscript).
**The legend still says anti hsp60 was used a loading control.

Dear Dr. Sengupta,

I hope you are doing well.

In the meantime, we have received the report from Reviewer #3, which you will find below. The reviewer raises several serious concerns that require your careful attention. Our decision to accept your manuscript in principle remains unchanged; however, we ask that you address all of Reviewer #3's comments and revise the manuscript accordingly.

In particular, please pay close attention to the concerns regarding the mouse phenotype, which the reviewer considers to be inconsistent with the previously published characterization of the same model.

Best wishes,
Zeljko

--
Zeljko Durdevic, PhD
Senior Editor
EMBO Molecular Medicine
z.durdevic@embomolmed.org

Referee #3 (Remarks for Author):

I thank the authors for their responses and for the substantial new experimental work, in particular the primer extension assay (Fig 3A/B), the mtDNA copy number data (Fig 3I), the analogue comparisons (Fig EV3C), and the α-synuclein specificity control (Fig EV5I/J). However, two of my original concerns remain substantively open, and in re-reading the revised manuscript alongside the published literature I have identified a further issue with the in vivo data that I consider the most serious problem in the paper. I set out the major points in order of importance.

Major points:

i) The reported mouse phenotype is inconsistent with the published characterisation of the same model. This is my principal concern, and it bears on the paper's central translational claim. The Polg A449T/A449T knock-in mouse was generated and characterised in Silva-Pinheiro et al., Nucleic Acids Research 2021;49:5230-5248. That study reports:

- Three-month-old homozygotes showed no gross phenotype relative to WT littermates, including a similar body weight curve and similar rotarod performance. The only motor findings were a 19.6% reduction in treadmill endurance and about 40% reduction in spontaneous rearing.
- One-year-old homozygotes were essentially indistinguishable from three-month-old animals; H&E histology showed no gross abnormality in any tissue; and neither reduced lifespan nor obvious age-related phenotypes emerged out to two years.
- mtDNA copy number in skeletal muscle was reduced, without multiple deletions.
- Importantly, no difference in OXPHOS activities was detected between mutant and WT littermates in liver or skeletal muscle.

The present manuscript reports, in 7-week-old animals (i.e. younger than the timepoint at which the source study found almost nothing):

- An approximately 3-5-fold reduction in basal respiration and ATP production in skeletal muscle mitochondria of both heterozygous and homozygous animals (Fig 6H);
- Significantly reduced rotarod performance and body weight in homozygotes (Fig 6I, 6K);
- Aggregate formation, hyperubiquitylation, respiratory deficit and grip-strength loss in heterozygous animals (Fig 6A-J), in some panels apparently as severe as or more severe than homozygotes.

These are not reconcilable as presented. The heterozygous findings are additionally difficult to square with human genetics, as A467T is the common recessive POLG allele, and heterozygous carriers are usually not clinically affected.
The authors are uniquely well placed to explain this discrepancy, and I would ask them to do so explicitly in the manuscript. If the baseline deficit in these animals is not robust, the rescue reported in Fig 6 is not interpretable, and the in vivo section, which is the paper's principal claim to translational significance, cannot stand.

Required:
a. Direct reconciliation, in the text, of Fig 6 with Silva-Pinheiro et al. 2021, addressing age, sex, background, housing and assay differences.
b. The Seahorse experiments on isolated mitochondria (Fig 6G/H) are not adequately described. The Methods (p. 21) describe only the cell-based XF protocol. For isolated organelles the authors must report the assay buffer the respiratory substrates used, µg

mitochondrial protein per well, and normalization (mitochondrial protein or citrate synthase activit). I would also note that the terminology ("basal respiration", "ATP production") and the shape of the Fig 6G trace, which shows a high pre-ADP baseline with little ADP-induced stimulation, are those of a cell-based rather than an organellar assay.
c. If the defect in replication is as strong as in figure 6F then the standard readouts for a mitochondrial myopathy model should be altered- such as mtDNA copy number OXPHOS complex levels (BN-PAGE or western), and COX/SDH histochemistry with quantification of COX-negative fibres. It is a significant gap that a paper whose thesis is restored mtDNA replication in vivo contains no mouse mtDNA copy number measurement at all.
d. Please note the internal tension between Fig 6F (a modest BrdU incorporation difference, confined to homozygotes) and Fig 6H (a 3-5-fold respiratory collapse in both mutant genotypes). These magnitudes are not mutually consistent.

ii)A causality test (import blockade) was not performed
In my original report I asked that mitochondrial import be inhibited in C1/C4-treated cells to determine whether the rescue is import-dependent or whether the compounds act as allosteric activators. The response letter lists three experiments in reply, none of which is this experiment. The in vitro import assay (Fig EV3I) uses CCCP as an internal negative control for the assay itself; this is a different question.
This experiment has become more important in the revision, not less. The authors now propose (Discussion, p. 26) that rescue reflects "a combination of enhanced intrinsic activity and increased import." A dual mechanism of this kind cannot be supported without an experiment that separates the two contributions. I would ask that the original experiment be performed.
Relatedly, the new primer extension data (Fig 3A/B), which are the sole evidence for direct enzymatic activation, rest on a single mutant at a single compound concentration. If C1 and C4 are direct activators of a purified enzyme, this should be established with a concentration-response series and steady-state kinetic parameters. Also please detail the method used and describe the figure in the legend.

iii)Mechanism
The literature on A467T attributes its pathogenicity principally to impaired interaction with the POLγB accessory subunit, with consequent loss of processivity and susceptibility of the free catalytic subunit to LONP1-mediated degradation. POLγB does not appear anywhere in the aggregation model proposed here. Since the authors' own primer extension assays include POLγB, and since the mouse model was characterised in exactly these terms, the manuscript should address whether the two mechanisms are alternative,

sequential or complementary. I would also note that much of the mechanistic cell biology rests on overexpression of tagged PolgA in HEK293T cells, a system in which aggregation of an overexpressed mitochondrial precursor is a recognised artefact.

iv)Statistics

Two issues apply broadly and materially affect the reported significance levels:

• Throughout the imaging analyses (Figs 1J, 5C, 5E, 5G, 5I, 6D, EV2, EV3J, EV5B/H/J), individual cells or muscle fibres are treated as independent observations (n = 25-80) when they derive from three biological replicates. Cells within a replicate are not independent. These analyses should be repeated using replicate-level means or a hierarchical/nested model.

•Fig 6I-K report measurements taken on the same animals at four sequential timepoints but are analysed by ordinary two-way ANOVA. A repeated-measures or mixed-effects model is required.

Minor points:

-Autophagic flux. The bafilomycin control is welcome, but an LC3B-YFP Pearson coefficient is a colocalisation measure, not a flux measure. Flux should be assessed by LC3-II/LC3-I western {plus minus} bafilomycin, and mitophagy flux specifically by degradation of a mitochondrial cargo. In addition, the response letter states that LC3B rose with bafilomycin in PEO 2 but not in PolgA WT cells, whereas the Results (p. 9) and Fig 5H indicate accumulation in both; these statements should be reconciled.

-mtDNA copy number. Fig 3I reports copy number rising from ~70 to ~155 after a 6-hour treatment (Methods, p. 22). Copy number cannot approximately double within six hours. Please clarify the treatment duration or the analysis.

-The inference (Discussion, p. 27) that restored amplification of the full 16.6 kb mtDNA "indirectly indicates that exonuclease/proofreading activity may also be largely restored" does not follow; long-range PCR reports on template integrity, not fidelity. Given that enhancing a proofreading-impaired polymerase could plausibly increase mutation burden, this is a safety-relevant claim. It should either be supported by mtDNA sequencing/mutation-load data or removed.

-"Hence, C1 and C4 should be primed for usage in clinical trials with respect to PEO patients" (p. 28) is not supported in the absence of PK, dose-ranging, a target-engagement biomarker or safety data, and should be tempered.

Referee #1 (Comments on Novelty/Model System for Author):

The cellular models and the PolgA A449T knock-in mouse are appropriate for assessing the proposed mechanism and in vivo therapeutic potential. Although the mouse model does not fully reproduce all clinical features of human PEO, it provides meaningful evidence of functional benefit. The animal experiments appear to have been conducted under approved ethical protocols, with no major ethical concerns identified.

Response:
We thank the reviewer for the observations made.

Referee #1 (Remarks for Author):

This revised manuscript provides a comprehensive and technically strong analysis of PEO- associated POLG mutants, showing their aggregation, enhanced MITOL-associated hyperubiquitylation, and impaired mitochondrial import. The authors further demonstrate that diltiazem (C1) and sulbactam (C4) improve these abnormalities and rescue mitochondrial and motor phenotypes in cellular and knock-in mouse models.

The revision has substantially strengthened the study through additional biochemical, cellular, and in vivo experiments. Overall, I consider the manuscript suitable for publication, subject to the following minor revisions.

Response:
We thank the reviewer for the comments.

First, the causal interpretation should be moderated. Because C1/C4 also improve intrinsic polymerase activity in vitro, the current data do not establish that aggregate dissolution and improved mitochondrial import are the sole or primary basis for functional rescue. The authors should therefore describe the

effects of C1/C4 as potentially involving combined changes in POLG conformation, MITOL interaction, mitochondrial import, and polymerase activity.

Response:
We appreciate his/her concerns and suggestions. Hence, we have moderated the interpretation in the discussion and now describe the effects of C1/C4 as potentially involving combined changes in POLG conformation, its interaction with MITOL, mitochondrial import and intrinsic polymerase activity.

Second, the discussion of C4 and MITOL-dependent ubiquitylation should be clarified. The data suggest that C4 does not broadly inhibit MITOL activity, but may selectively reduce mutant POLG hyperubiquitylation by altering mutant POLG conformation and/or its aberrant interaction with MITOL. This distinction should be stated clearly.

Response:
We appreciate the reviewer's suggestions. We have clearly stated in the discussion that C4 doesn't inhibit the E3 functions of MITOL, but it alters the conformation of PEO mutants and/or their interaction with MITOL.

Finally, statements regarding clinical translation should be softened. The findings provide promising preclinical support for therapeutic development in PEO, but further studies are needed regarding patient-derived cells, pharmacokinetics, long-term safety, and responsiveness across POLG variants.

Response:
We thank the Reviewer for this important point. We have softened the statements regarding clinical translation and now describe our findings as promising preclinical support for therapeutic development in PEO. We have also acknowledged the need for further studies on pharmacokinetics, long-term safety, and responsiveness across different POLG variants.

These revisions concern interpretation and presentation rather than major additional experiments. Provided that they are addressed, I support publication.

Response:
We thank the reviewer for the comments.

Referee #2 (Comments on Novelty/Model System for Author):
The problem is that upon review, the authors said that they have resolved issues and have not.
There are multiple minor and major issues that are still unresolved.

Referee #2 (Remarks for Author):

My review comments are in the ** sections after the reviewers' responses. There are still unresolved issues from the last review and wording saying that they have corrected issues when they have not.

1. The authors selectively pick and choose when to provide quantifications for their western blot data. The legends state that there are 3 biological replicates for many of the experiments with blots, so the data is there for analysis. The western blotting data is also not always the best with blown out bands for the loading controls or obvious loss of signals from poor transfer like in Figure 1H or 3G, 3H, etc.

Previous Response:
We highly appreciate the reviewer's concern. As suggested, we have done the quantification of all the graphs of western blots in an Appendix (Excel) file and also replaced the blown-out bands and saturated western blots.

**They now have quantifications for all the blot figures in the Appendix but not all of them are reported in the in the Source Data file. Since, I can't access the older version and some of it still looks smeared/overexposed/etc., I am not entirely sure about the replaced blot pictures. I would assume they have replaced the whole blot, especially where the loading control needed to be changed. 1G, H, 3D, 3F, 3H, 5A, 6B, 6F, EV 3F were not normalized to loading control.

Response:
We appreciate the reviewer's concern. As suggested, we have replaced all the previously mentioned blown-out loading controls. The data for all the biological replicates for the quantification Western files have been added to the Source data file, and as suggested, all the Western blots have been normalised to loading controls. For the slot blot and SDD-AGE analyses, however, direct normalisation to the corresponding loading controls is not technically applicable in the same manner. In the slot blot experiments, the representative mtDNA blot and the corresponding loading/reference staining were obtained in parallel rather than from the exact same membrane (technically not possible). Similarly, for SDD-AGE, the representative CBB staining was performed separately and an identical gel was run in parallel and used for the corresponding SDD-AGE analysis. Therefore, direct densitometric normalisation between these separately performed gels/membranes would not provide a technically valid comparison and could potentially be misleading.

Query #2:
Many of the experiments are overexpression experiments in cancer cell lines, even though the investigators could have reconstituted their PolgA KO cells since some experiments did this. It is also clear that the transfection efficiency is extremely variable in the study (see Figure 1G, 2H, 3H, EV2A, etc.), so, without quantification, one representative western blot is not enough to accurately assess the data.
Previous Response:
We appreciate the reviewer's concern, and as suggested, we have the quantification graphs of all the western blots in the attached Appendix (Excel) file. It may also be noted that a significant amount of work has been carried out on well-characterised stable cells expressing Polg WT, PEO 2 and PEO 6, which did not involve transient transfections.

**There is no mention in the method that flag tagged HEK293T cell lines are stable cell lines. Additionally, Figure 2H uses transiently expressed flag tagged PEO mutants in stably expressing YFP-HA tagged cell lines.

Response:
We thank the reviewer for raising this important point. We apologise if the previous response was unclear and may have led to a misunderstanding. Throughout the manuscript, all the experiments done with Flag-tagged variants were performed using transient transfection, and this has now been mentioned in the methods section and also the figure legends for clarity. Regarding variable expression, all the replicates have been quantified and normalised to loading controls.

4. Discussion could include additional insight into the conformational changes observed in PEO mutants following small molecule treatment, particularly regarding whether these changes represent a full reversion to the wild-type conformation. Additionally, while polymerization activity appears to be improved post-treatment, it would be relevant to address the status of exonuclease and proofreading functions in the corrected enzymes within the discussion.
Response:
We thank the reviewer for prompting us to strengthen the discussion section. We have revised the discussion section as suggested (Page 26).

**This still hasn't fully been addressed in the Discussion.

Response:
As suggested, we have revised the discussion section, as suggested.

Figure EV4F legend says loading control is HSP60, but it is not on the panel in the actual figure.

Response:
We thank the reviewer for also pointing out this mistake. In the revised version we have removed HSP60 from figure legend (Figure EV3F in revised manuscript).

**The legend still says anti hsp60 was used as a loading control.

Response:
We thank the reviewer for pointing out this mistake. In the revised manuscript, we have removed HSP60.

Referee #3 (Remarks for Author):

I thank the authors for their responses and for the substantial new experimental work, in particular the primer extension assay (Fig 3A/B), the mtDNA copy number data (Fig 3I), the analogue comparisons (Fig EV3C), and the α-synuclein specificity control (Fig EV5I/J).

However, two of my original concerns remain substantively open, and in re-reading the revised manuscript alongside the published literature I have identified a further issue with the in vivo data that I consider the most serious problem in the paper. I set out the major points in order of importance.

Major points:

i) The reported mouse phenotype is inconsistent with the published characterisation of the same model. This is my principal concern, and it bears on the paper's central translational claim. The Polg A449T/A449T knock-in mouse was generated and characterised in Silva-Pinheiro et al., Nucleic Acids Research 2021;49:5230-5248. That study reports:

•Three-month-old homozygotes showed no gross phenotype relative to WT littermates, including a similar body weight curve and similar rotarod performance. The only motor findings were a 19.6% reduction in treadmill endurance and about 40% reduction in spontaneous rearing.

•One-year-old homozygotes were essentially indistinguishable from three-month-old animals; H&E histology showed no gross abnormality in any tissue; and neither reduced lifespan nor obvious age-related phenotypes emerged out to two years.

•mtDNA copy number in skeletal muscle was reduced, without multiple deletions.

•Importantly, no difference in OXPHOS activities was detected between mutant and WT littermates in liver or skeletal muscle.

The present manuscript reports, in 7-week-old animals (i.e. younger than the timepoint at which the source study found almost nothing):

•An approximately 3-5-fold reduction in basal respiration and ATP production in skeletal muscle mitochondria of both heterozygous and homozygous animals (Fig 6H);

•Significantly reduced rotarod performance and body weight in homozygotes (Fig 6I, 6K);

•Aggregate formation, hyperubiquitylation, respiratory deficit and grip-strength loss in heterozygous animals (Fig 6A-J), in some panels apparently as severe as or more severe than homozygotes.

These are not reconcilable as presented. The heterozygous findings are additionally difficult to square with human genetics, as A467T is the common recessive POLG allele, and heterozygous carriers are usually not clinically affected.
The authors are uniquely well placed to explain this discrepancy, and I would ask them to do so explicitly in the manuscript. If the baseline deficit in these animals is not robust, the rescue reported in Fig 6 is not interpretable, and the in vivo section, which is the paper's principal claim to translational significance, cannot stand.

Required:
Direct reconciliation, in the text, of Fig 6 with Silva-Pinheiro et al. 2021, addressing age, sex, background, housing and assay differences.

Response:

We thank the reviewer for highlighting the apparent discrepancy between our findings and those reported by Silva-Pinheiro et al. (2021). We apologise if this difference was not sufficiently addressed in the original manuscript and would like to clarify this point and it has been reconciled in Fig 6 text.

Silva-Pinheiro et al. reported that Polg A449T/A449T mice did not exhibit significant differences in body weight or rotarod performance compared with WT mice at approximately 3 months of age. In contrast, in our study, we observed significant differences in body weight, grip strength, and rotarod performance in both heterozygous and homozygous A449T mice compared with WT animals at 7 weeks of age.

Several factors may contribute to the apparent discrepancy between the two studies. First, in https://www.ncbi.nlm.nih.gov/books/NBK26471/, the authors states "POLG-related disorders comprise a continuum of overlapping phenotypes that were clinically defined before the molecular basis

was known. POLG-related disorders can therefore be considered an overlapping spectrum of disease presenting from early childhood to late adulthood". The point to be noted is that the disease due to POLG mutated can happen at different ages.

Second, differences in genetic background, sex distribution, housing and husbandry conditions, environmental exposure, and other facility-specific factors may contribute to phenotypic variability between studies. Indeed, multiple independent studies have demonstrated that environmental and facility-related factors can substantially influence mouse phenotypes even when genetically homogeneous animals are used. Jaric et al. (2022) (PMID: 3629766) demonstrated persistent differences in body weight and behavioural phenotypes among genetically homogeneous C57BL/6J mice reared in different animal facilities. Similarly, Kovács and Pearce (2013) (PMID: 23817037) reported changes in body weight, motor coordination and motor learning following transfer of a mouse colony to a different animal facility. These findings highlight the potential contribution of environmental and facility-specific factors to inter-laboratory differences in mouse phenotypes.

Importantly, we do not interpret these findings as evidence that the results of either study are contradictory. Rather, the two studies may reflect differences in the age at phenotyping and experimental/environmental background under which the A449T mice were maintained and tested. We have therefore revised the Discussion to explicitly acknowledge these factors and clarify that our observation of an early motor and body-weight phenotype is consistent with phenotypic variability across experimental settings.

An unexpected finding is that heterozygous animals also show accumulation of aggregates outside mitochondria. This was surprising given the strictly recessive nature of the A467T mutation. However, the accumulation of these aggregates in heterozygous animals may not be sufficient to cause a phenotype, either because it does not interfere with cellular function or because its effects are compensated by the WT allele. In homozygous animals, this compensatory mechanism is absent, potentially leading to the accumulation of aggregates and, ultimately, mtDNA instability. We therefore interpret the A467T/WT phenotype as an early biochemical/mitochondrial vulnerability rather than a dominant disease phenotype. Age, tissue-specific mitochondrial stress and secondary mtDNA abnormalities may further influence phenotypic expression. Importantly, the heterozygous phenotype was not uniformly equivalent to the homozygous phenotype across all endpoints. We have avoided extrapolating the mouse heterozygous phenotype directly to human clinical disease.

b. The Seahorse experiments on isolated mitochondria (Fig 6G/H) are not adequately described. The Methods (p. 21) describe only the cell-based XF protocol. For isolated organelles the authors must report the assay buffer the respiratory substrates used, μg mitochondrial protein per well, and normalization (mitochondrial protein or citrate synthase activity). I would also note that the terminology ("basal respiration", "ATP production") and the shape of the Fig 6G trace, which shows a high pre-ADP baseline with little ADP-induced stimulation, are those of a cell-based rather than an organellar assay.

Response:
We appreciate the reviewer's concern. Because these specific datasets do not meet standard physiological quality controls for the isolated mitochondrial assay, we have removed the Seahorse dataset done with mitochondria isolated from the skeletal muscles of the three mice genotypes (Previously Fig 6G/H) from the revised manuscript.

Please note that this modification does not change or alter our primary conclusions, which remain fully supported by our in vitro and in vivo data regarding C1 and C4 treatment rescuing mtDNA replication and motor function in the PEO mouse model. The text in the results section has been amended accordingly.

c. If the defect in replication is as strong as in figure 6F then the standard readouts for a mitochondrial myopathy model should be altered- such as mtDNA copy number OXPHOS complex levels (BN-PAGE or western), and COX/SDH histochemistry with quantification of COX-negative fibres. It is a significant gap that a paper whose thesis is restored mtDNA replication in vivo contains no mouse mtDNA copy number measurement at all.

Response:
We thank the reviewer for this important comment. The primary objective of this study was to determine whether the PEO aggregates can be disrupted and whether these disaggregated PEO mutants can rescue mtDNA replication in vivo. We therefore directly assessed mtDNA replication in skeletal muscle via a BrdU incorporation assay using slot-blot assay and Immunofluorescence and observed a significant improvement following C4 treatment (Fig. 6F).

We agree that mtDNA copy number, OXPHOS complex abundance, and COX/SDH histochemistry would provide complementary measures of downstream mitochondrial consequences. However, we feel that these assays are beyond the specific scope of the present study.

d. Please note the internal tension between Fig 6F (a modest BrdU incorporation difference, confined to homozygotes) and Fig 6H (a 3-5-fold respiratory collapse in both mutant genotypes). These magnitudes are not mutually consistent.

Response:
We thank the reviewer for highlighting an important point. We agree that the magnitude of the respiratory phenotype (previously shown in Fig. 6H/Fig. 6I) appeared disproportionate to the modest BrdU incorporation phenotype in Fig. 6F. To avoid overinterpretation and because the Seahorse measurements were not essential to the primary objective of this study and did not meet standard physiological quality control, we have removed the Seahorse experiment and the corresponding dataset from the revised manuscript.

ii) A causality test (import blockade) was not performed. In my original report I asked that mitochondrial import be inhibited in C1/C4-treated cells to determine whether the rescue is import-dependent or whether the compounds act as allosteric activators. The response letter lists three experiments in reply, none of which is this experiment. The in vitro import assay (Fig EV3I) uses CCCP as an internal negative control for the assay itself; this is a different question. This experiment has become more important in the revision, not less. The authors now propose (Discussion, p. 26) that rescue reflects "a combination of enhanced intrinsic activity and increased import." A dual mechanism of this kind cannot be supported without an experiment that separates the two contributions. I would ask that the original experiment be performed.
Relatedly, the new primer extension data (Fig 3A/B), which are the sole evidence for direct enzymatic activation, rest on a single mutant at a single compound concentration. If C1 and C4 are direct activators of a purified enzyme, this should be established with a concentration-response series and steady-state kinetic parameters. Also please detail the method used and describe the figure in the legend.

Response:

We thank the reviewer for this important point. We agree that the CCCP control in Fig. EV3I validates the in vitro import assay but does not directly distinguish the relative contributions of mitochondrial import and intrinsic enzymatic activation in cells. Although we did not perform the requested import-blockade experiment, our interpretation of the mtDNA replication rescue is based on two complementary observations: C1/C4 enhance mitochondrial import, and they also increase the activity of the PEO 2 mutant enzyme in the biochemical assay. Thus, we consider the rescue to potentially reflect a combination of increased mitochondrial import and enhanced intrinsic activity of the mutant enzyme, rather than increased import alone.

We also considered that pharmacological blockade of mitochondrial import could itself perturb mitochondrial function and potentially confound interpretation of the cellular rescue phenotype. Nevertheless, we agree that the absence of a direct import-blockade experiment limits our ability to formally distinguish the relative contributions of these two mechanisms.

iii) Mechanism

The literature on A467T attributes its pathogenicity principally to impaired interaction with the POLγB accessory subunit, with consequent loss of processivity and susceptibility of the free catalytic subunit to LONP1-mediated degradation. POLγB does not appear anywhere in the aggregation model proposed here. Since the authors' own primer extension assays include POLγB, and since the mouse model was characterised in exactly these terms, the manuscript should address whether the two mechanisms are alternative, sequential or complementary. I would also note that much of the mechanistic cell biology rests on overexpression of tagged PolgA in HEK293T cells, a system in which aggregation of an overexpressed mitochondrial precursor is a recognised artefact.

Response:

We thank the reviewer for raising this important point. We agree that impaired interaction of A467T-POLG with POLγB, leading to reduced processivity and increased susceptibility to LONP1-mediated degradation, is an established component of the A467T phenotype. We do not view this mechanism as mutually exclusive with POLG aggregation. Rather, they may be sequential and/or complementary consequences of the A467T mutation. Reduced association with POLγB could increase the pool of unassembled/free mutant POLG catalytic subunit, which may in turn increase its susceptibility to misfolding, oligomerisation, and aggregation.

Importantly, our aggregation findings are not based solely on transient overexpression in HEK293T cells. We reproduced the aggregation phenotype in stable cell lines and, importantly, in the A467T animal model. These findings provide independent cellular and in vivo evidence that the aggregation phenotype is associated with the A467T mutation and is unlikely to be explained solely by an overexpression artefact.

iv) Statistics

Two issues apply broadly and materially affect the reported significance levels:

• Throughout the imaging analyses (Figs 1J, 5C, 5E, 5G, 5I, 6D, EV2, EV3J, EV5B/H/J), individual cells or muscle fibres are treated as independent observations (n = 25-80) when they derive from three biological replicates. Cells within a replicate are not independent. These analyses should be repeated using replicate-level means or a hierarchical/nested model.

Response:
We thank reviewers for pointing out this mistake. As suggested, all the immunofluorescence data have been replotted as separate plots using a nested model called Superplots (PMID: 32346721).

•Fig 6I-K report measurements taken on the same animals at four sequential timepoints but are analysed by ordinary two-way ANOVA. A repeated-measures or mixed-effects model is required.

Response:
We thank reviewers for pointing out this mistake. As suggested, the data have been replotted as separate plots using a mixed-effects model.

Minor points:
-Autophagic flux. The bafilomycin control is welcome, but an LC3B-YFP Pearson coefficient is a colocalisation measure, not a flux measure. Flux should be assessed by LC3-II/LC3-I western {plus minus} bafilomycin, and mitophagy flux specifically by degradation of a mitochondrial cargo. In addition, the response letter states that LC3B rose with bafilomycin in PEO 2 but not in PolgA WT cells, whereas the Results (p. 9) and Fig 5H indicate accumulation in both; these statements should be reconciled.

Response:
We appreciate the reviewer's concern. The autophagic flux can also be measured based on relative LC3B signal intensity{plus minus}bafilomycin. Hence, we reanalysed the confocal images and plotted the data as L3B signal intensity. The LC3B signal increased with bafilomycin in both PolgA WT and PEO 2, indicating active flux.

-mtDNA copy number. Fig 3I reports copy number rising from ~70 to ~155 after a 6-hour treatment (Methods, p. 22). Copy number cannot approximately double within six hours. Please clarify the treatment duration or the analysis.

Response
We thank the reviewer for raising this important point. In Fig. 3I, it could be misleading if interpreted as an absolute increase in mtDNA copy number. The values were calculated using the (2^{-Delta Ct}) method, with a nuclear DNA target used as the reference, and therefore represent **relative mtDNA** abundance normalised to nuclear DNA, rather than absolute mtDNA copies per cell.

The increase after 6 hours therefore reflects a change in the relative mtDNA qPCR signal and should not be interpreted as a direct measurement showing that cellular mtDNA copy number increased from approximately 70 to 155 copies. We agree that the original use of the term “mtDNA copy number” was potentially misleading and have revised the manuscript and figure legend accordingly.

Importantly, the 6-hour treatment duration is correct. Because this assay provides a relative rather than absolute measurement, we have also avoided making a direct claim that mtDNA genomes themselves doubled during this period.

-The inference (Discussion, p. 27) that restored amplification of the full 16.6 kb mtDNA "indirectly indicates that exonuclease/proofreading activity may also be largely restored" does not follow; long-range PCR reports on template integrity, not fidelity. Given that enhancing a proofreading-impaired

polymerase could plausibly increase mutation burden, this is a safety- relevant claim. It should either be supported by mtDNA sequencing/mutation-load data or removed.

Response :

We thank the reviewer for raising this important point. As suggested, the discussion section has been revised.

-"Hence, C1 and C4 should be primed for usage in clinical trials with respect to PEO patients" (p. 28) is not supported in the absence of PK, dose-ranging, a target-engagement biomarker or safety data, and should be tempered.

Response :

We thank the reviewer for raising this important point. As suggested, the discussion section has been revised.

16th Sep 2026

Dear Dr. Sengupta,

We are pleased to inform you that your manuscript is accepted for publication and is now being sent to our publisher to be included in the next available issue of EMBO Molecular Medicine.

Your manuscript will be processed for publication by EMBO Press. It will be copy edited and you will receive page proofs prior to publication. Please note that you will be contacted by Springer Nature Author Services to complete licensing and payment information.

You may qualify for financial assistance for your publication charges - either via a Springer Nature fully open access agreement or an EMBO initiative. Check your eligibility: https://link.springer.com/journal/44321/how-to-publish-with-us

Should you be planning a Press Release on your article, please notify the production team via this form in order to coordinate publication and release dates: https://forms.cloud.microsoft/e/eH4A8JVP6g

If you have any questions, please do not hesitate to contact the Editorial Office. Thank you for your contribution to EMBO Molecular Medicine.

Yours sincerely,
Zeljko Durdevic

Zeljko Durdevic
Senior Editor
EMBO Molecular Medicine

>>> Please note that it is EMBO Molecular Medicine policy for the transcript of the editorial process (containing referee reports and your response letter) to be published as an online supplement to each paper. If you do NOT want this, you will need to inform the Editorial Office via email immediately. More information is available here: https://link.springer.com/partners/embo-press/editorial-policies#Peer%20review

EMBO Press Author Checklist

Corresponding Author Name: Sagar Sengupta	
Journal Submitted to: EMBO Molecular Medicine	
Manuscript Number: EMM-2025-22560-V3	

USEFUL LINKS FOR COMPLETING THIS FORM
The EMBO Journal - Author Guidelines
EMBO Reports - Author Guidelines
Molecular Systems Biology - Author Guidelines
EMBO Molecular Medicine - Author Guidelines

Reporting Checklist for Life Science Articles (updated January
This checklist is adapted from Materials Design Analysis Reporting (MDAR) Checklist for Authors. MDAR establishes a minimum set of requirements in transparent reporting in the life sciences (see Statement of Task: 10.31222/osf.io/9sm4x). Please follow the journal's guidelines in preparing your
Please note that a copy of this checklist will be published alongside your article.

Abridged guidelines for figures
1. Data
The data shown in figures should satisfy the following conditions:
- the data were obtained and processed according to the field's best practice and are presented to reflect the results of the experiments in an accurate and unbiased manner.
- ideally, figure panels should include only measurements that are directly comparable to each other and obtained with the same assay.
- plots include clearly labeled error bars for independent experiments and sample sizes. Unless justified, error bars should not be shown for technical
- if n<5, the individual data points from each experiment should be plotted. Any statistical test employed should be justified.
- Source Data should be included to report the data underlying figures according to the guidelines set out in the authorship guidelines on Data

2. Captions
Each figure caption should contain the following information, for each panel where they are relevant:
- a specification of the experimental system investigated (eg cell line, species name).
- the assay(s) and method(s) used to carry out the reported observations and measurements.
- an explicit mention of the biological and chemical entity(ies) that are being measured.
- an explicit mention of the biological and chemical entity(ies) that are altered/varied/perturbed in a controlled manner.
- the exact sample size (n) for each experimental group/condition, given as a number, not a range;
- a description of the sample collection allowing the reader to understand whether the samples represent technical or biological replicates (including how many animals, litters, cultures, etc.).
- a statement of how many times the experiment shown was independently replicated in the laboratory.
- definitions of statistical methods and measures:
  - common tests, such as t-test (please specify whether paired vs. unpaired), simple $\chi 2$ tests, Wilcoxon and Mann-Whitney tests, can be unambiguously identified by name only, but more complex techniques should be described in the methods section;
  - are tests one-sided or two-sided?
  - are there adjustments for multiple comparisons?
  - exact statistical test results, e.g., P values = x but not P values < x;
  - definition of 'center values' as median or average;
  - definition of error bars as s.d. or s.e.m.

Please complete ALL of the questions below.
Select "Not Applicable" only when the requested information is not relevant for your study.

Materials

Newly Created Materials	Information included in the manuscript?	In which section is the information available? (Reagents and Tools Table, Materials and Methods, Figures, Data Availability Section)
New materials and reagents need to be available; do any restrictions apply?	Yes	Available, no restrictions apply.

Antibodies	Information included in the manuscript?	In which section is the information available? (Reagents and Tools Table, Materials and Methods, Figures, Data Availability Section)
For **antibodies** provide the following information:<br>- Commercial antibodies: RRID (if possible) or supplier name, catalogue number and or/clone number<br>- Non-commercial: RRID or citation	Yes	Supplied RRID and also the citations (whereever applicable)

DNA and RNA sequences	Information included in the manuscript?	In which section is the information available? (Reagents and Tools Table, Materials and Methods, Figures, Data Availability Section)
**Short novel DNA or RNA including primers, probes**: provide the sequences.	Yes	Sequences provided

Cell materials	Information included in the manuscript?	In which section is the information available? (Reagents and Tools Table, Materials and Methods, Figures, Data Availability Section)
**Cell lines:** Provide species information, strain. Provide accession number in repository **OR** supplier name, catalog number, clone number, and/**OR** RRID.	Yes	Information provided
**Primary cultures:** Provide species, strain, sex of origin, genetic modification status.	Not Applicable	Not applicable
Report if the cell lines were recently **authenticated** (e.g., by STR profiling) and tested for mycoplasma contamination.	Yes	Tested for mycoplasma containation, negative

Experimental animals	Information included in the manuscript?	In which section is the information available? (Reagents and Tools Table, Materials and Methods, Figures, Data Availability Section)
**Laboratory animals or Model organisms:** Provide species, strain, sex, age, genetic modification status. Provide accession number in repository **OR** supplier name, catalog number, clone number, **OR** RRID.	Yes	In method section
**Animal observed in or captured from the field:** Provide species, sex, and age where possible.	Not Applicable	Not applicable
Please detail **housing and husbandry conditions**.	Not Applicable	Not applicable

Plants and microbes	Information included in the manuscript?	In which section is the information available? (Reagents and Tools Table, Materials and Methods, Figures, Data Availability Section)
**Plants:** provide species and strain, ecotype and cultivar where relevant, unique accession number if available, and source (including location for collected wild specimens).	Not Applicable	Not applicable
**Microbes:** provide species and strain, unique accession number if available, and source.	Not Applicable	Not applicable

Human research participants	Information included in the manuscript?	In which section is the information available? (Reagents and Tools Table, Materials and Methods, Figures, Data Availability Section)
If collected and within the bounds of privacy constraints report on age, sex and gender or ethnicity for all study participants.	Not Applicable	Not applicable

Core facilities	Information included in the manuscript?	In which section is the information available? (Reagents and Tools Table, Materials and Methods, Figures, Data Availability Section)
If your work benefited from core facilities, was their service mentioned in the acknowledgments section?	Not Applicable	Not applicable

Design

Study protocol	Information included in the manuscript?	In which section is the information available? (Reagents and Tools Table, Materials and Methods, Figures, Data Availability Section)
If study protocol has been **pre-registered, provide DOI in the manuscript**. For clinical trials, provide the trial registration number **OR** cite DOI.	Not Applicable	Not applicable
Report the **clinical trial registration number** (at ClinicalTrials.gov or equivalent), where applicable.	Not Applicable	Not applicable

Laboratory protocol	Information included in the manuscript?	In which section is the information available? (Reagents and Tools Table, Materials and Methods, Figures, Data Availability Section)
Provide DOI OR other citation details if **external detailed step-by-step protocols** are available.	Not Applicable	Not applicable

Experimental study design and statistics	Information included in the manuscript?	In which section is the information available? (Reagents and Tools Table, Materials and Methods, Figures, Data Availability Section)
Include a statement about **sample size** estimate even if no statistical methods were used.	Yes	Information provided in every Figure legend and Extended View legends
Were any steps taken to minimize the effects of subjective bias when allocating animals/samples to treatment (e.g. **randomization procedure**)? If yes, have they been described?	Yes	Information provided in the Method section
Include a statement about **blinding** even if no blinding was done.	Not Applicable	No blinding was done
Describe **inclusion/exclusion criteria** if samples or animals were excluded from the analysis. Were the criteria pre-established?<br><br>If sample or data points were omitted from analysis, report if this was due to attrition or intentional exclusion and provide justification.	Not Applicable	Not applicable
For every figure, are **statistical tests** justified as appropriate? Do the data meet the assumptions of the tests (e.g., normal distribution)? Describe any methods used to assess it. Is there an estimate of variation within each group of data? Is the variance similar between the groups that are being statistically compared?	Yes	Information provided in every Figure legend and Extended View legends

Sample definition and in-laboratory replication	Information included in the manuscript?	In which section is the information available? (Reagents and Tools Table, Materials and Methods, Figures, Data Availability Section)
In the figure legends: state number of times the experiment was **replicated** in laboratory.	Yes	Information provided in every Figure legend and Extended View legends
In the figure legends: define whether data describe **technical or biological replicates**.	Yes	Information provided in every Figure legend and Extended View legends

Ethics

Ethics	Information included in the manuscript?	In which section is the information available? (Reagents and Tools Table, Materials and Methods, Figures, Data Availability Section)
Studies involving **human participants**: State details of **authority granting ethics approval** (IRB or equivalent committee(s), provide reference number for approval.	Not Applicable	Not applicable
Studies involving **human participants**: Include a statement confirming that **informed consent** was obtained from all subjects and that the experiments conformed to the principles set out in the WMA Declaration of Helsinki and the Department of Health and Human Services Belmont Report.	Not Applicable	Not applicable
Studies involving **human participants: For publication of patient photos**, include a statement confirming that consent to publish was obtained.	Not Applicable	Not applicable
Studies involving experimental **animals**: State details of **authority granting ethics approval** (IRB or equivalent committee(s), provide reference number for approval. Include a statement of compliance with ethical regulations.	Yes	Information provided in the method section
Studies involving **specimen and field samples:** State if relevant **permits** obtained, provide details of authority approving study; if none were required, explain why.	Not Applicable	Not applicable

Dual Use Research of Concern (DURC)	Information included in the manuscript?	In which section is the information available? (Reagents and Tools Table, Materials and Methods, Figures, Data Availability Section)
Could your study fall under dual use research restrictions? Please check biosecurity documents and list of **select agents and toxins** (CDC): https://www.selectagents.gov/sat/list.htm	Not Applicable	Not applicable
If you used a select agent, is the security level of the lab appropriate and reported in the manuscript?	Not Applicable	Not applicable
If a study is subject to dual use research of concern regulations, is the name of the **authority granting approval and reference number** for the regulatory approval provided in the manuscript?	Not Applicable	Not applicable

Reporting

The MDAR framework recommends adoption of discipline-specific guidelines, established and endorsed through community initiatives. Journals have their own policy about requiring specific guidelines and recommendations to complement MDAR.

Adherence to community standards	Information included in the manuscript?	In which section is the information available? (Reagents and Tools Table, Materials and Methods, Figures, Data Availability Section)
State if relevant guidelines or checklists (e.g., **ICMJE, MIBBI, ARRIVE, PRISMA**) have been followed or provided.	Not Applicable	Not applicable
For **tumor marker prognostic studies,** we recommend that you follow the **REMARK** reporting guidelines (see link list at top right). See author guidelines, under 'Reporting Guidelines'. Please confirm you have followed these guidelines.	Not Applicable	Not applicable
For **phase II and III randomized controlled trials**, please refer to the **CONSORT** flow diagram (see link list at top right) and submit the CONSORT checklist (see link list at top right) with your submission. See author guidelines, under 'Reporting Guidelines'. Please confirm you have submitted this list.	Not Applicable	Not applicable

Data Availability

Data availability	Information included in the manuscript?	In which section is the information available? (Reagents and Tools Table, Materials and Methods, Figures, Data Availability Section)
Have **primary datasets** been deposited according to the journal's guidelines (see 'Data Deposition' section) and the respective accession numbers provided in the Data Availability Section?	Yes	Information provided in the method section
Were **human clinical and genomic datasets** deposited in a public access-controlled repository in accordance to ethical obligations to the patients and to the applicable consent agreement?	Not Applicable	Not applicable
Are **computational models** that are central and integral to a study available without restrictions in a machine-readable form? Were the relevant accession numbers or links provided?	Not Applicable	Not applicable
If publicly available data were reused, provide the respective **data citations in the reference list**.	Not Applicable	Not applicable
