# Supplementary material for: Small molecules rescue pathogenic POLGA aggregation and restore its functions in a mouse model of PEO
Source: EMBO Mol Med. 2026 Oct 6;18(11):4494–528. doi: 10.1038/s44321-026-00532-3 (PMC13748654; doi:10.1038/s44321-026-00532-3)
Supplement: Supplementary file 2 [file 44321_2026_532_MOESM5_ESM.pdf]

Expanded View Figures

Figure EV1. PEO mutants show differential DNA polymerase and exonuclease activity in vitro and form aggregates.

(**A**) Purity of proteins used in assays. Coomassie gel showing the purity of the recombinant His-tagged (left, used for in vitro exonuclease and primer extension assay), Flag-tagged (middle, used for import assay and DLS) and GST-tagged (right, used for tryptophan fluorescence assay and DLS) POLGA WT and PEO mutants. Three independent preparations were used for each set of tagged proteins. (**B**, **C**) PEO 6 shows decreased exonuclease activity. (**B**) In vitro exonuclease assay was carried out with His-tagged POLGA WT, PEO 5, PEO 6 and PEO 7 using a paired template labelled with ATP [γ-^{32}P]. (**C**) Quantitation of (**B**). AU is arbitrary units. $n = 3$ biological replicates. (**D**, **E**) PEO 5 and PEO 6 show minimal primer extension activity. (**D**) In vitro primer extension assay was performed with His-tagged PEO mutants and POLGA WT using an annealed template labelled with ATP [γ-^{32}P]. (**E**) Quantitation of (**D**). $n = 3$ biological replicates. (**F**) PEO mutants undergo enhanced proteasomal degradation. Cell extracts were made from HEK293T cells (in ±MG132 conditions) transiently expressing Flag-tagged POLGA WT, PEO 5, PEO 6 and PEO 7 mutants, along with Myc-tagged MITOL. Western analysis was done with the indicated antibodies. $n = 3$ biological replicates. (**G**) PEO mutants show decreased mitochondrial import. An in vitro import assay was done using Flag-tagged POLGA WT, PEO 5, PEO 6 and PEO 7 mutants. Post-incubation, each of the indicated mitochondrial fractions was subjected to Western blot analysis with the indicated antibodies. n = 3 biological replicates. (**H**) PEO 5, PEO 6 and PEO 7 mutants form high-order structures. Flag-tagged POLGA WT, PEO 5, PEO 6 and PEO 7 proteins were monitored by DLS and represented by an intensity plot. $n = 3$ biological replicates. Data represent the mean ± SD and were analysed using a two-way ANOVA (for **C**, **E**) statistical test with Tukey's (**C**) and Dunnett's (**E**) multiple comparisons tests.

 © The Author(s)

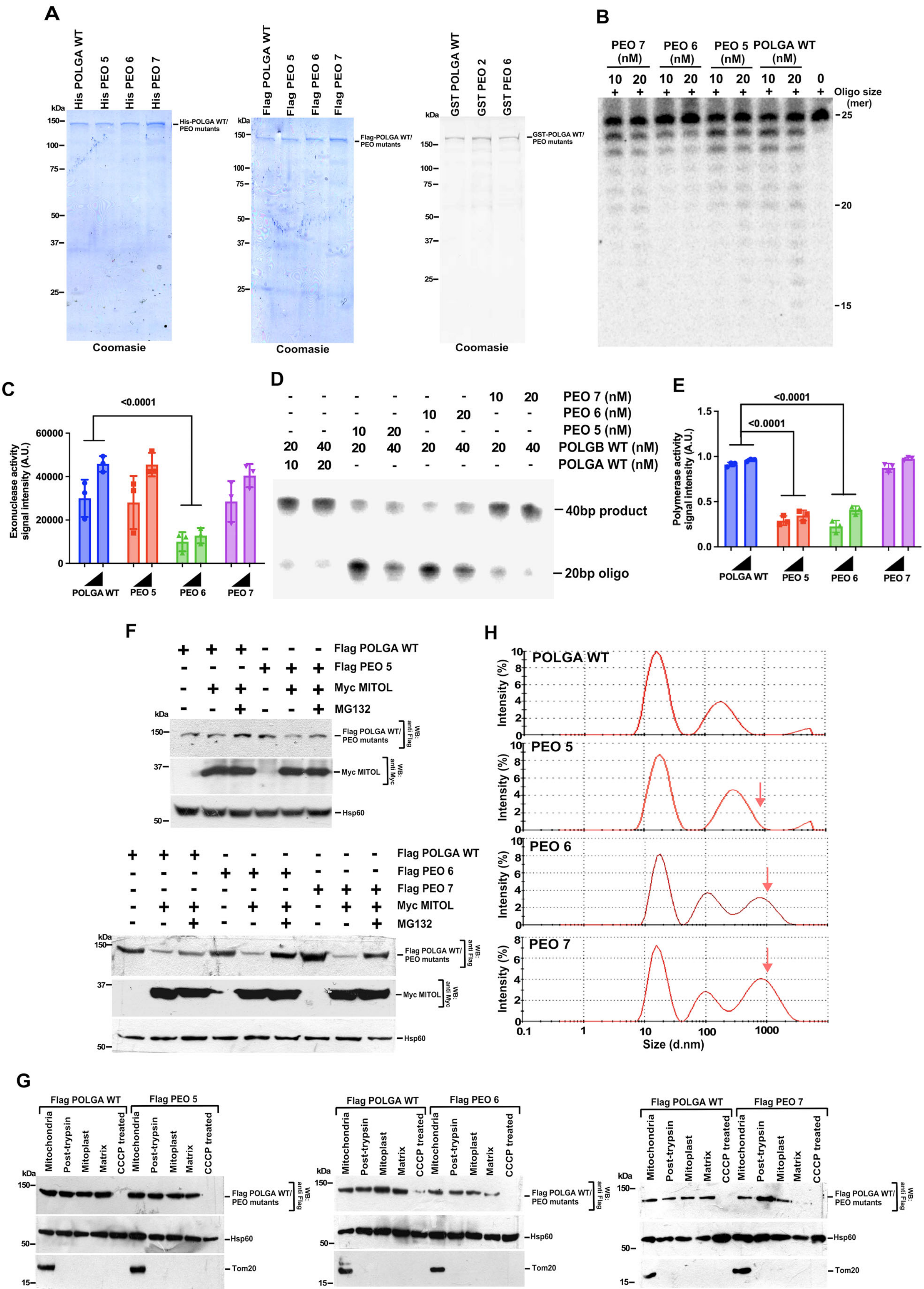
A
His POLGA WT
His PEO 5
His PEO 6
His PEO 7
kDa
150
100
75
50
37
25
His-POLGA WT/ PEO mutants
Coomasie
Flag POLGA WT
Flag PEO 5
Flag PEO 6
Flag PEO 7
Flag-POLGA WT/ PEO mutants
GST POLGA WT
GST PEO 2
GST PEO 6
GST-POLGA WT/ PEO mutants
B
PEO 7 (nM)
PEO 6 (nM)
PEO 5 (nM)
POLGA WT (nM)
10 20 10 20 10 20 10 20 0
Oligo size (mer)
25
20
15
C
<0.0001
Exonuclease activity signal intensity (A.U.)
60000
40000
20000
0
POLGA WT
PEO 5
PEO 6
PEO 7
D
PEO 7 (nM)
PEO 6 (nM)
PEO 5 (nM)
POLGB WT (nM)
POLGA WT (nM)
40bp product
20bp oligo
E
<0.0001
<0.0001
Polymerase activity signal intensity (A.U.)
1.5
1.0
0.5
0.0
POLGA WT
PEO 5
PEO 6
PEO 7
F
Flag POLGA WT
Flag PEO 5
Myc MITOL
MG132
Flag POLGA WT/ PEO mutants
Myc MITOL
Hsp60
WB: anti Flag
WB: anti Myc
Flag PEO 6
Flag PEO 7
G
Flag POLGA WT
Flag PEO 5
Flag PEO 6
Flag PEO 7
Mitochondria
Post-trypsin
Mitoplast
Matrix
CCCP treated
Tom20
H
POLGA WT
PEO 5
PEO 6
PEO 7
Intensity (%)
Size (d.nm)
0.1
1
10
100
1000

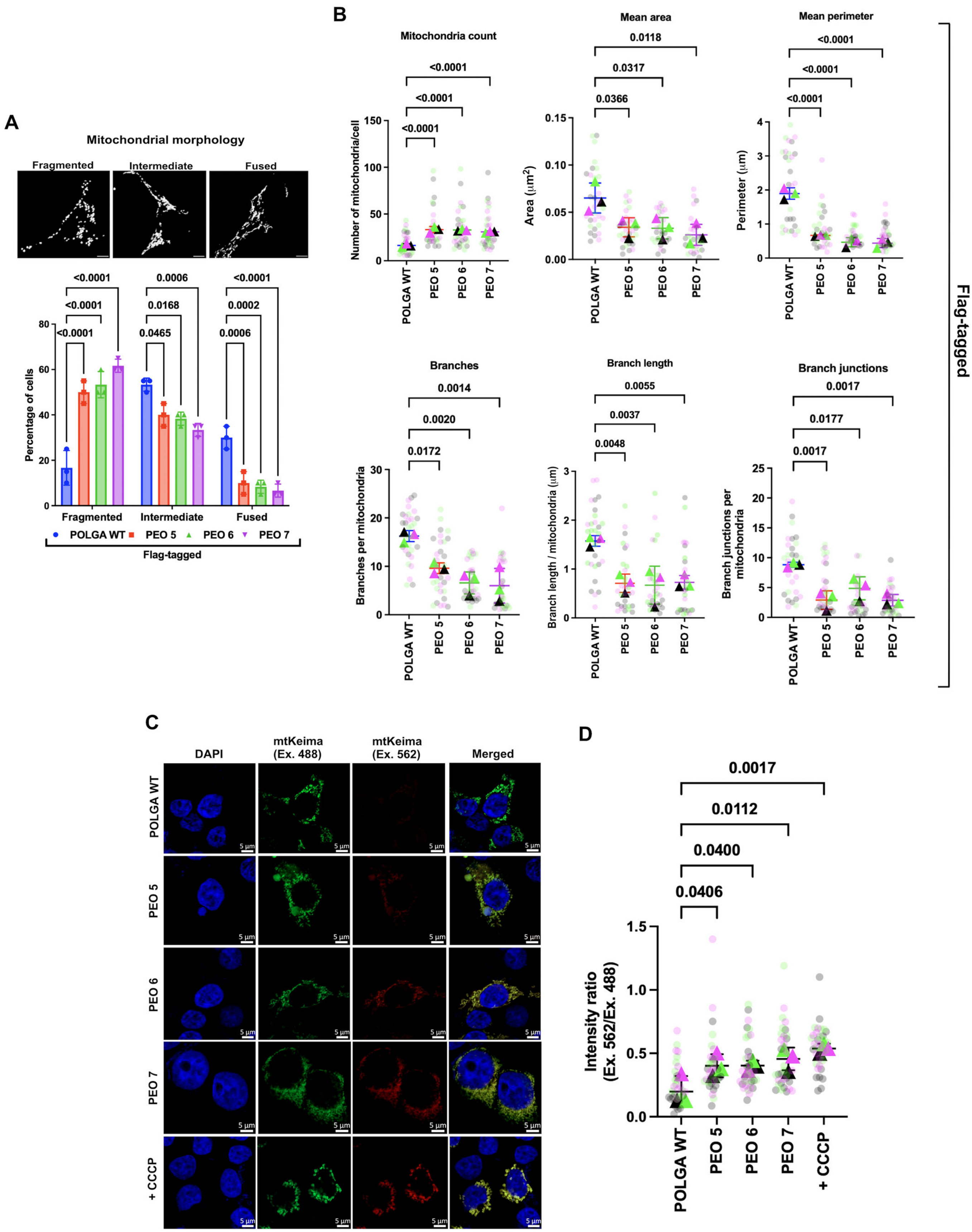
A
Mitochondrial morphology
Fragmented
Intermediate
Fused
<0.0001
<0.0001
<0.0001
0.0006
0.0168
0.0465
<0.0001
0.0002
0.0006
Percentage of cells
Fragmented
Intermediate
Fused
POLGA WT
PEO 5
PEO 6
PEO 7
Flag-tagged
B
Mitochondria count
<0.0001
<0.0001
<0.0001
Number of mitochondria/cell
Mean area
0.0118
0.0317
0.0366
Area (μm²)
Mean perimeter
<0.0001
<0.0001
<0.0001
Perimeter (μm)
Branches
0.0014
0.0020
0.0172
Branches per mitochondria
Branch length
0.0055
0.0037
0.0048
Branch length / mitochondria (μm)
Branch junctions
0.0017
0.0177
0.0017
Branch junctions per mitochondria
POLGA WT
PEO 5
PEO 6
PEO 7
Flag-tagged
C
DAPI
mtKeima (Ex. 488)
mtKeima (Ex. 562)
Merged
POLGA WT
PEO 5
PEO 6
PEO 7
+ CCCP
5 μm
D
0.0017
0.0112
0.0400
0.0406
Intensity ratio (Ex. 562/Ex. 488)
POLGA WT
PEO 5
PEO 6
PEO 7
+ CCCP

 © The Author(s)

Figure EV2. PEO mutant-expressing cells show mitochondrial fission and mitophagy.

(**A**, **B**) Mitochondrial morphology is altered in cells expressing PEO mutants. (**A**) Mitochondrial morphology of HEK293T cells expressing Flag-tagged POLGA WT, PEO 5, PEO 6 and PEO 7 was monitored after staining with Mitotracker CMX ROS using immunofluorescence. (Top) Representative images are shown for fragmented, intermediate and fused mitochondria. (Bottom) Quantitation of fragmented, intermediate and fused mitochondria. (**B**) Parameters indicating mitochondrial fission are increased in cells expressing PEO mutants. Quantitation using SuperPlots was carried out using FIJI software, and the different mitochondrial morphology parameters were calculated using the mitochondrial analyser plugin (FIJI). $n = 30$ cells over three biological replicates. (**C**, **D**) Mitophagy was upregulated in PEO-expressing cells. (**C**) Mitophagy was monitored in HEK293T cells expressing POLGA WT, PEO 5, PEO 6 and PEO 7 using the mtKeima assay. CCCP treatment on the same cells was used as a positive control. The immunofluorescence-based readout was monitored. Scale bar = 5 μm. (**D**) Quantitation of (**C**) using SuperPlots. $n = 35$ cells over three biological replicates. Data represent the mean ± SD and were analysed using ordinary one-way (for **B**, **D**) and two-way ANOVA (for **A**) statistical tests with Tukey's (**A**) and Dunnett's (**B**, **D**) multiple comparisons tests.

© The Author(s)

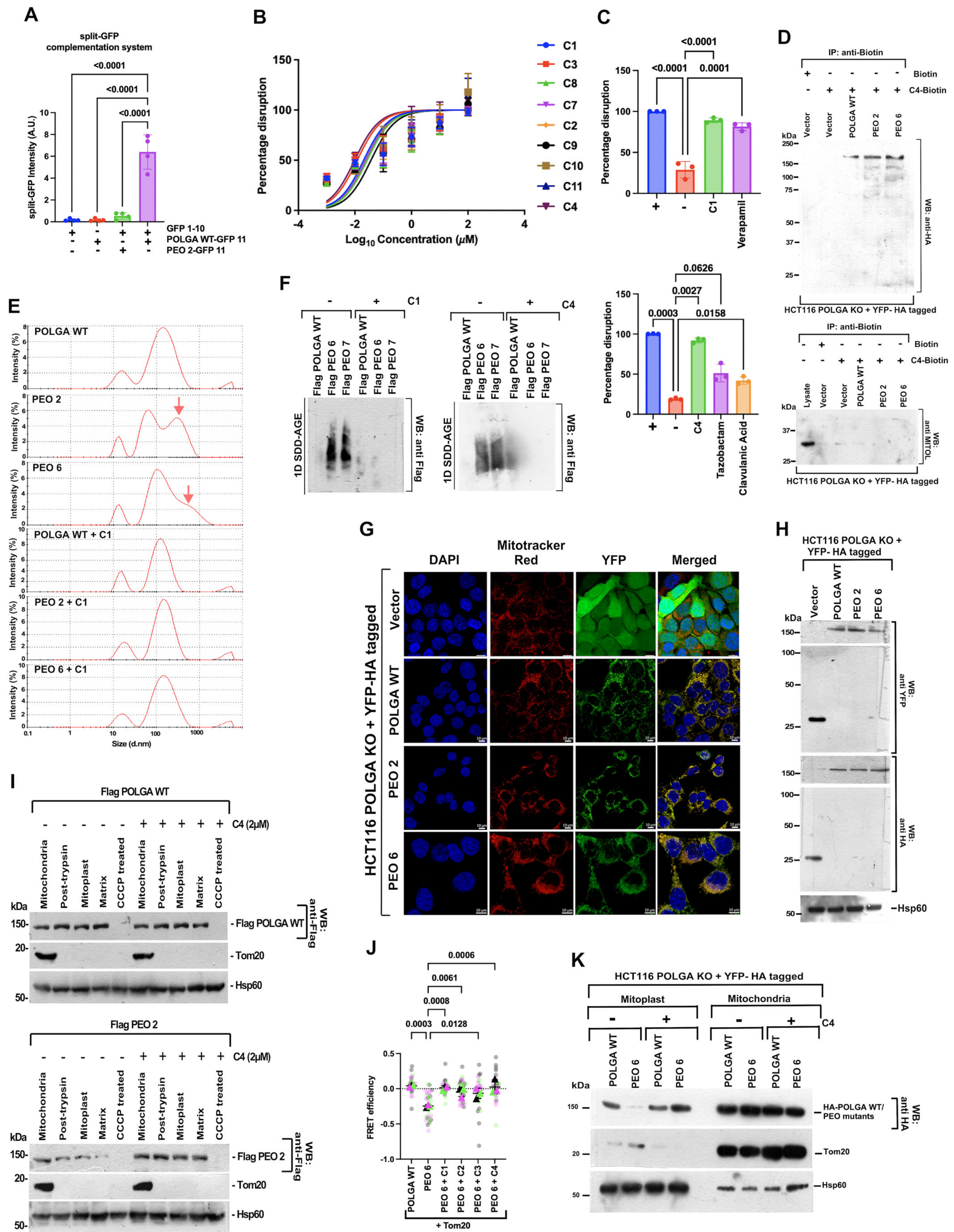
A
split-GFP complementation system
<0.0001
<0.0001
<0.0001
split-GFP Intensity (A.U.)
GFP 1-10
POLGA WT-GFP 11
PEO 2-GFP 11
B
Percentage disruption
Log10 Concentration (μM)
C1
C3
C8
C7
C2
C9
C10
C11
C4
C
<0.0001
<0.0001
0.0001
Percentage disruption
C1
Verapamil
D
IP: anti-Biotin
Biotin
C4-Biotin
kDa
Vector
POLGA WT
PEO 2
PEO 6
WB: anti-HA
HCT116 POLGA KO + YFP- HA tagged
Lysate
WB: anti MITOL
E
POLGA WT
PEO 2
PEO 6
POLGA WT + C1
PEO 2 + C1
PEO 6 + C1
Intensity (%)
Size (d.nm)
F
C1
C4
Flag POLGA WT
Flag PEO 6
Flag PEO 7
1D SDD-AGE
WB: anti Flag
0.0626
0.0027
0.0003
0.0158
Percentage disruption
C4
Tazobactam
Clavulanic Acid
G
DAPI
Mitotracker Red
YFP
Merged
HCT116 POLGA KO + YFP-HA tagged
Vector
POLGA WT
PEO 2
PEO 6
H
HCT116 POLGA KO + YFP- HA tagged
Vector
POLGA WT
PEO 2
PEO 6
kDa
WB: anti YFP
WB: anti HA
Hsp60
I
Flag POLGA WT
C4 (2μM)
Mitochondria
Post-trypsin
Mitoplast
Matrix
CCCP treated
kDa
Flag POLGA WT
Tom20
Hsp60
WB: anti-Flag
Flag PEO 2
Flag PEO 2
J
0.0006
0.0061
0.0008
0.0003
0.0128
FRET efficiency
POLGA WT
PEO 6
PEO 6 + C1
PEO 6 + C2
PEO 6 + C3
PEO 6 + C4
+ Tom20
K
HCT116 POLGA KO + YFP- HA tagged
Mitoplast
Mitochondria
C4
kDa
POLGA WT
PEO 6
HA-POLGA WT/ PEO mutants
Tom20
Hsp60
WB: anti HA

 © The Author(s)

Figure EV3. Treatment of cells expressing PEO mutants with small molecules leads to loss of aggregate formation and restores mitochondrial import.

(**A**) Validation of the split-GFP complementation system for POLGA. The different split-GFP complementation pairs were expressed in HEK293T cells, and GFP fluorescence was measured and plotted as GFP fluorescence intensity. AU arbitrary units. Mean ± SD. $n = 4$ biological replicates. (**B**) Dose-response curve of small molecules that disrupt PEO 2 aggregates. A GFP-based protein complementation assay was carried out in the presence of a range of concentrations of the nine small molecules identified in the protein complementation assay. The concentrations used were 1 nM, 10 nM, 100 nM, 1 µM, 10 µM and 100 µM. $n = 3$ biological replicates. (**C**) Effect of C1 and C4 analogues on the interaction between PEO 2-GFP11 aggregates. Protein complementation assays were carried out with (Top) C1 and its analogue (Verapamil), (Bottom) C4 and its analogues (tazobactam and clavulanic acid). (+) indicates the positive control in which POLGA-GFP11 and GFP (1–10) were co-expressed. (−) indicates the negative control in which PEO 2-GFP11, along with GFP (1–10), was expressed. $n = 3$ biological replicates. (**D**) C4 interacts with POLGA WT and PEO mutants. Immunoprecipitation was performed using streptavidin beads to pull down biotin-labelled C4 compound incubated with the cell extracts of YFP-HA-tagged stable cell lines. The extent of interaction of (Top) POLGA/PEO and (Bottom) MITOL with biotinylated C4 was assessed by probing with the indicated antibodies. $n = 3$ biological replicates. (**E**) C1 treatment decreases aggregate formation by PEO mutants. Aggregation of POLGA WT, PEO 2 and PEO 6 was monitored by DLS (in ±C1 conditions) and represented as an intensity plot. The intensity plots of the proteins without C1 treatment are the same as those in Fig. 2E. $n = 3$ biological replicates. (**F**) Reversion of aggregation occurs after treatment with C1 and C4. Cell lysates were made from HEK293T cells transiently expressing Flag-tagged POLGA WT, PEO 6 and PEO 7 mutants, with and without C1 (Left) and C4 (Right) treatment. 1D SDD-AGE followed by immunoblotting was carried out with an anti-Flag antibody. $n = 3$ biological replicates. (**G**, **H**) YFP-HA-tagged stable cell line validation. HCT116 YFP-HA (termed vector), HCT116 POLGA WT-YFP-HA (termed POLGA WT), HCT116 (A467T)-YFP-HA (termed PEO 2) and HCT116 (L304R)-YFP-HA (termed PEO 6) stable cell lines were generated in HCT116 POLGA CRISPR knockout cells. The stable lines were validated by (**G**) immunofluorescence. Scale bar = 10 µm. (**H**) Western blotting using the indicated antibodies. $n = 3$ biological replicates. (**I**) PEO mutants show increased mitochondrial import after C4 treatment. In vitro import assay was carried out using in vitro transcribed and translated Flag-tagged POLGA WT (Top) and PEO 2 (Bottom) mutants, without and after treatment with C4. Post-incubation, each of the indicated mitochondrial fractions was subjected to Western blot analysis with the indicated antibodies. $n = 3$ biological replicates. (**J**) Identified small molecules enhance the interaction between PEO mutants and Tom20. Graphical representation, using SuperPlots, of FRET efficiency calculated based on the fluorescence intensity of the donor fluorophore for interaction between Tom20 and PEO 6 mutant, without or after treatment with C1, C2, C3 and C4 compounds. POLGA WT has been used as a positive control. $n = 30$ across three biological replicates. (**K**) C4 treatment restores the mitochondrial entry of PEO 6. Mitochondria and mitoplast fractions were isolated from HCT116 cells stably expressing YFP-HA-tagged POLGA WT and PEO 6 protein, grown without or after C4 treatment. (**J**) The relative presence of proteins in the mitoplast and mitochondrial fractions was determined by Western blot analysis using the indicated antibodies. $n = 3$ biological replicates. Data represent the mean ± SD and were analysed using an ordinary one-way (for **A**, **C** and **J**) statistical test with Sidak's (**A**) and Dunnett's (**C**, **J**) multiple comparisons test.

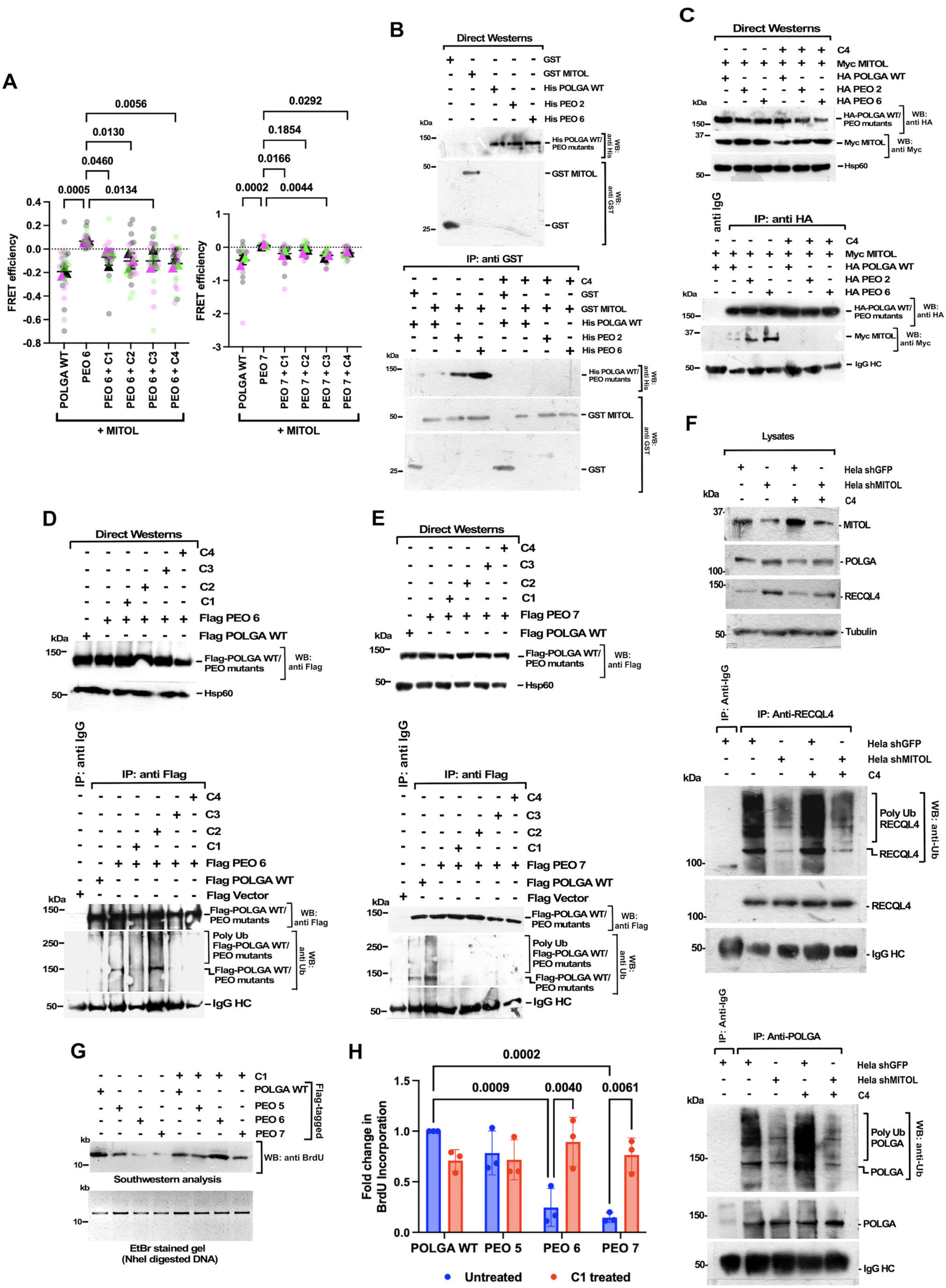
A
0.0056
0.0130
0.0460
0.0005
0.0134
FRET efficiency
POLGA WT
PEO 6
PEO 6 + C1
PEO 6 + C2
PEO 6 + C3
PEO 6 + C4
+ MITOL
0.0292
0.1854
0.0166
0.0002
0.0044
PEO 7
PEO 7 + C1
PEO 7 + C2
PEO 7 + C3
PEO 7 + C4
B
Direct Westerns
GST
GST MITOL
His POLGA WT
His PEO 2
His PEO 6
kDa
His POLGA WT/ PEO mutants
WB: anti His
GST MITOL
WB: anti GST
IP: anti GST
C4
IgG HC
C
Direct Westerns
Myc MITOL
HA POLGA WT
HA PEO 2
HA PEO 6
HA-POLGA WT/ PEO mutants
WB: anti HA
Myc MITOL
WB: anti Myc
Hsp60
anti IgG
IP: anti HA
D
Direct Westerns
C3
C2
C1
Flag PEO 6
Flag POLGA WT
Flag-POLGA WT/ PEO mutants
WB: anti Flag
IP: anti IgG
IP: anti Flag
Flag Vector
Poly Ub Flag-POLGA WT/ PEO mutants
WB: anti Ub
E
Flag PEO 7
F
Lysates
Hela shGFP
Hela shMITOL
MITOL
POLGA
RECQL4
Tubulin
IP: Anti-IgG
IP: Anti-RECQL4
Poly Ub RECQL4
WB: anti-Ub
IP: Anti-POLGA
Poly Ub POLGA
G
POLGA WT
PEO 5
PEO 6
PEO 7
Flag-tagged
kb
WB: anti BrdU
Southwestern analysis
EtBr stained gel (NheI digested DNA)
H
0.0002
0.0009
0.0040
0.0061
Fold change in BrdU Incorporation
Untreated
C1 treated

Figure EV4. Small molecule treatment disrupts the interaction of PEO mutants with MITOL.

(**A**) Identified small molecules disrupt the interaction between PEO mutants and MITOL. Graphical representation, using SuperPlots, of FRET efficiency calculated based on the fluorescence intensity of the donor fluorophore for interaction between (Left) MITOL and PEO 6 mutant or (Right) MITOL and PEO 7 mutant, without or after treatment with C1, C2, C3 and C4 compounds ($n = 30$ cells over three biological replicates). POLGA WT/MITOL FRET were used as the control. (**B**) Small molecules disrupt the interaction between GST MITOL and His-PEO mutants in vitro. In vitro interaction assays were carried out with recombinant soluble His-tagged POLGA WT, PEO 2, PEO 6 and bound GST-tagged MITOL in the absence or presence of C4. (**B**, top) Input of the recombinant proteins. (**B**, bottom) Post-interaction. For both input and post-interaction blots, Western analysis was carried out with the indicated antibodies. Mean ± SD. $n = 4$ biological replicates. (**C**) Small molecules disrupt the interaction between MITOL and PEO mutants in cells. Myc-tagged MITOL was transiently expressed in stable cell lines expressing YFP-HA-tagged POLGA WT or PEO 2 or PEO 6. Cells were either left untreated or treated with the C4 compound. (**D**, Top) The expression of the proteins was checked with the indicated antibodies. (**D**, Bottom) Anti-HA immunoprecipitation was performed on these cell lysates, and immunoblots were carried out with the indicated antibodies. $n = 4$ biological replicates. (**D**, **E**) Small molecules decrease the extent of ubiquitylation on PEO mutants. (Top of both **D**, **E**) Western blotting analysis was performed with the cell extracts of HEK293T cells transiently expressing Flag-tagged POLGA WT or (**D**) PEO 6 or (**E**) PEO 7 after treatment with C1, C2, C3, and C4. Western analysis was done with the indicated antibodies. (Bottom of both **D**, **E**) Immunoprecipitation was performed using anti-Flag beads with the same cell extracts. The extent of POLGA/PEO ubiquitylation was assessed by probing with the indicated antibodies. $n = 4$ biological replicates. (**F**) C4 treatment does not alter MITOL-mediated ubiquitylation of POLGA and RECQL4. (Top) Cell extracts were made from HeLa shGFP and HeLa shMITOL cells grown either without or with C4 treatment. Western blots were carried out with the indicated antibodies. Immunoprecipitation was carried out on the same cell extracts with (Middle) anti-RECQL4 and (Bottom) anti-POLGA antibodies. The extent of ubiquitylation was assessed by probing with anti-ubiquitin (P4D1) and anti-POLGA/RECQL4 antibodies. n = 3 biological replicates. (**G**, **H**) C1 treatment restores mtDNA replication in cells expressing PEO mutants. (**G**) mtDNA was isolated from BrdU-treated HEK293T cells expressing Flag-tagged wild-type POLGA, PEO 5, PEO 6, and PEO 7, which have been grown either with or without C1. mtDNA replication was analysed by measuring the extent of BrdU incorporation using Southwestern blot analysis with an anti-BrdU antibody. EtBr gel was used as a loading control. (**H**) Quantitation of (**G**). $n = 3$ biological replicates. Data represent the mean ± SD and were analysed using ordinary one-way (for **A**) and two-way ANOVA (for **H**) statistical tests with Dunnett's (**A**) and Tukey's (**H**) multiple comparisons tests.

© The Author(s)

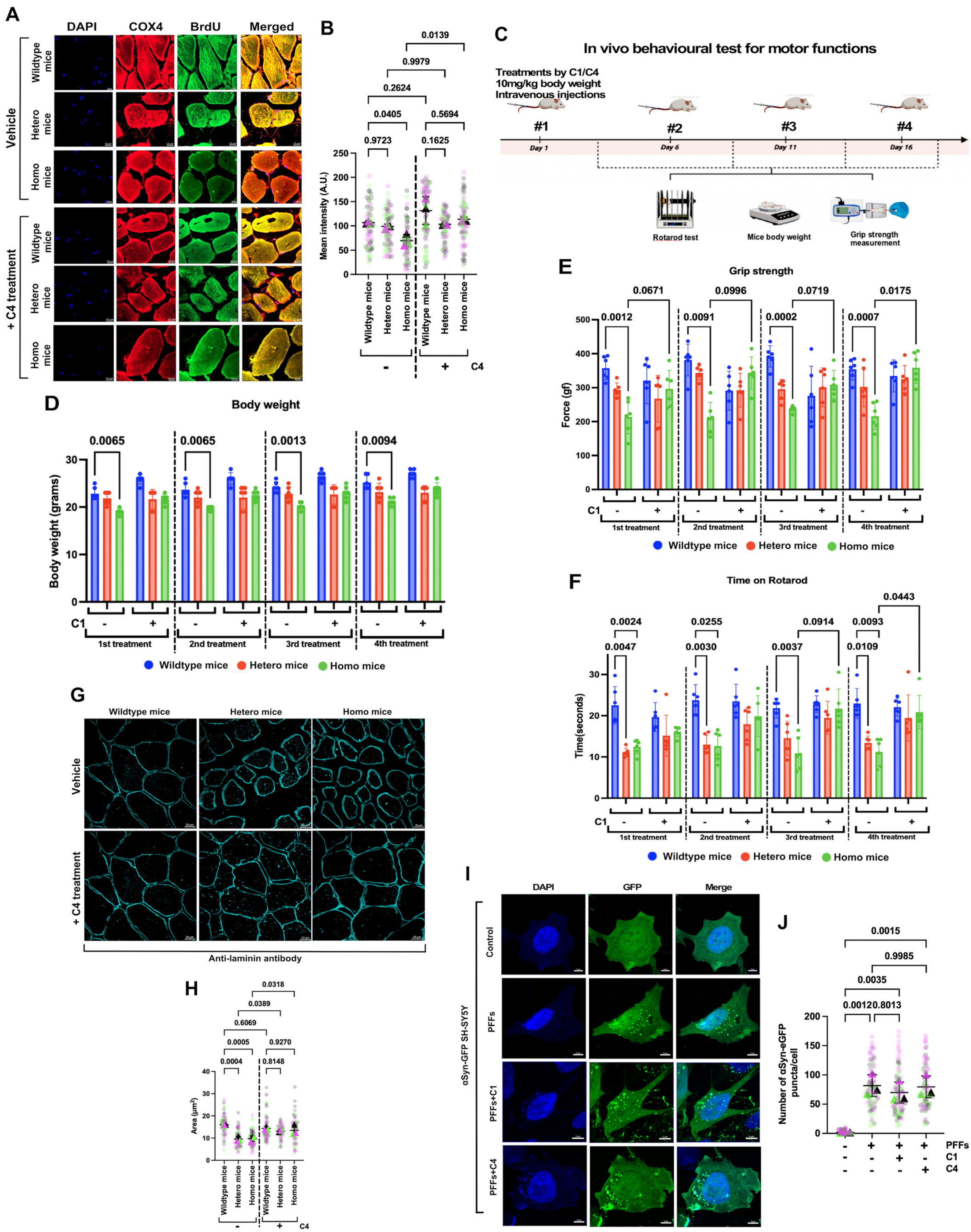
A
DAPI
COX4
BrdU
Merged
Vehicle
Wildtype mice
Hetero mice
Homo mice
+ C4 treatment
B
Mean intensity (A.U.)
C4
C
In vivo behavioural test for motor functions
Treatments by C1/C4 10mg/kg body weight Intravenous injections
Rotarod test
Mice body weight
Grip strength measurement
D
Body weight
Body weight (grams)
1st treatment
2nd treatment
3rd treatment
4th treatment
E
Grip strength
Force (gf)
F
Time on Rotarod
Time(seconds)
G
Anti-laminin antibody
H
Area (μm²)
I
αSyn-GFP SH-SY5Y
Control
PFFs
PFFs+C1
PFFs+C4
GFP
Merge
J
Number of αSyn-eGFP puncta/cell
PFFs
C1

Figure EV5. Treatment with C1 or C4 enhances multiple parameters of mitochondrial functions in the mice model.

(**A**, **B**) C4 treatment restored mtDNA replication in the PEO mice model. (**A**) In vivo BrdU incorporation assay in mice skeletal muscle was analysed using immunofluorescence. Mice skeletal tissue sections from POLGA WT, Hetero ($POLG^{WT/A449T}$), and Homo ($POLG^{A449T/A449T}$) mice were stained with antibodies against BrdU and COX4. (**B**) BrdU signal intensity per muscle fibre was quantitated using SuperPlots. Mean ± SD. $n = 70$ muscle fibres across three biological replicates. (**C**) Schematic diagram depicting how POLGA WT, Hetero ($POLG^{WT/A449T}$) and Homo ($POLG^{A449T/A449T}$) mice were subjected to treatment with small molecules. (**D–F**) C1 treatment restored clinical phenotypes to the basal level in the PEO mice model. Seven-week-old POLGA WT, Hetero ($POLG^{WT/A449T}$) and Homo ($POLG^{A449T/A449T}$) mice were evaluated for clinical phenotypes, including (**D**) Body weight, (**E**) Grip strength and (**F**) Time spent on the rotarod without or after treatment with C1. Measurements were performed on the day following injections for four weeks. Mean ± SD. $n = 6$ mice. (**G**, **H**) C4 treatment enhances muscle fibre areas in Hetero ($POLG^{WT/A449T}$) and Homo ($POLG^{A449T/A449T}$) mice. (**G**) Same as (**A**), except the muscle fibre area was analysed using an antibody against laminin. Scale bar = 20 µm. (**H**) Quantitation of (**G**) using SuperPlots. $n = 70$ muscle fibres over three biological replicates. (**I**, **J**) C1 or C4 treatment do not disrupt α-synuclein aggregate formation in pre-formed fibrils (PFF)-treated αSyn-GFP SH-SY5Y cells. Immunofluorescence followed by confocal microscopy was performed in stably expressing αSyn-GFP SH-SY5Y cells, which were either untreated or treated with PFFs followed by treatment with C1 or C4. The nucleus was stained with DAPI. Scale bar = 5 µm. (**J**) Quantitation of (**I**) using SuperPlots. $n = 84$ cells across 3 biological replicates. Data represent the mean ± SD and were analysed using ordinary one-way (for **B**, **H**, **J**) and mixed-effects analysis (for **D**, **E**, **F**) statistical tests with Tukey's multiple comparisons test.

© The Author(s)
