# Supplementary material for: Small molecules rescue pathogenic POLGA aggregation and restore its functions in a mouse model of PEO
Source: EMBO Mol Med. 2026 Oct 6;18(11):4494–528. doi: 10.1038/s44321-026-00532-3 (PMC13748654; doi:10.1038/s44321-026-00532-3)
Supplement: Supplementary file 3 [file 44321_2026_532_MOESM1_ESM.docx]

**Table EV1: Details of patient mutations used in the study**

| **Identifier (lab references)** | **PEO patient mutation** |
| --- | --- |
| PEO 2 | A467T |
| PEO 5 | T251I |
| PEO 6 | L304R |
| PEO 7 | R943H |
