# Supplementary material for: Small molecules rescue pathogenic POLGA aggregation and restore its functions in a mouse model of PEO
Source: EMBO Mol Med. 2026 Oct 6;18(11):4494–528. doi: 10.1038/s44321-026-00532-3 (PMC13748654; doi:10.1038/s44321-026-00532-3)
Supplement: Supplementary file 4 [file 44321_2026_532_MOESM2_ESM.docx]

**Table EV2: List of small molecules with >95% disruption for PEO aggregates.**

| **Name** | **CAS number** | **Chemical structure** | **EC_50_ Value (nM)** |
| --- | --- | --- | --- |
| Diltiazem hydrochloride (C1) | 33286-22-5 |  | 20.3 ± 1.651 |
| Irinotecan hydrochloride trihydrate (C2) | 136572-09-3 |  | 24.38 ± 1.619 |
| Clorgyline hydrochloride (C3) | 17780-75-5 |  | 12 ± 1.647 |
| Sulbactam (C4) | 68373-14-8 |  | 9.78 ± 1.692 |
| Debrisoquin sulfate (C7) | 581-88-4 |  | 24.63 ± 1.657 |
| Adamantamine fumarate (C8) | 80789-67-9 |  | 27 ± 1.696 |
| Amphotericin B (C9) | 1397-89-3 |  | 36.93 ± 1.712 |
| Sulfamethazine sodium salt (C10) | 1981-58-4 |  | 25.1 ± 1.782 |
| Pyrvinium pamoate (C11) | 3546-41-6 |  | 23.83 ± 1.618 |
