# Supplementary material for: Small molecules rescue pathogenic POLGA aggregation and restore its functions in a mouse model of PEO
Source: EMBO Mol Med. 2026 Oct 6;18(11):4494–528. doi: 10.1038/s44321-026-00532-3 (PMC13748654; doi:10.1038/s44321-026-00532-3)
Supplement: Supplementary file 5 [file 44321_2026_532_MOESM3_ESM.docx]

**Table EV3: Primers used in this study.**

| **Recombinant Name** | **Forward primer sequence (5’ to 3’)** | **Reverse primer sequence (5’ to 3’)** | **References** |
| --- | --- | --- | --- |
| **Oligos for cloning** | | | |
| pLVX-IRES-Hyg-POLGA-YFP-HA | CGG ACT AGT CCG GCC ACC ATG AGC CGC CTG CTC TGGCGG ACT AGT CCG GCC ACC ATG AGC CGC CTG CTC TGG | ATA GTT TAG CGG CCG CAT TCT TAT CTA AGC GTA ATC TGG AAC ATC GTA TGG GTA AGC TCGAGA TCT GAG TCC GG | This study |
| pLVX-IRES-Hyg-POLGA-GFP 11 | CGG ACT AGT CCG GCC ACC ATG AGC CGC CTG CTC TGGCGG ACT AGT CCG GCC ACC ATG AGC CGC CTG CTC TGG | CGC GGA TCC GCG CTA GGT GAT ACC GGC AGC ATT TAC ATA CTC ATG AAG GAC CAT GTG GTC ACG CAT CAT GAA TTTCCC GCC GGA CCC ACC ACT CCC TGG TCC AGG CTG GCT TCG | This study |
| pLVX-YFP-N1 | CGC GGA TCC GCG ATG GTG AGC AAG GGC GAG GA | ATA GTT TAG CGG CCG CAT TCT TAT CTA AGC TCG AGA TCT GAG TCC GG | This study |
| pLVX-MITOL-YFP | CGC GGA TCC GCG GCC ACC ATG CCG GAC CAA GCC CTA CA | CGC GGA TCC GCG TGC TTC TTC TTG TTC TGG AT | This study |
| pLVX-TOM20-YFP | CCG CTC GAG CGG GCC ACC ATG GTG GGT CGG AAC AG | CGC GGA TCC GCG TTC CAC ATC ATC TTC AGC | This study |
| pLVX-IRES-Hyg-CFP-Flag | ATA AGA ATG CGG CCG CTA AAC TAT ATG GTG AGC AAG GGC GAG GA | CGC GGA TCC GCG CTA CTT ATC GTC GTC ATC CTT GTA ATC CTT GTA CAG CTC GTC CAT GC | This study |
| pLVX-IRES-POLGA-CFP-Flag | CGG ACT AGT CCG GCC ACC ATG AGC CGC CTG CTC TG | ATA GTT TAG CGG CCG CAT TCT TAT TGG TCCAGGCTGGCTTC | This study |
| pLVX-IRES-Hyg- YFP-HA | CGG ACT AGT CCG GCC ACC ATG GTG AGC AAG GGC GAG GA | ATA GTT TAG CGG CCG CAT TCT TAT CTA AGC GTA ATC TGG AAC ATC GTA TGG GTA AGC TCGAGA TCT GAG TCC GG | This study |
| **Genotyping PCR primers** | | | |
| FVB POLGA | GTT GTC CCT GTC TTC CTC CA | AAG CTT CCC ACC TTC CTG AT | (Silva-Pinheiro *et al.*, 2021) |
| **Long range mtDNA amplification assay** | | | |
| mtND1 | CAC CCA AGA ACA GGG TTT GT | TGG CCA TGG GTA TGT TGT TAA | (Chi *et al*, 2012) |
| mtDNA | TGA GGC CAA ATA TCA TTC TGA GGG GC | TTT CAT CAT GCG GAG ATG TTG GAT GG |  |
| **mtDNA copy number** | | | |
| Cytochrome b | CCA ACA TCT CCG CAT GAT GAA | TTC AGC CAT AAT TTA CGT CTC | This study |
| β-globin | CTT GAG GTT GTC CAG GTG AGC | AGG CTG CTG GTG GTC TAC CCT |  |
| **Primer extension assay (5’-3’)** | | | |
| 20-mer primer | CAG ATA CTG CGA CAT AGG GT | | (Gupta *et al*, 2014; Hussain *et al.*, 2021) |
| 40-mer template | CGAGACGCTGGAGCCGGAGCACCCTATGTCGCAGTATCTG | |  |
| **Exonuclease assay** | | | |
| 25-mer primer | GCC TCG CAG CCG TCC AAC CAA TGT T | | (Gupta *et al.*, 2014) |
| 45-mer template | GGA CGG CAT TGG ATC GAG GTT GAG TTG GTT GGA CGG CTG CGA GGC | |  |
